# Association Between Folate and Health Outcomes: An Umbrella Review of Meta-Analyses

**DOI:** 10.3389/fpubh.2020.550753

**Published:** 2020-12-15

**Authors:** Yacong Bo, Yongjian Zhu, Yuchang Tao, Xue Li, Desheng Zhai, Yongjun Bu, Zhongxiao Wan, Ling Wang, Yuming Wang, Zengli Yu

**Affiliations:** ^1^School of Public Health, Xinxiang Medical University, Xinxiang, China; ^2^Department of Cardiology, The First Affiliated Hospital of Zhengzhou University, Zhengzhou, China; ^3^School of Public Health, Zhengzhou University, Zhengzhou, China; ^4^Centre for Population Health Sciences, University of Edinburgh, Edinburgh, United Kingdom; ^5^Department of Administration, Henan University People's Hospital, Zhengzhou, China

**Keywords:** folate, meta-analysis, umbrella review, multiple health outcomes, chronic diseases

## Abstract

**Background:** There is no study that has systematically investigated the breadth and validity of the associations of folate and multiple health outcomes. We aimed to evaluate the quantity, validity, and credibility of evidence regarding associations between folate and multiple health outcomes by using umbrella review of meta-analysis.

**Methods:** We searched the MEDLINE, EMBASE, and Cochrane Library databases from inception to May 20, 2018, to identify potential meta-analyses that examined the association of folate with any health outcome. For each included meta-analysis, we estimated the summary effect size and their 95% confidence interval using the DerSimonian and Laird random-effects model. We used the AMSTAR 2 (A Measurement Tool to Assess Systematic Reviews) to assess methodological quality and the GRADE (Grading of Recommendations, Assessment, Development, and Evaluation working group classification) to assess the quality of evidence for each outcome included in the umbrella review.

**Results:** Overall, 108 articles reporting 133 meta-analyses of observational studies and 154 meta-analyses of randomized controlled trials (RCTs) were included in the study. Among them, 108 unique exposure–outcome–population triplets (referred to as unique meta-analyses hereafter) of RCTs and 87 unique meta-analyses of observational studies were reanalyzed. Beneficial effects of folate were observed in the all-cause mortality rate and in a number of chronic diseases, including several birth/pregnancy outcomes, several cancers, cardiovascular disease and metabolic-related outcomes, neurological conditions, and several other diseases. However, adverse effects of folate were observed for prostate cancer, colorectal adenomatous lesions, asthma or wheezing, and wheezing as an isolated symptom and depression.

**Conclusions:** Current evidence allows for the conclusion that folate is associated with decreased risk of all-cause mortality and a wide range of chronic diseases. However, folate may be associated with an increased risk of prostate cancer. Further research is warranted to improve the certainty of the estimates.

## Introduction

Folate, which mediates the transfer of one-carbon units in methylation and biosynthesis of nucleotides, has been well-established to play important roles in the processes of DNA synthesis, stability, repair, and methylation (1). It has been known for more than 2 decades that folic acid supplements during a woman's pregnancy can reduce the risk of neural tube malformation. Since then, numerous studies have investigated the effects of folate on a wide range of health outcomes, including the all-cause and cause-specific mortality, cancer outcomes, cardiovascular disease (CVD), diabetes, neurocognitive disorders, and pregnancy and birth outcomes. It is also noteworthy that folate supplement has been becoming popular worldwide, although evidence regarding the associations between folate and various health outcomes is still inconclusive.

Given this, a systematic assessment of the credibility of the published evidence will provide important implications for folate in both clinical practice and public health. Previous original or meta-analysis studies of the health effects of folate usually focused on a single health outcome (e.g., neural tube malformation). We therefore carried out the current umbrella review of existing published data on the associations between folate exposure and diverse health outcomes. In addition, we aimed to describe the magnitude, direction, and significance of the suggested associations; evaluate the potential biases; and identify which studies produced the highest-quality evidence.

## Methods

### Structure of Umbrella Review

The umbrella review method, which synthesizes information from meta-analyses both of observational studies and randomized controlled trials (RCTs) on multiple health outcomes associated with a particular exposure, could provide an instructive panorama for public health interventions (2, 3). We conducted this umbrella review of folate and multiple health outcomes by systematically searching for meta-analyses in which folate was part or all of the exposure of interest. Meanwhile, we excluded those systematic reviews without meta-analyses.

### Search Strategy

The MEDLINE, EMBASE, and Cochrane Library databases were searched from inception to May 20, 2018 to identify meta-analyses that examined the association between folate and any health outcome. The detailed search strategies are presented in Supplementary Table 1. The titles, abstracts, and full texts of potentially eligible articles were screened by two researchers independently. Disagreements were arbitrated by a third researcher.

### Eligibility Criteria

Articles with meta-analyses were included if they met the following inclusion criteria:

1. Meta-analyses of either observational (i.e., cohort, case-control, and cross-sectional studies) or interventional studies (i.e., RCTs)
2. Evaluating the association of folate (folate intake, folate supplementation, and folate concentration) with any health outcome
3. The included population aged 18 years or older
4. Published in peer-reviewed journals in English.

We excluded meta-analyses that evaluated the effects of genetic polymorphisms related to folate metabolism on health outcomes, animal research, and laboratory studies. If an article presented separate meta-analysis for more than one health outcome, we included each of these separately. For meta-analyses of observational studies, if more than one meta-analysis addressed the same research question, the one with the largest number of prospective cohort studies was included.

### Data Extraction

Two investigators independently extracted information from eligible meta-analyses. For each meta-analysis, the following information was extracted: first author's last name, year of publication, number of studies included, populations, health outcomes of interest, study designs, exposure of folate, effect sizes [odds ratio (OR), risk ratio (RR), hazard ratio (HR), or mean difference (MD)], and the corresponding 95% confidence intervals (CIs), and types of effect model used in the meta-analysis (fixed or random). In addition, we also extracted number of cases and controls (for case–control studies), events and participant/person-years (for cohort studies), or number of subjects in interventional and control groups (for RCTs). For each original study included in each meta-analysis, the following data were extracted for further reevaluation: the effect estimates (OR, RR, HR, or MD) with 95% CI, number of cases, total number of participants, and study design.

### Data Analysis

Summary effects and 95% CIs for each meta-analysis were reanalyzed by using a DerSimonian and Laird random-effects model to be consistent with the method widely used in the included meta-analyses. For any associations with *p* < 0.05, the following metrics were further estimated: the 95% prediction interval to evaluate the uncertainty for the effect that would be expected in a new original study (4, 5); the between-study heterogeneity (defined as significant for *I*^2^ ≥ 50% and *p* < 0.05); the excess significance test to assess whether the observed number (O) of studies with significant results (positive studies) was larger than the expected number (E) (6); and the presence of small-study effect by using Egger regression asymmetry test (significance threshold *p* < 0.10) (7).

For overlapping outcomes that were examined both in meta-analyses of RCTs and those of observational studies, we examined whether the observed direction and statistical significance were consistent between the two study types.

### Assessment of Methodological Quality

We used the updated AMSTAR 2 (A Measurement Tool to Assess Systematic Reviews) to evaluate the methodological quality of the included meta-analyses. Compared with the original AMSTAR tool, the AMSTAR 2 emphasizes the risk-of-bias assessment in study design and heterogeneity and is a reliable and valid tool for quality assessment of meta-analyses of both interventional and observational research (8). The AMSTAR 2 includes 16 items for evaluating the methodological quality of systematic reviews/meta-analyses, with each item scoring 0 or 1. The methodological quality of each individual meta-analysis was then classified as high, moderate, low, or critically low accordingly.

### Credibility of the Evidence

The Grading of Recommendations, Assessment, Development, and Evaluation working group classification (GRADE) was used to assess the quality of evidence for those meta-analyses included in the umbrella review (9, 10). The GRADE categorizes evidence from systematic reviews and meta-analyses into the levels of high, moderate, low, or very low. In the GRADE approach, RCTs start as high-quality evidence, and observational studies start as low-quality evidence. Other factors may then upgrade or downgrade the quality level. For example, unexplained heterogeneity or high probability of publication bias may downgrade the quality of evidence, whereas a large effect or dose-response gradient may upgrade it. Two reviewers independently assessed the included studies, and a third reviewer settled disagreements.

## Results

### Literature Review

**Figure 1:**
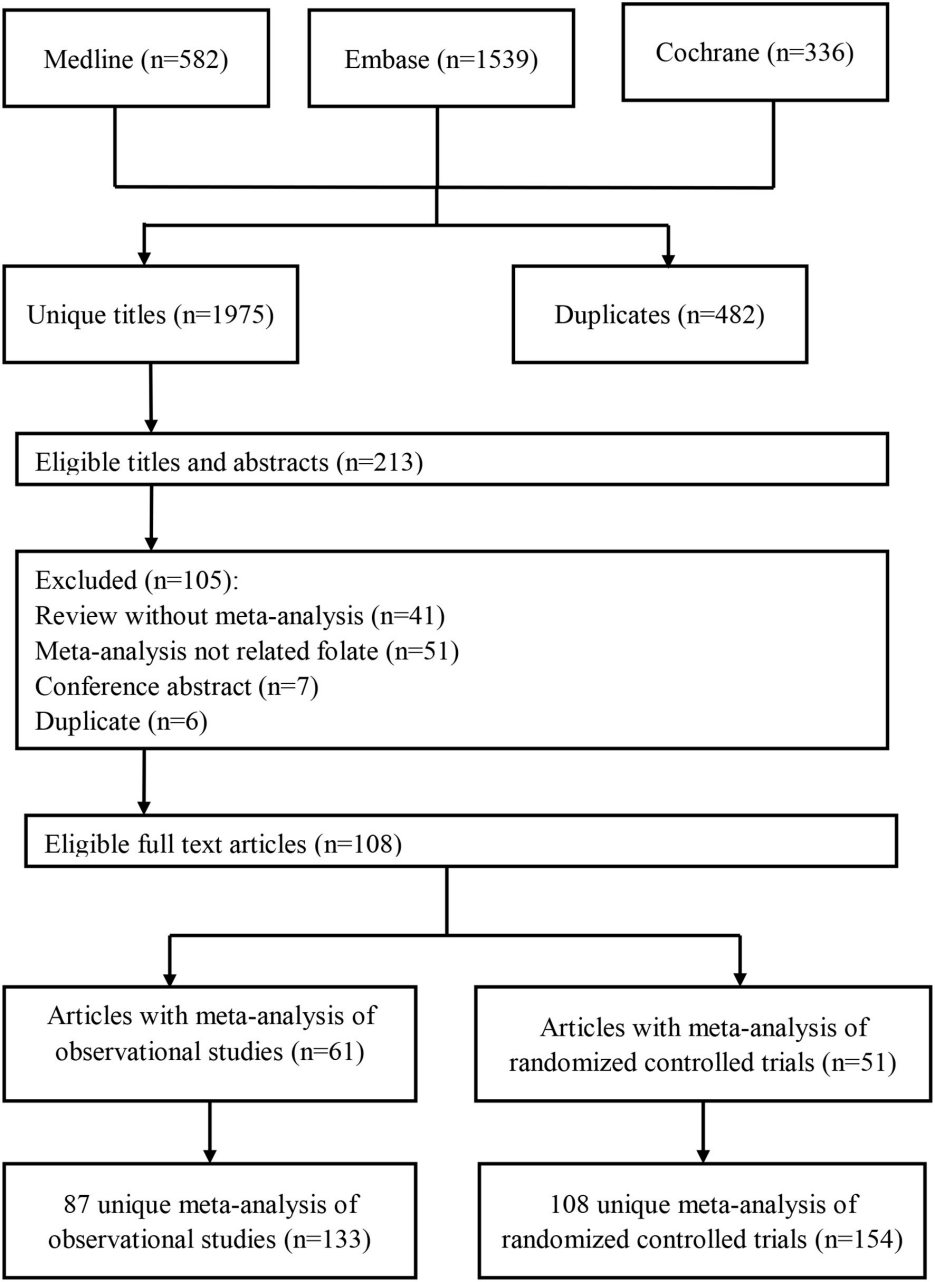
Flowchart of selection of studies for inclusion in umbrella review on folate and health.

The flow of study selection is presented in Figure 1. We initially identified 1,975 unduplicated articles. After considering the inclusion and exclusion criteria, 108 articles were finally included in the study. Among them, 133 meta-analyses of observational studies were reported in 62 articles (11–72), and 154 meta-analyses of RCTs were reported in 51 articles (13, 20, 28, 47, 62, 73–118). Another five articles reported both meta-analyses with observational studies and RCTs (13, 20, 28, 47, 62). As a result, a total of 195 unique health outcomes classified into eight health fields (i.e., all-cause and cause-specific mortality rates, cancer outcomes, cardiovascular outcomes, birth outcomes, pregnancy outcomes, neurocognitive disorders, and other outcomes) were reported (Supplementary Figure 1).

### Meta-Analysis of Observational Studies

As shown in Supplementary Table 2, the median number of meta-analyses with observational studies included in each outcome was 7 (range, 2–36), and the median numbers of participants/case numbers were 43,063 (range, 635–59,514,473) and 3,463 (range, 11–147,424), respectively. Twenty-one outcomes were reported in more than one meta-analysis.

**Figure 2:**
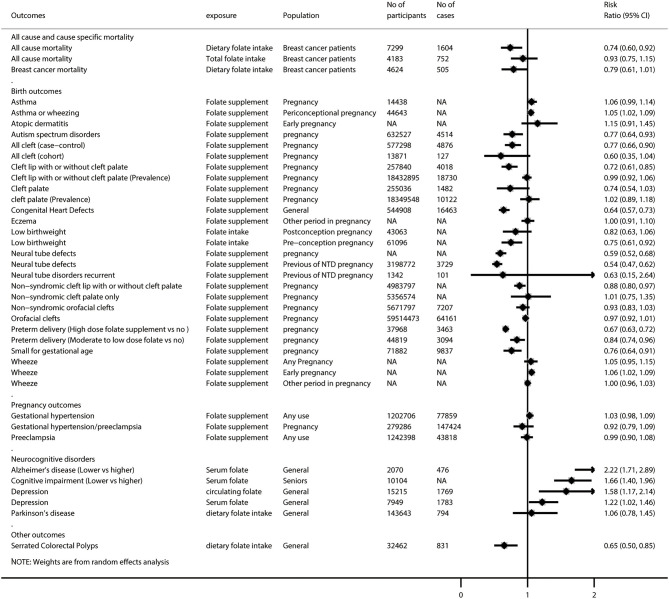
Summary random-effects estimates of cancer outcomes and cardiovascular outcomes reported in meta-analyses of observational studies.

**Figure 3:**
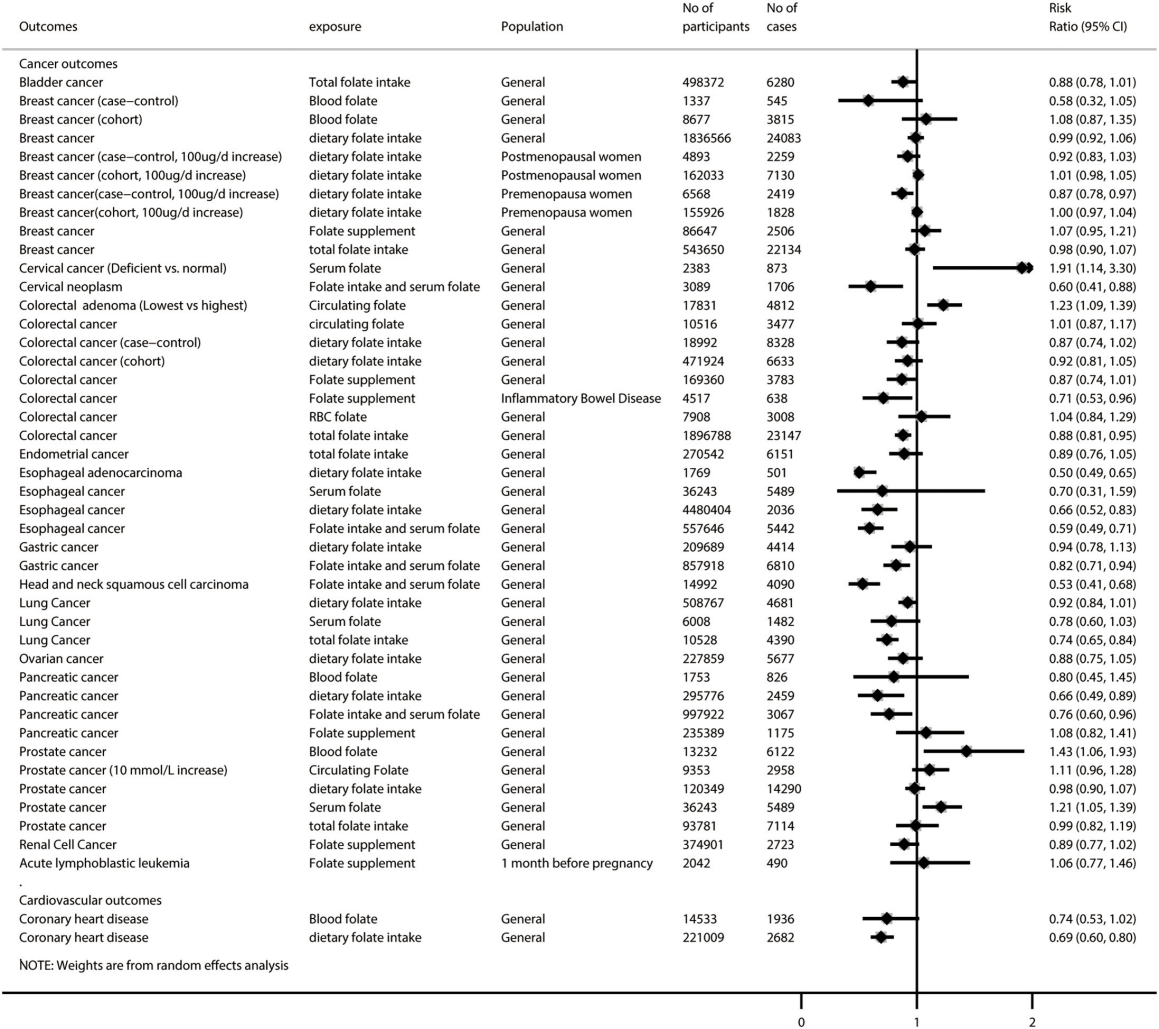
Summary random-effects estimates of all-cause and cause-specific mortality, birth outcomes, pregnancy outcomes, neurocognitive disorders, and other outcomes reported in meta-analyses of observational studies.

After excluding 46 duplicated meta-analyses, we further analyzed 87 unique exposure–outcome–population triplets (referred to as unique meta-analyses hereafter) of observational studies with a wide range of outcomes (Supplementary Table 3): all-cause and cause-specific mortality (*n* = 3), birth outcomes (*n* = 28), cancer-related outcomes (*n* = 45), cardiovascular outcomes (*n* = 2), neurocognitive disorders (*n* = 5), pregnancy outcomes (*n* = 3), and other outcomes (*n* = 1). Figures 2, 3 show the summarized results of these 87 unique meta-analyses. Overall, 35 of the 87 (40.2%) meta-analyses reported nominally significant pooled results (*p* < 0.05).

Of these 87 unique meta-analyses, 10 (11.5%) were with statistical significance of *p* < 10^−6^, 7 (8.0%) had a 95% prediction interval excluding the null, 60 (69.0%) had more than 1,000 cases (or more than 20,000 participants for continuous outcomes), 16 (18.4%) had neither evidence of excess significance bias (*p* > 0.10) nor small-study effects (*p* > 0.10), and 40 (46.0%) had no large heterogeneity (*I*^2^ < 50% and *p* > 0.05).

Supplementary Table 4 provides a breakdown of the AMSTAR 2 scores for the meta-analyses representing each outcome. None of the 87 meta-analyses was rated at the high methodological level, and 3 (3.4%) were rated as moderate, leaving 31 (35.6%) as low and 53 (60.9%) as critically low. Regarding the GRADE classification for evidence level, 4 of the 87 meta-analyses (4.6%) were rated as high-quality evidence for the corresponding outcomes, 21 (24.1%) were rated as moderate, 12 (13.8%) were rated as low, and 50 (57.5%) were rated as very low quality (Supplementary Table 5).

### Data Synthesis for High- or Moderate-Quality Meta-Analysis of Observational Studies

Among the 25 meta-analyses with high or moderate GRADE classification, we found that folate intake was associated with lower risks of low birth weight (during preconception), esophageal adenocarcinoma, gastric cancer, head and neck squamous cell carcinoma, pancreatic cancer, coronary heart disease, and serrated colorectal polyps (among adults undergoing endoscopic investigation of the colorectal), but it did not show a significant association with low birth weight (during post-conception pregnancy), colorectal cancer, lung cancer, and Parkinson disease. Folate supplementation was associated with lower risks of non-syndromic cleft lip with or without cleft palate and small for gestational age, but it did not show a significant association with non-syndromic cleft palate, wheezing, acute lymphoblastic leukemia, and gestational hypertension/preeclampsia. A higher level of circulating folate was associated with lower risks of cervical cancer, colorectal adenoma, and Alzheimer disease, but it did not show a significant association with lung cancer and coronary heart disease (Supplementary Figure 2). Interestingly, we found that circulating folate did not show a significant association with prostate cancer, but higher serum folate was associated with increased risk of prostate cancer.

### Meta-Analyses of RCTs

As shown in Supplementary Table 6, the median number of meta-analyses of RCTs included in each outcome was 5.5 (range, 2–25), and the median numbers of participants and cases were 3,113 (range, 28–82,723) and 653 (range, 3–39,923), respectively. More than one meta-analysis was reported for 17 outcomes.

**Figure 4:**
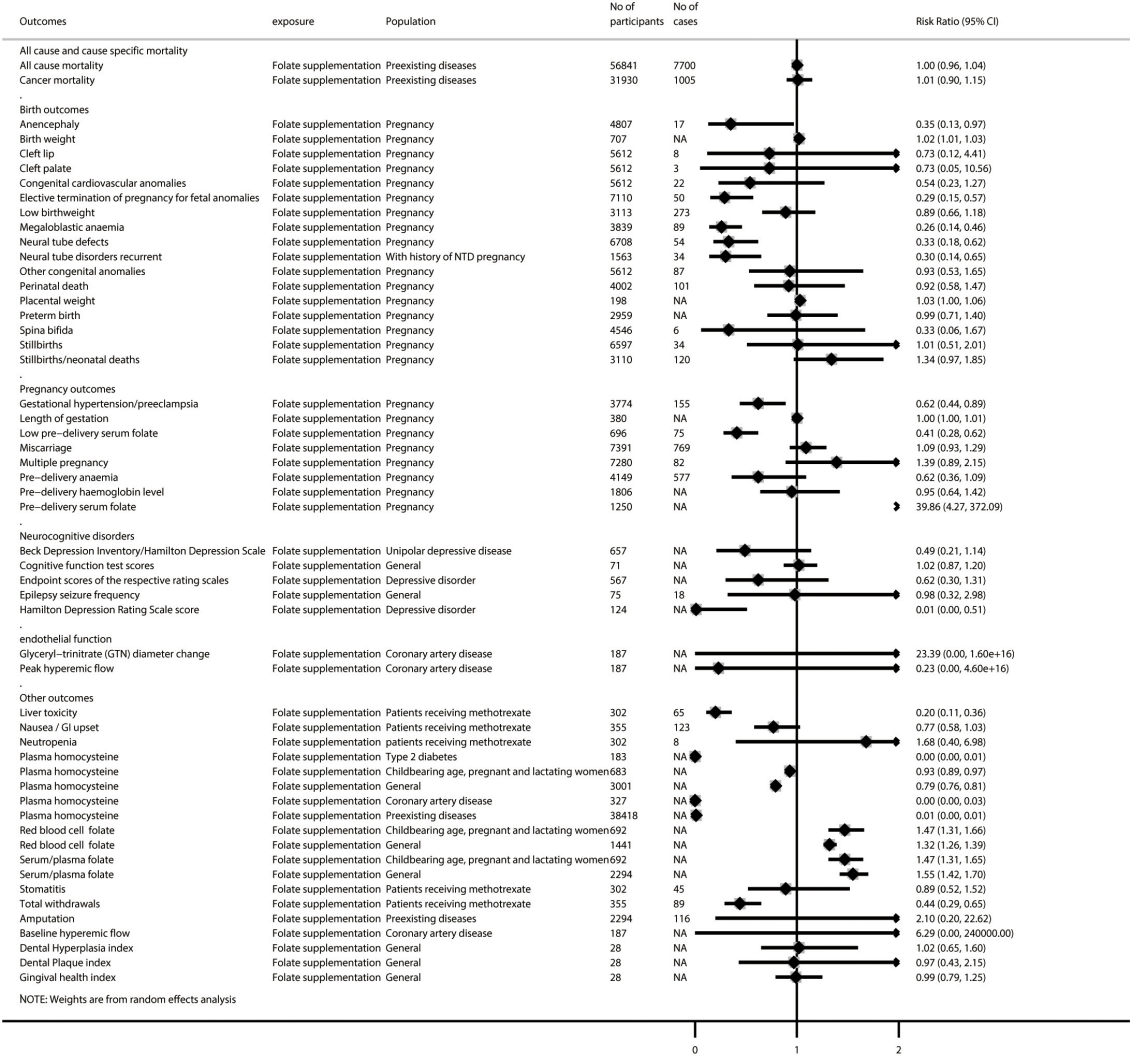
Summary random-effects estimates of all-cause and cause-specific mortality, birth outcomes, pregnancy outcomes, neurocognitive disorders, endothelial function, and other outcomes reported in meta-analyses of randomized controlled trials.

**Figure 5:**
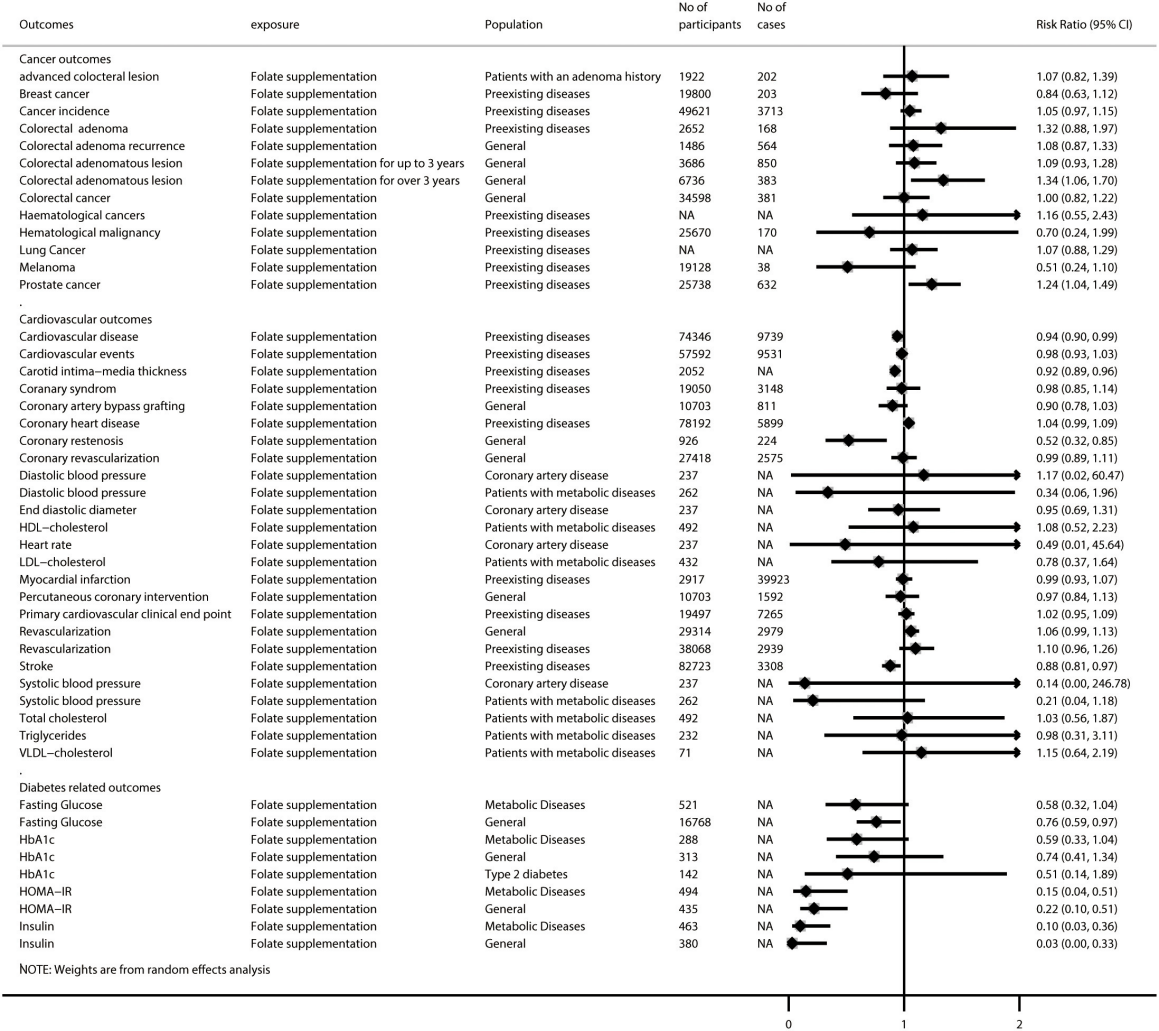
Summary random-effects estimates of cancer outcomes, cardiovascular outcomes, and diabetes-related outcomes reported in meta-analyses of randomized controlled trials.

After removing 46 duplicated meta-analyses, we further analyzed 108 unique meta-analyses of RCTs for the associations of folate with all-cause and cause-specific mortality (*n* = 2), birth outcomes (*n* = 17), cancer-related outcomes (*n* = 14), cardiovascular outcomes (*n* = 29), diabetes-related outcomes (*n* = 9), endothelial function (*n* = 5), neurocognitive disorders (*n* = 5), pregnancy outcomes (*n* = 8), and other outcomes (*n* = 19). The summarized results of these 108 unique meta-analyses are presented in Figures 4, 5. Overall, 31 (28.7%) meta-analyses showed nominally significant pooled results (*p* < 0.05). Among the 31 meta-analyses, 6 were for birth outcomes, 3 were for cardiovascular outcomes, 5 were for diabetes-related outcomes, 1 was for neurocognitive disorders, 3 were for pregnancy outcomes, and 11 were for other outcomes, suggesting that folate supplementation was associated with a decreased risk of these aforementioned diseases. However, two meta-analyses for cancer-related outcomes reported pooled results with *p-*values lower than 0.05, suggesting that folate supplementation was associated with increased risks of colorectal adenomatous lesion and prostate cancer.

As shown in Supplementary Table 7, 25 of the 108 meta-analyses (23.1%) showed statistical significance (*p* < 0.01); the 95% prediction interval excluded the null in 6 (5.6%), 14 (13.0%) had more than 1,000 cases (or more than 20,000 participants for continuous outcomes), 8 (7.4%) had no evidence of excess significance bias (*p* > 0.10) or small-study effects (*p* > 0.10), and 49 (45.4%) showed no great heterogeneity (*I*^2^ < 50% and *p* > 0.05).

Supplementary Table 8 presents a breakdown of the AMSTAR 2 scores for the meta-analyses representing each outcome. None of the 108 meta-analyses was rated at a high methodological level, and 14 (13.0%) were rated as moderate, leaving 36 (33.3%) as low and 58 (53.7%) as critically low. In terms of evidence quality for each outcome, 10 of the 108 meta-analyses (9.3%) were rated as high, 24 (22.2%) were rated as moderate, 22 (20.4%) were rated as low, and 52 (48.1%) were rated as very low quality by the GRADE classification (Supplementary Table 9).

### Data Synthesis for High- or Moderate-Quality Meta-Analyses of RCTs

Among the 34 meta-analyses with high or moderate GRADE classification, we found that folate supplementation was associated with decreased risk of elective termination of pregnancy for fetal anomalies; megaloblastic anemia; neural tube defects; CVD (among those with preexisting diseases); liver toxicity (patients receiving methotrexate); gestational hypertension/preeclampsia; low predelivery serum folate; decreased scores on the Hamilton Depression Rating Scale and levels of plasma homocysteine (both among patients with type 2 diabetes and the general population); increased levels of birth weight, red blood cell folate, and serum/plasma folate; and increased risk of prostate cancer (among those with preexisting diseases). However, we did not find any significant association between folate supplementation and the all-cause mortality rate (among those with preexisting diseases), cancer mortality rate (among those with preexisting diseases), low birth weight, preterm birth, stillbirths/neonatal deaths, cancer incidence (among those with preexisting diseases), colorectal adenomatous lesion, colorectal cancer, coronary artery bypass grafting, diastolic blood pressure (among patients with coronary artery disease), end-diastolic diameter (among patients with coronary artery disease), myocardial infarction (among those with preexisting diseases), amputation, gingival health index, miscarriage, or multiple pregnancy (Supplementary Figure 3).

### Comparison Findings in Meta-Analysis of Observational Studies and Those of RCTs

One hundred eighty (92.3%) unique meta-analyses examined only observational studies (*n* = 77) or RCTs (*n* = 103), so the evidence from those meta-analyses could not be compared between observational and randomized studies.

Five outcomes from 15 meta-analyses were investigated by meta-analyses of both observational studies (*n* = 10) and RCTs (*n* = 5) (Supplementary Table 10): cleft palate, neural tube defects, recurrence of neural tube defects, colorectal cancer, and gestational hypertension/preeclampsia. Between meta-analyses of observational studies and those of RCTs, the direction of the association/effect and level of statistical significance were concordant for cleft palate, neural tube defects, and the effects of different folate exposure [dietary folate intake (both case–control and cohort studies), red blood cell folate, circulating folate, and folate supplementation] on colorectal cancer. The direction of the association/effect but not the level of statistical significance was concordant for gestational hypertension/preeclampsia and the recurrence of neural tube defects among women with a previous pregnancy with indicators of neural tube defects. In addition, the pooled results of the effect of total folate intake on colorectal cancer from observational studies were also discordant with those from RCTs both in direction and the level of significance.

## Discussion

In this study, we first provided an overview and appraisal of the relationships between folate exposure and a wide range of health outcomes. We found that folate is more often associated with benefit than harm for a range of health outcomes across multiple measures of exposure, including folate intake, folate supplementation, and folate concentration. Overall, we observed the beneficial effects of folate intake/level/supplementation on all-cause mortality and a number of chronic diseases, including cancers, CVD, and metabolic-related outcomes, as well as several birth outcomes. However, adverse effects of supplemented/serum folate were observed on prostate cancer, colorectal adenomatous lesion, asthma or wheezing, and wheezing as an isolated symptom.

The beneficial effects of folate on the aforementioned health outcomes might be explained by a number of plausible mechanisms. First, folate is the cofactor for methionine synthase, which catalyzes the conversion of homocysteine, and folate levels are therefore inversely associated with homocysteine levels (119, 120). Hyperhomocysteinemia has been found to be associated with higher risks of some birth/pregnancy outcomes (121–123), cancers (124–126), CVD (127), and neurological conditions (128, 129). Second, the polymorphisms of 5,10-methylenetetrahydrofolate reductase, which are critical junctions in the folate-metabolizing pathway via their role of guiding folate metabolites to the DNA methylation pathway and away from the DNA synthesis pathway, may modulate the susceptibility of subjects to several birth/pregnancy outcomes (130–132), cancers (133, 134), CVD (135), and neurological conditions (136).

The evidence on the association of folate with the all-cause mortality rate in the general population remains controversial. Several studies have demonstrated that folate supplementation could reduce the risk of CVD-related death, which might be attributable to serum homocysteine reduction (137). In contrast, Ebbing et al. reported that folate treatment was associated with increased risks of cancer outcomes and all-cause mortality in patients with ischemic heart disease (138). Excess folic acid intake may stimulate the growth of established neoplasms in experimental animals (139). As such, establishing the appropriate range of folate dosage might be crucial to balance the benefits against the risks and allow us to more accurately study the associations of folate with all-cause or cause-related mortality.

Other than the reasons mentioned above, the associations between folate and cancers may also be explained by two further mechanisms: (1) folate deficiency may induce complete transformation of deoxyuridylate monophosphate to deoxythymidylate monophosphate, which induces mis-incorporation of uracil into DNA and leads to chromosomal breaks and mutations (140, 141); and/or (2) folate deficiency may cause abnormal methylation of DNA, leading to alterations in expression of critical protooncogenes and tumor suppressor genes (142, 143). Experiments *in vivo* on mice and dogs have suggested that increased folate intake altered DNA methylation and in turn reduced the risks of cancers (144, 145).

Studies have suggested that folate can also prevent and reverse endothelial dysfunction (146, 147), which is an important risk factor for CVD (148, 149). Folate may improve the bioavailability of nitric oxide (NO) by increasing endothelial NO synthase coupling and NO production and by directly scavenging superoxide radicals (150, 151). By enhancing NO bioavailability, folate may improve endothelial function, thereby preventing or reversing the progression of CVD (147).

In contrast to its many beneficial effects, we observed adverse effects of folate on prostate cancer, colorectal adenomatous lesion, asthma or wheezing, and wheezing as an isolated symptom. For the adverse effect of folate on increased risk of prostate cancer/asthma, we found that the significant associations were both driven by one individual study. And the removal of these two influential studies from the respective meta-analysis resulted in non-significant results. However, the assessment of New Castle–Ottawa scale suggested that both of these two studies were with low risks of bias (i.e., scored 7–9 out of 10, data not shown). We thus speculate that the inconsistent findings across the included studies may be ascribed to the heterogeneity of population and study design. Further meta-analyses with larger sample size are warranted to verify these associations. For the association between folate and increased risk of colorectal adenomatous lesion, the most likely explanation is that undiscovered early precursor lesions might have existed in the mucosa of these patients, and folate could have accelerated the proliferation and growth of these paraneoplastic lesions.

We found high-quality evidence that folate supplementation was associated with a lower risk of several birth/pregnancy outcomes (neural tube defects, megaloblastic anemia, elective termination of pregnancy for fetal anomalies, small for gestational age, non-syndromic cleft lip with or without cleft palate, gestational hypertension/preeclampsia, and low predelivery serum folate), decreased scores on the Hamilton Depression Rating Scale (in a population with depressive disorder) and levels of plasma homocysteine, and increased serum/plasma folate. Although the meta-analyses of these outcomes might still be subject to potential biases, such as those without a preregistered protocol and the presence of high heterogeneity (for outcomes of small for gestational age and serum/plasma folate), our results are encouraging enough to verify the recommendation that women of child-bearing age should take folate supplementation to prevent adverse birth/pregnancy outcomes.

We found moderate-quality evidence from meta-analyses of observational studies that serum folate was associated with a higher risk of prostate cancer, which is consistent with the high-quality evidence from meta-analyses of RCT that folate supplementation was associated with increased risk of prostate cancer. The potential mechanism of folate in the development of this cancer is unclear. *In vitro* models using human prostate tissue have shown enhanced proliferation of tumor cells under conditions of elevated folate concentrations (152). Elsewhere, mice with transgenic adenoma of mouse prostate (TRAMP) that were fed a folate-depleted diet had lower cellular proliferation than mice with TRAMP fed a normal or high-folate diet (153).

In this umbrella review, the specific trends of relationships between folate and increased risks of neurocognitive disorders (such as cognitive impairment, Alzheimer disease, and depression) were also observed. The proposed mechanisms through which folate affects these diseases include suppression of DNA methylation and reduction of tetrahydrobiopterin levels, hyperhomocysteinemia, and excessive mis-incorporation of uracil into DNA (154). In contrast to the evidence that folate supplementation reduced the risk of stroke, the effect of folate on improving cognitive function or slowing cognitive decline in healthy or cognitively impaired older individuals was inconclusive (155). Prospective studies are strongly warranted to cover this knowledge gap.

### Strengths and Limitations

This umbrella review has several strengths. First, we are the first to summarize the evidence for the associations between folate intake/levels and a wide range of health-related outcomes by incorporating information from published meta-analyses of observational studies or RCTs. Second, we used systematic methods that included a robust search strategy of three scientific literature databases and independent study selection and extraction by two investigators. When possible, we repeated each meta-analysis with a standardized approach that included the use of random-effects analysis and produced measures of heterogeneity and publication bias to allow better comparison across outcomes. We also used standard approaches to assess the quality of methods (AMSTAR 2) and the quality of evidence (GRADE) of the included meta-analyses.

Our study should also be interpreted cautiously with several limitations. First, the credibility assessment method was based on established tools for observational evidence, which are susceptible to bias and uncertainty. Another limitation of the umbrella review approach is the use of existing meta-analyses. Meta-analyses are known to have important limitations, such as limited coverage of the literature search, quality of included studies, and selective outcome reporting.

## Conclusions

Our umbrella review found high- and moderate-quality evidence for the effect of folate on health outcomes such as mortality, cancers, CVD, and metabolic-related outcomes, as well as several birth outcomes. Therefore, our results support the current recommendation of daily folate supplementation for preventing adverse birth/pregnancy outcomes, cardiovascular and metabolic disease, and other disease. Further RCTs with large sample sizes are warranted to confirm these observed findings and to study the concentration–response relationships between folate exposure and health outcomes.

## Data Availability Statement

The original contributions presented in the study are included in the article/Supplementary Material, further inquiries can be directed to the corresponding author/s.

## Author Contributions

ZY was the project lead for the current study. YBo and YZ searched databases and screened the articles. YBo and YT extracted the data. YBo, XL, and YZ conducted statistical analysis. YBo wrote the manuscript. DZ, YBu, ZW, LW, and ZY reviewed and revised the manuscript. All authors contributed to the article and approved the submitted version.

## Conflict of Interest

The authors declare that the research was conducted in the absence of any commercial or financial relationships that could be construed as a potential conflict of interest.
