# Supplementary material for: Association Between Folate and Health Outcomes: An Umbrella Review of Meta-Analyses
Source: Front Public Health. 2020 Dec 15;8:550753. doi: 10.3389/fpubh.2020.550753 (PMC7770110; doi:10.3389/fpubh.2020.550753)
Supplement: Supplementary file 1 [file Table_1.DOCX]

**Association Between Folate and Health Outcomes: An Umbrella Review of Meta-Analysis**

*Yacong Bo^1#^, Yongjian Zhu^2#^, Yuchang Tao^3^, Xue Li^1,4^, Desheng Zhai^1^, Yongjun Bu^1^, Yuming Wang^5^, Zengli Yu^1*^*

*^1^School of Public Health, Xinxiang Medical University, Xinxiang, China,*

*^2^Department of Cardiology, The First Affiliated Hospital of Zhengzhou University, Zhengzhou, China,*

*^3^School of Public Health, Zhengzhou University, Zhengzhou, China,*

*^4^Centre for Population Health Sciences, University of Edinburgh, Edinburgh EH8 9AG, UK,*

*^5^Henan University People's Hospital, Zhengzhou, China.*

^#^These authors contributed equally to this work.

## Supplementary Methods

### AMSTAR Checklist 2

1. Did the research questions and inclusion criteria for the review include the components of PICO?

The research questions and inclusion criteria for the review should include Population, Intervention, Comparator group, and Outcome.

Note: Timeframe for follow-up is optional (recommended) to get a yes.

2. Did the report of the review contain an explicit statement that the review methods were established prior to the conduct of the review and did the report justify any significant deviations from the protocol?

For Partial Yes:

The authors state that they had a written protocol or guide that included ALL the following: review question(s), a search strategy, inclusion/exclusion criteria, a risk of bias assessment.

For Yes:

As for partial yes, plus the protocol should be registered and should also have specified: a meta-analysis/synthesis plan, if appropriate, and a plan for investigating causes of heterogeneity, justification for any deviations from the protocol.

3. Did the review authors explain their selection of the study designs for inclusion in the review?

For Yes, the review should satisfy ONE of the following: explanation for including only RCTs, OR explanation for including only NRSI, OR explanation for including both RCTs and NRSI.

4. Did the review authors use a comprehensive literature search strategy?

For Partial Yes (ALL the following): searched at least 2 databases (relevant to research question), provided key word and/or search strategy, justified publication restrictions (eg, language). For Yes, should also have (all the following): searched the reference lists/bibliographies of included studies, searched trial/study registries, included/consulted content experts in the field, where relevant, searched for grey literature, conducted search within 24 months of completion of the review.

5. Did the review authors perform study selection in duplicate?

For Yes, either ONE of the following: at least two reviewers independently agreed on selection of eligible studies and achieved consensus on which studies to include, OR two reviewers selected a sample of eligible studies and achieved good agreement (at least 80 per cent), with the remainder selected by one reviewer.

6. Did the review authors perform data extraction in duplicate?

For Yes, either ONE of the following: at least two reviewers achieved consensus on which data to extract from included studies, OR two reviewers extracted data from a sample of eligible studies and achieved good agreement (at least 80 per cent), with the remainder extracted by one reviewer.

7. Did the review authors provide a list of excluded studies and justify the exclusions?

For Partial Yes: provided a list of all potentially relevant studies that were read in full text form but excluded from the review

For Yes, must also have: justified the exclusion from the review of each potentially relevant study.

8. Did the review authors describe the included studies in adequate detail?

For Partial Yes (ALL the following): described populations, described interventions, described comparators, described outcomes, described research designs.

For Yes, should also have ALL the following: described population in detail, described intervention and comparator in detail (including doses where relevant), described study’s setting, timeframe for follow-up.

9. Did the review authors use a satisfactory technique for assessing the risk of bias (RoB) in individual studies that were included in the review?

RCTs

For Partial Yes, must have assessed RoB from unconcealed allocation, and lack of blinding of patients and assessors when assessing outcomes (unnecessary for objective outcomes such as all-cause mortality).

For Yes, must also have assessed RoB from: allocation sequence that was not truly random, and selection of the reported result from among multiple measurements or analyses of a specified outcome.

NRSI

For Partial Yes, must have assessed RoB: from confounding, and from selection bias.

For Yes, must also have assessed RoB: methods used to ascertain exposures and outcomes, and selection of the reported result from among multiple measurements or analyses of a specified outcome.

10. Did the review authors report on the sources of funding for the studies included in the review?

For Yes, must have reported on the sources of funding for individual studies included in the review. Note: Reporting that the reviewers looked for this information but it was not reported by study authors also qualifies.

11. If meta-analysis was performed did the review authors use appropriate methods for statistical combination of results?

RCTs

For Yes: the authors justified combining the data in a meta-analysis, AND they used an appropriate weighted technique to combine study results and adjusted for heterogeneity if present, AND investigated the causes of any heterogeneity.

NRSI

For Yes: the authors justified combining the data in a meta-analysis, AND they used an appropriate weighted technique to combine study results, adjusting for heterogeneity if present, AND they statistically combined effect estimates from NRSI that were adjusted for confounding, rather than combining raw data, or justified combining raw data when adjusted effect estimates were not available, AND they reported separate summary estimates for RCTs and NRSI separately when both were included in the review.

12. If meta-analysis was performed, did the review authors assess the potential impact of RoB in individual studies on the results of the meta-analysis or other evidence synthesis?

For Yes: included only low risk of bias RCTs, OR, if the pooled estimate was based on RCTs and/or NRSI at variable RoB, the authors performed analyses to investigate possible impact of RoB on summary estimates of effect.

13. Did the review authors account for RoB in individual studies when interpreting/discussing the results of the review?

For Yes: included only low risk of bias RCTs, OR, if RCTs with moderate or high RoB, or NRSI were included the review provided a discussion of the likely impact of RoB on the results.

14. Did the review authors provide a satisfactory explanation for, and discussion of, any heterogeneity observed in the results of the review?

For Yes: there was no significant heterogeneity in the results, OR if heterogeneity was present the authors performed an investigation of sources of any heterogeneity in the results and discussed the impact of this on the results of the review.

15. If they performed quantitative synthesis did the review authors carry out an adequate investigation of publication bias (small study bias) and discuss its likely impact on the results of the review?

For Yes: performed graphical or statistical tests for publication bias and discussed the likelihood and magnitude of impact of publication bias.

16. Did the review authors report any potential sources of conflict of interest, including any funding they received for conducting the review?

For Yes: the authors reported no competing interests, OR the authors described their funding sources and how they managed potential conflicts of interest.

Rating overall confidence in the results of the review

High: No or one non-critical weakness: the systematic review provides an accurate and comprehensive summary of the results of the available studies that address the question of interest.

Moderate: More than one non-critical weakness*: the systematic review has more than one weakness but no critical flaws. It may provide an accurate summary of the results of the available studies that were included in the review.

Low: One critical flaw with or without non-critical weaknesses: the review has a critical flaw and may not provide an accurate and comprehensive summary of the available studies that address the question of interest.

Critically low: More than one critical flaw with or without non-critical weaknesses: the review has more than one critical flaw and should not be relied on to provide an accurate and comprehensive summary of the available studies.

*Multiple non-critical weaknesses may diminish confidence in the review and it may be appropriate to move the overall appraisal down from moderate to low confidence.

## Supplementary Tables

### Supplementary Table S1. Keywords and search strategy in the umbrella review.

| **Database** | **Keywords** |
| --- | --- |
| MEDLINE (OvidSP) | 1. Folate  2. Folic acid  3. Vitamin B9  4. 1 or 2 or 3  5. Meta-Analysis as Topic/ or meta-analys*.mp.  4 and 5 |
| EMBASE (OvidSP) | 1. Folate  2. Folic acid  3. Vitamin B9  4. 1 or 2 or 3  5. Meta-Analysis as Topic/ or meta-analys*.mp.  4 and 5 |
| Cochrane library (OvidSP) | 1. Folate  2. Folic acid  3. Vitamin B9  4. 1 or 2 or 3  5. Meta-Analysis as Topic/ or meta-analys*.mp.  4 and 5 |

### Supplementary Table S2. General characteristics and main findings of the 133 meta-analysis of observational studies.

| **Author** | **Year** | **Outcome** | **Exposure** | **Population** | **Comparison** | **N Studies** | **N**  **Participants** | **N**  **Cases** | **Type of metric** | **Effect model reported** | **Reported summary effect (95% CI)** | **Recalculated summary effect (95% CI)** | **Concordance in overlapped MA** |
| --- | --- | --- | --- | --- | --- | --- | --- | --- | --- | --- | --- | --- | --- |
| **All cause and cause specific mortality** | | | | | | | | | | | | | |
| Li (1) | 2015 | All-cause mortality | Dietary folate intake | Breast cancer patients | Highest vs lowest | 5 | 7,299 | 1,604 | HR | Random | 0.74 (0.60, 0.92) | 0.74 (0.60, 0.92) |  |
| Li (1) | 2015 | All-cause mortality | Total folate intake | Breast cancer patients | Highest vs lowest | 4 | 4,183 | 752 | HR | Random | 0.93 (0.75, 1.15) | 0.93 (0.75, 1.15) |  |
| Li (1) | 2014 | Breast cancer mortality | Dietary folate intake | Breast cancer patients | Highest vs lowest | 2 | 4,624 | 505 | HR | Random | 0.79 (0.61, 1.01) | 0.79 (0.61, 1.01) |  |
| **Birth outcomes** | | | | | | | | | | | | | |
| Wang (2) | 2015 | Asthma | folate supplement | Any Pregnancy | Folate supplement vs. no | 3 | 9,988 | 1,377 | RR | Random | 1.04 (0.94, 1.16) | NA | Yes (2-4) |
| Wang (2) | 2015 | Asthma | folate supplement | Early pregnancy | Folate supplement vs. no | 3 | 11,865 | 656 | RR | Random | 0.98 (0.78, 1.23) | NA |  |
| Wang (2) | 2015 | Asthma | folate supplement | Pre-pregnancy | Folate supplement vs. no | 2 | 5,081 | 935 | RR | Random | 0.98 (0.73, 1.33) | NA |  |
| Wang (2) | 2015 | Asthma | folate supplement | Other period in pregnancy | Folate supplement vs. no | 2 | 7,547 | 549 | RR | Random | 1.03 (0.92, 1.16) | NA |  |
| Crider (3) | 2013 | Asthma | folate supplement | Periconceptional pregnancy | Folate supplement vs. no | 3 | 3,984 | 1,804 | RR | Random | 1.01 (0.78, 1.30) | NA |  |
| Yang (4) | 2014 | Asthma | folate supplement | Pregnancy | Folate supplement vs. no | 5 | 14,438 |  | OR | Random | 1.06 (0.99, 1.14) | 1.06 (0.99, 1.14) |  |
| Crider (3) | 2013 | Asthma or wheezing | folate supplement | Periconceptional pregnancy | Folate supplement vs. no | 5 | 44,643 |  | RR | Random | 1.05 (1.02, 1.09) | 1.05 (1.02, 1.09) |  |
| Wang (2) | 2015 | atopic dermatitis | folate supplement | Early pregnancy | Folate supplement vs. no | 2 |  |  | RR | NA | 1.15 (0.91, 1.45) | 1.15 (0.91, 1.45) |  |
| Wang (5) | 2017 | autism spectrum disorders | folate supplement | pregnancy | Folate supplement vs. no | 16 | 632,527 | 4,514 | RR | Random | 0.77 (0.64, 0.93) | 0.77 (0.64, 0.93) |  |
| Badovinac (6) | 2006 | All cleft | folate supplement | Pregnancy | Folate supplement vs. no | 12 | 577,298 | 4,876 | OR | Random | 0.77 (0.65, 0.90) | 0.77 (0.66, 0.90) |  |
| Badovinac (6) | 2006 | All cleft | folate supplement | Pregnancy | Folate supplement vs. no | 5 | 13,871 | 127 | RR | Fixed | 0.51 (0.31, 0.95) | 0.60 (0.35, 1.04) |  |
| Johnson (7) | 2008 | Cleft lip with or without cleft palate | folate supplement | Pregnancy | Folate supplement vs. no | 13 | NA | NA | OR | Random | 0.82 (0.70, 0.97) | NA | Yes (6-8) |
| Badovinac (6) | 2006 | Cleft lip with or without cleft palate | folate supplement | Pregnancy | Folate supplement vs. no | 5 | 13,525 | 110 | RR | Fixed | 0.51 (0.32, 0.95) | NA |  |
| Badovinac (6) | 2006 | Cleft lip with or without cleft palate | folate supplement | Pregnancy | Folate supplement vs. no | 12 | 573,747 | 2,914 | OR | Random | 0.77 (0.65, 0.90) | NA |  |
| Jahanbin (8) | 2018 | cleft lip with or without cleft palate | folate supplement | Pregnancy | Folate supplement vs. no | 17 | 257,840 | 4,018 | OR | Random | 0.73 (0.61, 0.85) | NA |  |
| Millacura (9) | 2017 | Cleft lip with or without cleft palate (prevalence) | folate supplement | Pregnancy | Folate supplement vs. no | 10 | 18,432,895 | 18,730 | OR | Random | 0.99 (0.92, 1.06) | 0.99 (0.92, 1.06) |  |
| Johnson (7) | 2008 | Cleft palate | folate supplement | Pregnancy | Folate supplement vs. no | 12 | NA | NA | OR | Random | 0.95 (0.79, 1.14) | NA | No (6-8) (discordance in statistical significance) |
| Badovinac (6) | 2006 | Cleft palate | folate supplement | Pregnancy | Folate supplement vs. no | 5 | 11,182 | 11 | RR | Fixed | 0.51 (0.35, 0.95) | NA |  |
| Badovinac (6) | 2006 | Cleft palate | folate supplement | Pregnancy | Folate supplement vs. no | 12 | 570,934 | 1,286 | OR | Random | 0.77 (0.65, 0.90) | NA |  |
| Jahanbin (8) | 2018 | cleft palate | folate supplement | Pregnancy | Folate supplement vs. no | 14 | 255,036 | 1,482 | OR | Random | 0.75 (0.53, 1.04) | NA |  |
| Millacura (9) | 2017 | Cleft palate (prevalence) | folate supplement | Pregnancy | Folate supplement vs. no | 10 | 18,349,548 | 10,122 | OR | Random | 1.02 (0.89, 1.18) | 1.02 (0.89, 1.18) |  |
| Feng (10) | 2015 | Congenital Heart Defects | folate supplement | Before or during pregnancy | Folate supplement vs. no | 18 | 84,651 | 18,500 | RR | Random | 0.72 (0.63, 0.82) | NA | Yes (10-12) |
| Xu (11) | 2016 | Congenital Heart Defects | folate supplement | Pregnancy | Folate supplement vs. no | 20 | 544,908 | 16,463 | OR | Random | 0.60 (0.49, 0.71) | 0.64 (0.57, 0.73) |  |
| Dean (12) | 2014 | Congenital Heart Defects | folate supplement | Pregnancy | Folate supplement vs. no | 4 | 12,861 | 199 | RR | Fixed | 0.58 (0.42, 0.79) | NA |  |
| Wang (2) | 2015 | Eczema | folate supplement | Other period in pregnancy | Folate supplement vs. no | 2 | NA | NA | RR | NA | 0.99 (0.89, 1.10) | 1.00 (0.91, 1.10) |  |
| Hodgetts (13) | 2014 | Low birthweight | folate intake | Post-conception pregnancy | Folate supplement vs. no | 2 | 43,063 | NA | aOR | NA | 0.82 (0.63, 1.06) | 0.82 (0.63, 1.06) |  |
| Hodgetts (13) | 2014 | Low birthweight | folate intake | Pre-conception pregnancy | Folate supplement vs. no | 3 | 61,096 | NA | aOR | NA | 0.75 (0.61, 0.92) | 0.75 (0.61, 0.92) |  |
| Imdad (14) | 2011 | Neural tube defects | folate supplement | pregnancy | Folate supplement vs. no | 11 | NA | NA | RR | Random | 0.59 (0.52, 0.68) | 0.59 (0.52, 0.68) |  |
| Blencowe (15) | 2010 | Neural tube defects (prevalence) | folate supplement | Women with a previous pregnancy with NTD indicates | Highest vs lowest | 8 | 3,198,772 | 3729 | RR | Random | 0.54 (0.46, 0.63) | 0.54 (0.47, 0.62) |  |
| Dean (12) | 2014 | Neural tube disorders recurrent | folate supplement | Women with a previous pregnancy with NTD indicates | Folate supplement vs. no | 2 | 1,342 | 101 | OR | NA | NA | NA |  |
| Millacura (9) | 2017 | Non-syndromic cleft lip with or without cleft palate | folate supplement | Pregnancy | Folate supplement vs. no | 3 | 4,983,797 |  | OR | Fixed | 0.88 (0.81, 0.96) | 0.88 (0.80, 0.97) |  |
| Millacura (9) | 2017 | Non-syndromic cleft palate only | folate supplement | Pregnancy | Folate supplement vs. no | 3 | 5,356,574 |  | OR | Random | 1.00 (0.75, 1.34) | 1.01 (0.75, 1.35) |  |
| Millacura (9) | 2017 | Non-syndromic orofacial clefts | folate supplement | Pregnancy | Folate supplement vs. no | 5 | 5,671,797 | 7207 | OR | Random | 0.92 (0.83, 1.03) | 0.93 (0.83, 1.03) |  |
| Millacura (9) | 2017 | Orofacial clefts | folate supplement | Pregnancy | Folate supplement vs. no | 15 | 5,9514,473 | 64,161 | OR | Random | 0.97 (0.92, 1.02) | 0.97 (0.92, 1.01) |  |
| Zhang (16) | 2016 | Preterm delivery | folate supplement | pregnancy | High dose folate supplement vs no | 2 | 37,968 | 3,463 | RR | Fixed | 0.67 (0.63, 0.72) | 0.67 (0.63, 0.72) |  |
| Zhang (16) | 2016 | Preterm delivery | folate supplement | pregnancy | Moderate to low dose folate vs no | 7 | 44,819 | 3,094 | RR | Random | 0.84 (0.74, 0.96) | 0.84 (0.74, 0.96) |  |
| Wang (2) | 2015 | Sensitization | folate supplement | Other period in pregnancy | Folate supplement vs. no | 2 | NA | NA | RR | NA | 0.88 (0.75, 1.03) | NA |  |
| Zhang (16) | 2016 | Small for gestational age | folate supplement | pregnancy | Moderate to low dose folate vs no | 6 | 71,882 | 9,837 | RR | Random | 0.76 (0.63, 0.91) | 0.76 (0.64, 0.91) |  |
| Wang (2) | 2015 | Wheeze | folate supplement | Any Pregnancy | Folate supplement vs. no | 2 | NA | NA | RR | NA | 1.05 (0.95, 1.15) | 1.05 (0.95, 1.15) |  |
| Wang (2) | 2015 | Wheeze | folate supplement | Early pregnancy | Folate supplement vs. no | 3 | NA | NA | RR | NA | 1.06 (1.02, 1.09) | 1.06 (1.02, 1.09) |  |
| Wang (2) | 2015 | Wheeze | folate supplement | Other period in pregnancy | Folate supplement vs. no | 3 | NA | NA | RR | NA | 1.01 (0.98,1.03) | 1.00 (0.96, 1.03) |  |
| **Cancer outcomes** | | | | | | | | | | | | | |
| He (17) | 2014 | Bladder cancer | total folate intake | General | Highest vs lowest | 13 | 498,372 | 6,280 | OR | Fixed | 0.84 (0.72, 0.96) | 0.88 (0.78, 1.01) |  |
| Larsson (18) | 2007 | Breast cancer | blood folate | General | Highest vs lowest | 2 | 635 | 269 | OR | NA | 0.41 (0.15, 1.10) | NA | Yes (18-20) |
| Larsson (18) | 2007 | Breast cancer | blood folate | General | Highest vs lowest | 3 | 2,683 | 837 | RR | NA | 0.81 (0.59, 1.10) | NA |  |
| Tio (19) | 2014 | Breast cancer | blood folate | General | Highest vs lowest | 7 | 5,226 | 2,403 | OR | Random | 0.86 (0.60, 1.25) | NA |  |
| Chen (20) | 2014 | Breast cancer | blood folate | General | Highest vs lowest | 4 | 1,337 | 545 | OR | NA | 0.59 (0.26, 1.31) | 0.58 (0.32, 1.05) |  |
| Chen (20) | 2014 | Breast cancer | blood folate | General | Highest vs lowest | 6 | 8,677 | 3,815 | RR | NA | 1.04 (0.76, 1.42) | 1.08 (0.87, 1.35) |  |
| Larsson (18) | 2007 | Breast cancer | dietary folate intake | General | Highest vs lowest | 13 | 19,370 | 8,558 | OR | NA | 0.73 (0.64, 0.83) | NA | No (18-21) (discordance in statistical significance) |
| Larsson (18) | 2007 | Breast cancer | dietary folate intake | General | Highest vs lowest | 8 | 303,347 | 7,067 | RR | NA | 0.96 (0.87, 1.05) | NA |  |
| Tio (19) | 2014 | Breast cancer | dietary folate intake | General | Highest vs lowest | 36 | 608,265 | 34,602 | OR | Random | 0.84 (0.77, 0.91) | NA |  |
| Chen (20) | 2014 | Breast cancer | dietary folate intake | General | Highest vs lowest | 25 | 39,363 | 17,834 | OR | Random | 0.79 (0.67, 0.92) | NA |  |
| Chen (20) | 2014 | Breast cancer | dietary folate intake | General | Highest vs lowest | 15 | 655,493 | 24,422 | RR | Random | 0.95 (0.87, 1.03) | NA |  |
| Liu (21) | 2014 | Breast cancer | dietary folate intake | General | Highest vs lowest | 15 | 1,836,566 | 24,083 | RR | Random | 0.98 (0.90, 1.05) | 0.99 (0.92, 1.06) |  |
| Lewis (22) | 2006 | Breast cancer | dietary folate intake | Postmenopausal women | 100- μ g/d increase | 4 | 4,893 | 2,259 | OR | Random | 0.92 (0.83, 1.02) | 0.92 (0.83, 1.03) |  |
| Lewis (22) | 2006 | Breast cancer | dietary folate intake | Postmenopausal women | 100- μ g/d increase | 6 | 162,033 | 7,130 | RR | Random | 1.01 (0.98, 1.05) | 1.01 (0.98, 1.05) |  |
| Lewis (22) | 2006 | Breast cancer | dietary folate intake | Premenopausa women | 100- μ g/d increase | 5 | 6,568 | 2,419 | OR | Random | 0.87 (0.78, 0.97) | 0.87 (0.78, 0.97) |  |
| Lewis (22) | 2006 | Breast cancer | dietary folate intake | Premenopausa women | 100- μ g/d increase | 3 | 15,5926 | 1,828 | RR | Random | 1.01 (0.98, 1.04) | 1.00 (0.97, 1.04) |  |
| Chen (20) | 2014 | Breast cancer | folate supplement | General | Highest vs lowest | 3 | 86,647 | 2,506 | RR | NA | 1.07 (0.95, 1.21) | 1.07 (0.95, 1.21) |  |
| Zhang (23) | 2014 | Breast cancer | total folate intake | General | Highest vs lowest | 14 | 677,858 | 18,705 | RR | Random | 0.97 (0.90, 1.05) | NA | Yes (19, 20, 23) |
| Tio (19) | 2014 | Breast cancer | total folate intake | General | Highest vs lowest | 15 | 543,650 | 22,134 | OR | NA | 0.98 (0.91, 1.07) | 0.98 (0.90, 1.07) |  |
| Chen (20) | 2014 | Breast cancer | total folate intake | General | Highest vs lowest | 11 | 476,699 | 16,717 | RR | Random | 0.97 (0.87, 1.08) | NA |  |
| Zhou (24) | 2016 | Cervical cancer | serum folate | General | Deficient vs. normal | 6 | 2,383 | 873 | OR | Random | 1.94 (1.14, 3.31) | 1.91 (1.14, 3.30) |  |
| Myung (25) | 2011 | Cervical neoplasm | folate intake and serum folate | General | Folate supplement vs. no | 9 | 3,089 | 1,706 | OR | NA | 0.60 (0.41, 0.88) | 0.60 (0.41, 0.88) |  |
| Kim (26) | 2010 | Colon cancer | dietary folate intake | General | Highest vs lowest | 13 | 725,134 | 5,720 | RR | Random | 0.92 (0.84, 1.00) | NA |  |
| Kim (26) | 2010 | Colon cancer | folate intake and circulating folate | General | Highest vs lowest | 8 | 725,134 | 5,720 | RR | Random | 0.85 (0.77, 0.95) | NA |  |
| Park (27) | 2017 | Colorectal adenoma | Circulating folate | General | Lowest vs highest | 11 | 17,831 | 4,812 | RR | Fixed | 1.15 (1.03, 1.27) | 1.23 (1.09, 1.39) |  |
| Chuang (28) | 2013 | Colorectal cancer | Circulating folate | General | Highest vs lowest | 8 | 10,516 | 34,77 | RR | Fixed | 0.91 (0.77, 1.05) | 1.01 (0.87, 1.17) |  |
| Sanjoaquin (29) | 2004 | Colorectal cancer | dietary folate intake | General | Highest vs lowest | 7 | 9,676 | 6,166 | OR | NA | 0.76 (0.60, 0.96) | NA | No (29, 30) (discordance in statistical significance) |
| Sanjoaquin (29) | 2004 | Colorectal cancer | dietary folate intake | General | Highest vs lowest | 5 | 2,394 | NA | RR | NA | 0.75 (0.64, 0.89) | NA |  |
| Kennedy (30) | 2010 | Colorectal cancer | dietary folate intake | General | Highest vs lowest | 13 | 18,992 | 8,328 | OR | Random | 0.87 (0.74, 1.02) | 0.87 (0.74, 1.02) |  |
| Kennedy (30) | 2010 | Colorectal cancer | dietary folate intake | General | Highest vs lowest | 9 | 471,924 | 6,633 | HR | Random | 0.92 (0.81, 1.05) | 0.92 (0.81, 1.05) |  |
| Heine-Br€oring (31) | 2015 | Colorectal cancer | folate supplement | General | Highest vs lowest | 3 | 169,360 | 3,783 | RR | NA | 0.88 (0.78, 0.98) | 0.87 (0.74, 1.01) | No (31, 32) (discordance in statistical significance) |
| Moazzen (32) | 2017 | Colorectal cancer | folate supplement | General | Folate supplement vs. no | NA | NA | NA | RR | Random | 0.96 (0.76, 1.21) | NA |  |
| Burr (33) | 2013 | Colorectal cancer | folate supplement | Patients with Inflammatory Bowel Disease | Folate supplement vs. no | 10 | 4,517 | 622 | OR | Random | 0.58 (0.37, 0.80) | 0.71 (0.53, 0.96) |  |
| Moazzen (32) | 2017 | Colorectal cancer | RBC folate | General | Folate supplement vs. no | 8 | 7,908 | 3,008 | RR | Random | 1.05 (0.85, 1.30) | 1.04 (0.84, 1.29) |  |
| Sanjoaquin (29) | 2004 | Colorectal cancer | Total folate intake | General | Highest vs lowest | 3 | 1,499 | 968 | OR | NA | 0.81 (0.62, 1.05) | NA | No (29, 30, 32, 34) (discordance in statistical significance) |
| Sanjoaquin (29) | 2004 | Colorectal cancer | total folate intake | General | Highest vs lowest | 3 | 2,689 | NA | RR | NA | 0.95 (0.81, 1.11) | NA |  |
| Kennedy (30) | 2010 | Colorectal cancer | total folate intake | General | Highest vs lowest | 8 | 1,679 | 725 | OR | Random | 0.85 (0.74, 0.99) | NA |  |
| Moazzen (32) | 2017 | Colorectal cancer | total folate intake | General | Highest vs lowest | NA | NA | NA | OR | Random | 0.77 (0.62, 0.95) | NA |  |
| Moazzen (32) | 2017 | Colorectal cancer | total folate intake | General | Highest vs lowest | NA | NA | NA | RR | Random | 0.71 (0.59, 0.86) | NA |  |
| Liu (34) | 2015 | Colorectal cancer | total folate intake | General | Highest vs lowest | 23 | 1,896,788 | 23,147 | RR | Random | 0.88 (0.81, 0.95) | 0.88 (0.81, 0.95) |  |
| Du (35) | 2016 | Endometrial cancer | total folate intake | General | Highest vs lowest | 14 | 270,542 | 6,151 | OR | Random | 0.89 (0.76, 1.05) | 0.89 (0.76, 1.05) |  |
| LARSSON (36) | 2006 | Esophageal adenocarcinoma | dietary folate intake | General | Highest vs lowest | 7 | 1,769 | 501 | RR | NA | 0.50 (0.39, 0.65) | 0.50 (0.49, 0.65) |  |
| Zhao (37) | 2017 | Esophageal cancer | serum folate | General | Highest vs lowest | 4 | 36,243 | 5,489 | OR | Random | 0.71 (0.55, 0.92) | 0.70 (0.31, 1.59) | No (37, 38) (discordance in statistical significance) |
| Ni (38) | 2018 | Esophageal cancer | serum folate | General | Highest vs lowest | 5 | 1,344 | 700 | OR | Random | 0.45 (0.19, 1.07) | NA |  |
| Tio (39) | 2013 | Esophageal cancer | dietary folate intake | General | Highest vs lowest | 9 | 9,254 | 2,574 | OR | Random | 0.59 (0.51, 0.69) | NA | Yes (37-40) |
| Zhao (37) | 2017 | Esophageal cancer | dietary folate intake | General | Highest vs lowest | 15 | 4,480,404 | 2,036 | OR | Random | 0.63 (0.56, 0.71) | 0.66 (0.52, 0.83) |  |
| Liu (40) | 2011 | Esophageal cancer | dietary folate intake | General | Highest vs lowest | 6 | 9,495 | 1,817 | OR | Random | 0.60 (0.50, 0.70) | NA |  |
| Ni (38) | 2018 | Esophageal cancer | dietary folate intake | General | Highest vs lowest | 15 | NA | 3,793 | OR | Random | 0.64 (0.54, 0.76) | NA |  |
| Liu (41) | 2017 | Esophageal cancer | folate intake and serum folate | General | Highest vs lowest | 19 | 557,646 | 5,442 | OR | Random | 0.55 (0.43, 0.66) | 0.59 (0.49, 0.71) |  |
| Tio (39) | 2013 | Gastric cancer | dietary folate intake | General | Highest vs lowest | 16 | 209,689 | 4,414 | OR | NA | 0.98 (0.81, 1.19) | 0.94 (0.78, 1.13) | Yes (36, 39) |
| LARSSON (36) | 2006 | Gastric cancer | dietary folate intake | General | Highest vs lowest | 11 | 3,205 | 73,344 | RR | Random | 0.90 (0.72, 1.13) | NA |  |
| Liu (41) | 2017 | Gastric cancer | folate intake and serum folate | General | Highest vs lowest | 21 | 857,918 | 6,810 | OR | Random | 0.76 (0.65, 0.88) | 0.82 (0.71, 0.94) |  |
| Fan (42) | 2017 | Head and neck squamous cell carcinoma | folate intake and serum folate | General | Highest vs lowest | 9 | 14,992 | 4,090 | OR | Random | 0.50 (0.39, 0.62) | 0.53 (0.41, 0.68) |  |
| DAI (43) | 2013 | Lung Cancer | dietary folate intake | General | Highest vs lowest | 6 | 9,275 | 3,805 | OR | Fixed | 0.73 (0.63, 0.85) | NA | No (43, 44) (discordance in statistical significance) |
| Zhang (44) | 2014 | Lung Cancer | dietary folate intake | General | Highest vs lowest | 9 | 508,767 | 4,681 | RR | Fixed | 0.92 (0.84, 1.01) | 0.92 (0.84, 1.01) |  |
| DAI (43) | 2013 | Lung Cancer | serum folate | General | Highest vs lowest | 5 | 6,008 | 1,482 | OR | Random | 0.78 (0.60, 1.02) | 0.78 (0.60, 1.03) |  |
| DAI (43) | 2013 | Lung Cancer | total folate intake | General | Highest vs lowest | 6 | 10,528 | 4,390 | OR | Fixed | 0.74 (0.65, 0.84) | NA |  |
| Li (45) | 2013 | Ovarian cancer | dietary folate intake | General | Highest vs lowest | 8 | 227,859 | 5,677 | RR | Random | 0.88 (0.75, 1.05) | 0.88 (0.75, 1.05) |  |
| Lin (46) | 2013 | Pancreatic cancer | blood folate | General | Highest vs lowest | 3 | 1,753 | 826 | OR | Random | 0.80 (0.44, 1.45) | 0.80 (0.45, 1.45) | Yes (39, 46) |
| Tio (39) | 2013 | Pancreatic cancer | Blood folate | General | Highest vs lowest | 4 | 2,215 | 839 | OR | Random | 0.73 (0.47, 1.13) | NA |  |
| Tio (39) | 2013 | Pancreatic cancer | dietary folate intake | General | Highest vs lowest | 8 | 295,776 | 2,459 | OR | NA | 0.66 (0.49, 0.89) | 0.66 (0.49, 0.89) | Yes (36, 39, 46) |
| LARSSON (36) | 2006 | Pancreatic cancer | dietary folate intake | General | Highest vs lowest | 5 | 236,788 | 722 | RR | Random | 0.49 (0.35 ,0.67) | NA |  |
| Lin (46) | 2013 | Pancreatic cancer | dietary folate intake | General | Highest vs lowest | 9 | 1,330,762 | 4,404 | RR | Random | 0.66 (0.49, 0.88) | NA |  |
| Liu (41) | 2017 | Pancreatic cancer | folate intake and serum folate | General | Highest vs lowest | 12 | 997,922 | 3,067 | OR | Random | 0.73 (0.55, 0.91) | 0.76 (0.60, 0.96) |  |
| Wien (47) | 2011 | Pancreatic cancer | folate supplement | General | Folate supplement vs. no | 2 | NA | NA | RR | NA | 1.12 (0.90, 1.40) | NA | Yes (46, 47) |
| Lin (46) | 2013 | Pancreatic cancer | folate supplement | General | Folate supplement vs. no | 4 | 235,389 | 1,175 | OR | Random | 1.08 (0.82, 1.41) | 1.08 (0.82, 1.41) |  |
| Tio (39) | 2013 | Pancreatic cancer | total folate intake | General | Highest vs lowest | 4 | 261,727 | 1,259 | RR | Random | 0.69 (0.47, 1.03) | NA | Yes (39, 46) |
| Lin (46) | 2013 | Pancreatic cancer | total folate intake | General | Highest vs lowest | 3 | 209,635 | 993 | OR | Random | 0.75 (0.50, 1.13) | NA |  |
| Wang (48) | 2014 | Prostate cancer | Serum folate | General | Highest vs lowest | 5 | 36,243 | 5,489 | RR | Random | 1.21 (1.05, 1.39) | 1.21 (1.05, 1.39) |  |
| Tio (49) | 2014 | Prostate cancer | blood folate | General | Highest vs lowest | 7 | 13,232 | 6,122 | OR | NA | 1.43 (1.06, 1.93) | 1.43 (1.06, 1.93) |  |
| Price (50) | 2016 | Prostate cancer | Circulating Folate | General | Highest vs lowest | 6 | 11,730 | 5,277 | OR | NA | 1.17 (1.05, 1.31) | NA | No (50, 51) (discordance in statistical significance) |
| Collin (51) | 2010 | Prostate cancer | Circulating Folate | General | 10mmol/L increase | 7 | 9,353 | 2,958 | OR | Random | 1.11 (0.96, 1.28) | 1.11 (0.96, 1.28) |  |
| Tio (49) | 2014 | Prostate cancer | dietary folate intake | General | Highest vs lowest | 11 | 120,349 | 14,290 | OR | NA | 0.97 (0.89, 1.06) | 0.98 (0.90, 1.07) | Yes (48, 49) |
| Wang (48) | 2014 | Prostate cancer | dietary folate intake | General | Highest vs lowest | 5 | 192,702 | 12,306 | RR | Random | 1.02 (0.95, 1.09) | NA |  |
| Tio (49) | 2014 | Prostate cancer | total folate intake | General | Highest vs lowest | 5 | 93,781 | 7,114 | OR | NA | 0.99 (0.82, 1.19) | NA |  |
| Mao (52) | 2015 | Renal Cell Cancer | folate supplement | General | folate supplement vs no | 8 | 374,901 | 2,723 | RR | NA | 0.87 (0.72, 1.05) | 0.89 (0.77, 1.02) |  |
| Milne (53) | 2009 | Acute lymphoblastic leukemia | folate supplement | 1 month before pregnancy | Folate supplement vs. no | 2 | 2,042 | 490 | OR | NA | 1.06 (0.77, 1.46) | 1.06 (0.77, 1.46) |  |
| **Cardiovascular outcomes** | | | | | | | | | | | | | |
| Wang(54) | 2011 | Coronary heart disease | blood folate | General | Highest vs lowest | 8 | 14533 | 1936 | RR | Random | 0.74 (0.53, 1.02) | 0.74 (0.53, 1.02) |  |
| Wang (54) | 2011 | Coronary heart disease | dietary folate intake | General | Highest vs lowest | 7 | 221,009 | 2,682 | RR | Random | 0.69 (0.60, 0.80) | 0.69 (0.60, 0.80) |  |
| **Neurocognitive disorders** | | | | | | | | | | | | | |
| Shen (55) | 2015 | Alzheimer's disease | serum folate | General | Lower vs higher | 6 | 2,070 | 476 | RR | Fixed | 2.11 (1.51, 2.71) | 2.22 (1.71, 2.89) |  |
| Michelakos (56) | 2013 | Cognitive impairment | serum folate | Seniors | Lower vs higher | 13 | 10,104 |  | OR | Random | 1.66 (1.40, 1.96) | 1.66 (1.40, 1.96) |  |
| Gilbody (57) | 2007 | Depression | circulating folate | General | Highest vs lowest | 10 | 15,215 | 1,769 | OR | NA | 1.55 (1.26, 1.91) | 1.58 (1.17, 2.14) |  |
| Petridou (58) | 2015 | Depression | serum folate | General | Highest vs lowest | 11 | 7,949 | 1,783 | OR | Fixed | 1.23 (1.07, 1.43) | 1.22 (1.02, 1.46) |  |
| Shen (59) | 2015 | Parkinson's disease | dietary folate intake | General | Highest vs lowest | 3 | 143,643 | 794 | OR | Fixed | 1.01 (0.68, 1.34) | 1.06 (0.78, 1.45) |  |
| **Pregnancy outcomes** | | | | | | | | | | | | | |
| Yang (60) | 2016 | Gestational hypertension | folate supplement | Pregnancy | Folate supplement vs. no | 4 | 1202706 | 77,859 | RR | Random | 1.03 (0.98, 1.09) | 1.03 (0.98, 1.09) |  |
| Hua (61) | 2016 | Gestational hypertension/preeclampsia | folate supplement | Pregnancy | Folate supplement vs. no | 9 | 279286 | 147,424 | RR | NA | 0.92 (0.79, 1.08) | 0.92 (0.79, 1.09) |  |
| Yang (60) | 2016 | Preeclampsia | folate supplement | Pregnancy | Folate supplement vs. no | 7 | 1242398 | 43,818 | ES | Random | 0.99 (0.90, 1.08) | 0.99 (0.90, 1.08) | Yes (60, 61) |
| Hua (61) | 2012 | Preeclampsia | folate supplement | Pregnancy | Folate supplement vs. no | 8 | 279,286 | 6,789 | RR | Random | 0.88 (0.76, 1.02) | NA |  |
| **Other outcomes** | | | | | | | | | | | | | |
| Bailie (62) | 2017 | Serrated Colorectal Polyps | dietary folate intake | General | Highest vs lowest | 3 | 32,462 | 831 | RR | Random | 0.65 (0.49, 0.85) | 0.65 (0.50, 0.85) |  |

### Supplementary Table S3. Unique health outcomes reported in meta-analysis of observational studies.

| **Outcomes** | **Exposure** | **Population** | **Study design included in MA** | **Comparison** | **N studies** | **N participants** | **N cases** | **Type of metric** | **Relative Risk (95% CI)** | ***P* value** | ***I^2^***  **(95% CI)** | ***P* value for heterogeneity** | ***P* value for Egger test** | ***P* value for excess significance test** | **95% prediction interval** |
| --- | --- | --- | --- | --- | --- | --- | --- | --- | --- | --- | --- | --- | --- | --- | --- |
| **All cause and cause specific mortality** | | | | | | | | | | | | | | | |
| All-cause mortality (1) | Dietary folate intake | Breast cancer patients | Prospective cohort | Highest vs lowest | 5 | 7,299 | 1,604 | HR | 0.74 (0.60, 0.92) | 0.006 | 36 (0-76) | 0.183 | 0.401 | NP | 0.42-1.31 |
| All-cause mortality (1) | Total folate intake | Breast cancer patients | Prospective cohort | Highest vs lowest | 4 | 4,183 | 752 | HR | 0.93 (0.75, 1.15) | 0.503 | 0 (0-68) | 0.956 | 0.708 | NP | 0.59-1.48 |
| Breast cancer mortality (1) | Dietary folate intake | Breast cancer patients | Prospective cohort | Highest vs lowest | 2 | 4,624 | 505 | HR | 0.79 (0.61, 1.01) | 0.064 | NA | NA | NA | NP | NA |
| **Birth outcomes** | | | | | | | | | | | | | | | |
| Asthma (60) | folate supplement | Pregnancy | cohort | Folate supplement vs. no | 5 | 14,438 | NA | OR | 1.06 (0.99, 1.14) | 0.105 | 0 (0-64) | 0.444 | 0.685 | NP | 0.94-1.20 |
| asthma or wheezing (3) | folate supplement | Pregnancy with periconceptional period | cohort studies | Folate supplement vs. no | 5 | 44,643 | NA | RR | 1.05 (1.02, 1.09) | 0.001 | 0 (0-64) | 0.700 | 0.525 | NP | 1.00-1.11 |
| atopic dermatitiss (2) | folate supplement | Early pregnancy | cohort | Folate supplement vs. no | 2 | NA | NA | RR | 1.15 (0.91, 1.45) | 0.233 | NA | NA | NA | NP | NA |
| autism spectrum disorders (5) | folate supplement | pregnancy | cohort and case-control | Folate supplement vs. no | 16 | 632,527 | 4,514 | RR | 0.77 (0.64, 0.93) | 0.006 | 59 (18-75) | 0.001 | 0.005 | 0.154 | 0.46-1.31 |
| All cleft (6) | folate supplement | Pregnancy | case-control | Folate supplement vs. no | 12 | 577,298 | 4,876 | OR | 0.77 (0.66, 0.90) | 0.001 | 67 (28-80) | 0.001 | 0.685 | NP | 0.46-1.30 |
| All cleft (6) | folate supplement | Pregnancy | cohort | Folate supplement vs. no | 5 | 13,871 | 127 | RR | 0.60 (0.35, 1.04) | 0.070 | 0 (0-64) | 0.550 | 0.669 | NP | 0.25-1.47 |
| Cleft lip with or without cleft palate (8) | folate supplement | pregnancy | case control and cohort | Highest vs lowest | 17 | 257,840 | 4,018 | OR | 0.72 (0.61, 0.85) | 7.75E-05 | 63 (30-77) | <0.001 | 0.663 | 0.552 | NP |
| Cleft lip with or without cleft palate (Prevalence) (9) | folate supplement | Pregnancy | Case-control | Folate supplement vs. no | 10 | 18,432,895 | 18,730 | OR | 0.99 (0.92, 1.06) | 0.702 | 76 (59-84) | <0.001 | 0.254 | 0.774 | 0.75-1.29 |
| Cleft palate (8) | folate supplement | pregnancy | case control and cohort | Folate supplement vs. no | 14 | 255,036 | 1,482 | OR | 0.74 (0.54, 1.03) | 0.077 | 81 (67-87) | <0.001 | 0.180 | 0.000352 | 0.22-2.47 |
| Cleft palate (prevalence) (9) | folate supplement | Pregnancy | Case-control | Folate supplement vs. no | 10 | 18,349,548 | 10,122 | OR | 1.02 (0.89, 1.18) | 0.744 | 89 (84-92) | <0.001 | 0.388 | 0.086 | 0.58-1.82 |
| Congenital Heart Defects (11) | folate supplement | General | case-control + cohort | Folate supplement vs. no | 20 | 544,908 | 16,463 | OR | 0.64 (0.57, 0.73) | 3.01E-11 | 80 (70-86) | <0.001 | < 0.001 | 1.00E-08 | 0.41-1.02 |
| Eczema (2) | folate supplement | Other period in pregnancy | cohort | Folate supplement vs. no | 2 | NA | NA | RR | 1.00 (0.91, 1.10) | 0.991 | NA | NA | NA | NP | NA |
| Low birthweight (13) | folate intake | Post-conception pregnancy | cohort | Folate supplement vs. no | 2 | 43,063 | NA | aOR | 0.82 (0.63, 1.06) | 0.132 | NA | NA | NA | 0.943 | NA |
| Low birthweight (13) | folate intake | Pre-conception pregnancy | cohort | Folate supplement vs. no | 3 | 61,096 | NA | aOR | 0.75 (0.61, 0.92) | 0.006 | 64 (0-88) | 0.060 | 0.042 | NP | 0.08-6.68 |
| Neural tube defects (14) | folate supplement | pregnancy | before and after studies | Folate supplement vs. no | 11 | NA | NA | RR | 0.59 (0.52, 0.68) | 3.33E-14 | 88 (80-92) | <0.001 | 0.088 | NP | 0.36-0.96 |
| Neural tube defects (prevalence) (15) | folate supplement | Women with a previous pregnancy with NTD indicates | Case-control | Highest vs lowest | 8 | 3,198,772 | 3729 | RR | 0.54 (0.47, 0.62) | 1.15E-16 | 67 (6-82) | 0.004 | 0.113 | NP | 0.35-0.83 |
| Neural tube disorders recurrent (12) | folate supplement | Pregnancy | Case and cohort | Folate supplement vs. no | 2 | 1,342 | 101 | OR | 0.63 (0.15, 2.64) | 0.532 | 88 (NA) | <0.001 | 0.112 | 0.070 | NA |
| Non-syndromic cleft lip with or without cleft palate (9) | folate supplement | Pregnancy | Case-control | Folate supplement vs. no | 3 | 4,983,797 | NA | OR | 0.88 (0.80, 0.97) | 0.009 | 22 (0-71) | 0.276 | 0.800 | NP | 0.71-1.10 |
| Non-syndromic cleft palate only (9) | folate supplement | Pregnancy | Case-control | Folate supplement vs. no | 3 | 5,356,574 | NA | OR | 1.01 (0.75, 1.35) | 0.971 | 69 (0-86) | 0.011 | 0.904 | NP | 0.38-2.67 |
| Non-syndromic orofacial clefts(9) | folate supplement | Pregnancy | Case-control | Folate supplement vs. no | 5 | 5671797 | 7207 | OR | 0.93(0.83,1.03) | 0.166 | 53(0-78) | 0.047 | 0.534 | NP | 0.69-1.25 |
| Orofacial clefts(9) | folate supplement | Pregnancy | Case-control | Folate supplement vs. no | 15 | 59514473 | 64161 | OR | 0.97(0.92,1.01) | 0.137 | 71(54-80) | <0.001 | 0.608 | 0.006 | 0.82-1.14 |
| Preterm delivery(16) | folate supplement | pregnancy | cohort | High dose folate supplement vs no | 2 | 37968 | 3463 | RR | 0.67(0.63,0.72) | 5.87E-33 | NA | NA | NA | 0.221 | NA |
| Preterm delivery(16) | folate supplement | pregnancy | cohort | Moderate to low dose folate vs no | 7 | 44819 | 3094 | RR | 0.84(0.74,0.96) | 0.010 | 44(0-75) | 0.096 | 0.169 | 0.561 | 0.60-1.17 |
| Sensitization(2) | folate supplement | Other period in pregnancy | cohort | Folate supplement vs. no | 2 | NA | NA | RR | NA | NA | NA | NA | NA | NA | NA |
| Small for gestational age(16) | folate supplement | pregnancy | cohort | Moderate to low dose folate vs no | 6 | 71882 | 9837 | RR | 0.76(0.64,0.91) | 0.003 | 86(74-91) | <0.001 | 0.926 | 0.975 | 0.45-1.30 |
| Wheeze(2) | folate supplement | Any Pregnancy | cohort | Folate supplement vs. no | 2 | NA | NA | RR | 1.05(0.95,1.15) | 0.363 | 0 | 0.578 | NA | NP | NA |
| Wheeze (2) | folate supplement | Early pregnancy | cohort | Folate supplement vs. no | 3 | NA | NA | RR | 1.06 (1.02, 1.09) | 0.001 | 0 | 0.761 | 0.112 | NP | NA |
| Wheeze (2) | folate supplement | Other period in pregnancy | cohort | Folate supplement vs. no | 3 | NA | NA | RR | 1.00 (0.96, 1.03) | 0.762 | 0 (0-73) | 0.587 | 0.486 | NP | 0.83-1.21 |
| **Cancer outcomes** | | | | | | | | | | | | | | | |
| bladder cancer (17) | total folate intake | General | case-control + cohort | Highest vs lowest | 13 | 498,372 | 6,280 | OR | 0.88 (0.78, 1.01) | 0.067 | 26 (0-61) | 0.184 | 0.185 | 0.373 | 0.65-1.20 |
| breast cancer (20) | blood folate | General | case-control | Highest vs lowest | 4 | 1,337 | 545 | OR | 0.58 (0.32, 1.05) | 0.072 | 69 (0-87) | 0.023 | 0.017 | NP | 0.05-7.06 |
| breast cancer (20) | blood folate | General | cohort | Highest vs lowest | 6 | 8,677 | 3,815 | RR | 1.08 (0.87, 1.35) | 0.483 | 57 (0-78) | 0.016 | 0.222 | NP | 0.59-1.98 |
| breast cancer (21) | dietary folate intake | General | cohort | Highest vs lowest | 15 | 1,836,566 | 24,083 | RR | 0.99 (0.92, 1.06) | 0.756 | 49 (0-74) | 0.018 | 0.585 | NP | 0.79-1.23 |
| breast cancer (22) | dietary folate intake | Postmenopausal women | case-control | 100- μ g/d increase | 4 | 4,893 | 2,259 | OR | 0.92 (0.83, 1.03) | 0.136 | 57 (0-84) | 0.074 | 0.510 | 0.202 | 0.61-1.40 |
| breast cancer (22) | dietary folate intake | Postmenopausal women | cohort | 100- μ g/d increase | 6 | 162,033 | 7,130 | RR | 1.01 (0.98, 1.05) | 0.473 | 27 (0-71) | 0.231 | 0.850 | 0.293 | 0.93-1.10 |
| breast cancer (22) | dietary folate intake | Premenopausal women | case-control | 100- μ g/d increase | 5 | 6,568 | 2,419 | OR | 0.87 (0.78, 0.97) | 0.011 | 73 (0-87) | 0.005 | 0.411 | 0.186 | 0.60-1.25 |
| breast cancer (22) | dietary folate intake | Premenopausal women | cohort | 100- μ g/d increase | 3 | 155,926 | 1,828 | RR | 1.00 (0.97, 1.04) | 0.782 | 0 (0-73) | 0.584 | 0.133 | NP | 0.81-1.24 |
| breast cancer (20) | folate supplement | General | cohort | Highest vs lowest | 3 | 86,647 | 2,506 | RR | 1.07 (0.95, 1.21) | 0.285 | 22 (0-78) | 0.279 | 0.613 | 0.005 | 0.38-2.98 |
| breast cancer (23) | total folate intake | General | case-control and cohort | Highest vs lowest | 15 | 543,650 | 22,134 | OR | 0.98 (0.90, 1.07) | 0.707 | 52 (0-72) | 0.009 | 0.414 | 0.108 | 0.76-1.27 |
| cervical cancer (24) | serum folate | General | case-control | Deficient vs. normal | 6 | 2,383 | 873 | OR | 1.91 (1.14, 3.30) | 0.015 | 81 (49-89) | <0.001 | 0.003 | 6.20E-07 | 0.33-11.30 |
| cervical neoplasm (25) | folate intake and serum folate | General | case–control | Folate supplement vs. no | 9 | 3,089 | 1,706 | OR | 0.60 (0.41, 0.88) | 0.008 | 60 (0-79) | 0.011 | 0.085 | NP | 0.19-1.83 |
| colon cancer (26) | dietary folate intake | General | prospective study | Highest vs lowest | 13 | 725,134 | 5,720 | RR | NA | NA | NA | NA | NA | NA | NA |
| colon cancer (26) | folate intake and ciuculating folate | General | prospective study | Highest vs lowest | 8 | 725,134 | 5,720 | RR | NA | NA | NA | NA | NA | NA | NA |
| Colorectal adenoma (27) | Circulating folate | General | case-control + cross sectional + cohort + clinical trials | Lowest vs highest | 11 | 17,831 | 4,812 | RR | 1.23 (1.09, 1.39) | 0.001 | 55 (0-74) | 0.007 | 0.019 | 0.001 | 0.86-1.76 |
| Colorectal cancer (28) | circulating folate | General | cohort | Highest vs lowest | 8 | 10,516 | 3,477 | RR | 1.01 (0.87, 1.17) | 0.888 | 11 (0-56) | 0.341 | 0.622 | 0.875 | 0.79-1.30 |
| Colorectal cancer (30) | dietary folate intake | General | case-control | Highest vs lowest | 13 | 18,992 | 8,328 | OR | 0.87 (0.74, 1.02) | 0.095 | 63 (31-77) | <0.001 | 0.063 | NP | 0.50-1.52 |
| Colorectal cancer (30) | dietary folate intake | General | cohort | Highest vs lowest | 9 | 471,924 | 6,633 | HR | 0.92 (0.81, 1.05) | 0.207 | 42 (0-70) | 0.066 | 0.808 | 0.641 | 0.65-1.30 |
| Colorectal cancer (31) | folate supplement | General | prospective cohort | Highest vs lowest | 3 | 169,360 | 3,783 | RR | 0.87 (0.74, 1.01) | 0.076 | 23 (0-75) | 0.272 | 0.191 | 0.018 | 0.54-1.40 |
| Colorectal cancer (33) | folate supplement | Patients with Inflammatory Bowel Disease | case-control +cohort | Folate supplement vs. no | 10 | 4,517 | 638 | OR | 0.71 (0.53, 0.96) | 0.026 | 25 (0-64) | 0.210 | 0.843 | 0.527 | 0.37-1.36 |
| Colorectal cancer (32) | RBC folate | General | case-control | Folate supplement vs. no | 8 | 7,908 | 3,008 | RR | 1.04 (0.84, 1.29) | 0.700 | 44 (0-72) | 0.065 | 0.657 | 0.346 | 0.59-1.84 |
| colorectal cancer (34) | total folate intake | General | cohort studies | Highest vs lowest | 23 | 1,896,788 | 23,147 | RR | 0.88 (0.81, 0.95) | 0.002 | 43 (0-64) | 0.016 | 0.082 | NP | 0.68-1.15 |
| endometrial cancer (35) | total folate intake | General | case-control and cohort | Highest vs lowest | 14 | 270,542 | 6,151 | OR | 0.89 (0.76, 1.05) | 0.169 | 59 (11-76) | 0.003 | 0.791 | NP | 0.52-1.52 |
| esophageal adenocarcinoma (36) | dietary folate intake | General | case-control | Highest vs lowest | 3 | 1,769 | 501 | RR | 0.50 (0.49, 0.65) | 1.48E-07 | 0 (0-73) | 0.737 | 0.325 | NP | 0.09-2.68 |
| esophageal cancer (37) | serum folate | General | case-control and cohort | Highest vs lowest | 4 | 36,243 | 5,489 | OR | 0.70 (0.31, 1.59) | 0.392 | 88 (73-93) | <0.001 | 0.961 | 3.99E-04 | 0.03-15.04 |
| esophageal cancer (37) | dietary folate intake | General | case-control and cohort | Highest vs lowest | 15 | 4,480,404 | 2,036 | OR | 0.66 (0.52, 0.83) | 3.46E-04 | 70 (47-80) | <0.001 | 0.580 | NP | 0.27-1.58 |
| esophageal cancer (41) | folate intake and serum folate | General | case-control + cohort | Highest vs lowest | 19 | 557,646 | 5,442 | OR | 0.59 (0.49, 0.71) | 1.83E-08 | 68 (48-78) | <0.001 | 0.023 | 0.600 | 0.28-1.26 |
| gastric cancer (39) | dietary folate intake | General | cohort | Highest vs lowest | 16 | 209,689 | 4,414 | OR | 0.94 (0.78, 1.13) | 0.509 | 55 (13-72) | 0.002 | 0.315 | NP | 0.49-1.78 |
| gastric cancer r (41) | folate intake and serum folate | General | case-control + cohort | Highest vs lowest | 21 | 857,918 | 6,810 | OR | 0.82 (0.71, 0.94) | 0.005 | 68 (48-78) | <0.001 | 0.478 | NP | 0.46-1.44 |
| Head and neck squamous cell carcinoma (42) | folate intake and serum folate | General | case-control | Highest vs lowest | 9 | 14,992 | 4,090 | OR | 0.53 (0.41, 0.68) | 7.87E-07 | 62 (0-80) | 0.008 | 0.278 | NP | 0.24-1.14 |
| Lung Cancer (44) | dietary folate intake | General | cohort | Highest vs lowest | 9 | 508,767 | 4,681 | RR | 0.92 (0.84, 1.01) | 0.076 | 0 (0-53) | 0.495 | 0.959 | NP | 0.82-1.03 |
| Lung Cancer (43) | serum folate | General | case-control + cohort | Highest vs lowest | 5 | 6,008 | 1,482 | OR | 0.78 (0.60, 1.03) | 0.078 | 0 (0-64) | 0.592 | 0.078 | NP | 0.50-1.22 |
| Lung Cancer (43) | total folate intake | General | case-control | Highest vs lowest | 6 | 10,528 | 4,390 | OR | 0.74 (0.65, 0.84) | 4.48E-06 | 0 (0-61) | 0.631 | 0.250 | NP | 0.62-0.89 |
| ovarian cancer (45) | dietary folate intake | General | Case-control+cohort | Highest vs lowest | 8 | 227,859 | 5,677 | RR | 0.88 (0.75, 1.05) | 0.154 | 40 (0-73) | 0.093 | 0.727 | NP | 0.58-1.35 |
| pancreatic cancer (46) | blood folate | General | Cohort | Highest vs lowest | 3 | 1,753 | 826 | OR | 0.80 (0.45, 1.45) | 0.463 | 71 (0-89) | 0.032 | 0.489 | 0.991 | 0.00-684.77 |
| pancreatic cancer (39) | dietary folate intake | General | Case-control + cohort | Highest vs lowest | 8 | 295,776 | 2,459 | OR | 0.66 (0.49, 0.89) | 0.006 | 71 (23-84) | 0.001 | 0.193 | 0.427 | 0.27-1.61 |
| pancreatic cancer (41) | folate intake and serum folate | General | Case-control + cohort | Highest vs lowest | 12 | 997,922 | 3,067 | OR | 0.76 (0.60, 0.96) | 0.023 | 68 (33-81) | <0.001 | 0.089 | NP | 0.34-1.69 |
| pancreatic cancer (46) | folate supplement | General | case-control + cohort | Highest vs lowest | 4 | 235,389 | 1,175 | OR | 1.08 (0.82, 1.41) | 0.596 | 14 (0-69) | 0.328 | 0.392 | NP | 0.61-1.89 |
| prostate cancer (49) | blood folate | General | case-control and cohort | Highest vs lowest | 7 | 13,232 | 6,122 | OR | 1.43 (1.06, 1.93) | 0.187 | 80 (51-88) | <0.001 | 0.165 | NP | 0.56-3.69 |
| prostate cancer (51) | Circulating Folate | General | Case-control and cohort | 10mmol/L increase | 7 | 9,353 | 2,958 | OR | 1.11 (0.96, 1.28) | 0.160 | 39 (0-73) | 0.132 | 0.334 | 0.001 | 0.79-1.55 |
| prostate cancer (49) | dietary folate intake | General | Case-control and cohort | Highest vs lowest | 11 | 120,349 | 14,290 | OR | 0.98 (0.90, 1.07) | 0.603 | 45 (0-71) | 0.053 | 0.279 | 0.228 | 0.79-1.21 |
| prostate cancer (48) | serum folate | General | prospective cohort | Highest vs lowest | 5 | 36,243 | 5,489 | RR | 1.21 (1.05, 1.39) | 0.008 | 0 (0-64) | 0.724 | 0.428 | NP | 0.96-1.52 |
| prostate cancer (49) | total folate intake | General | case-control and cohort | Highest vs lowest | 5 | 93,781 | 7,114 | OR | 0.99 (0.82, 1.19) | 0.886 | 49 (0-79) | 0.100 | 0.404 | NP | 0.58-1.68 |
| Renal Cell Cancer (52) | folate supplement | General | Case-control and cohort | Highest vs lowest | 8 | 374,901 | 2,723 | RR | 0.89 (0.77, 1.02) | 0.099 | 0 (0-56) | 0.872 | 0.856 | NP | 0.75-1.06 |
| acute lymphoblastic leukemia (53) | folate supplement | 1 month before pregnancy | case-control | Folate supplement vs. no | 2 | 2,042 | 490 | OR | 1.06 (0.77, 1.46) | 0.736 | 0 | 0.920 | NA | NP | NA |
| **Cardiovascular outcomes** | | | | | | | | | | | | | | | |
| coronary heart disease (54) | blood folate | General | prospective cohort | Highest vs lowest | 8 | 14,533 | 1,936 | RR | 0.74 (0.53, 1.02) | 0.068 | 65 (14-81) | 0.002 | 0.262 | NP | 0.27-2.01 |
| coronary heart disease (54) | dietary folate intake | General | prospective cohort | Highest vs lowest | 7 | 221,009 | 2,682 | RR | 0.69 (0.60, 0.80) | 9.87E-07 | 0 (0-56) | 0.470 | 0.955 | NP | 0.58-0.83 |
| **Neurocognitive disorders** | | | | | | | | | | | | | | | |
| Alzheimer's disease (55) | serum folate | General | cohort | Lower vs higher | 6 | 2,070 | 476 | RR | 2.22 (1.71, 2.89) | 2.73E-09 | 0 (0-61) | 0.861 | 0.519 | NP | 1.53-3.22 |
| cognitive impairment (56) | serum folate | Seniors | cross-sectional and cohort studies | Lower vs higher | 13 | 10,104 | NA | OR | 1.66 (1.40, 1.96) | 2.98E-09 | 45 (0-70) | 0.040 | 0.907 | NP | 1.04-2.64 |
| Depression (57) | circulating folate | General | cross-sectional, case–control and cohort | Highest vs lowest | 10 | 15,215 | 1,769 | OR | 1.58 (1.17, 2.14) | 0.003 | 44 (0-71) | 0.068 | 0.482 | NP | 0.72-3.45 |
| Depression (58) | serum folate | General | Case-control, cohort and, cross-sectional | Highest vs lowest | 11 | 7,949 | 1,783 | OR | 1.22 (1.02, 1.46) | 0.033 | 30 (0-64) | 0.150 | 0.490 | 0.411 | 0.79-1.87 |
| Parkinson's disease(59) | dietary folate intake | General | Case-control and cohort | Highest vs lowest | 3 | 143,643 | 794 | OR | 1.06 (0.78, 1.45) | 0.714 | 0 (0-73) | 0.486 | 0.387 | NP | 0.14-7.97 |
| **Pregnancy outcomes** | | | | | | | | | | | | | | | |
| gestational hypertension(60) | folate supplement | Any use | cohort | Folate supplement vs. no | 4 | 1,202,706 | 77,859 | RR | 1.03 (0.98, 1.09) | 0.267 | 80 (39-90) | <0.001 | 0.620 | NP | 0.87-1.23 |
| gestational hypertension/preeclampsia(61) | folate supplement | Pregnancy | cohort | Highest vs lowest | 9 | 279,286 | 147,424 | RR | 0.92 (0.79, 1.09) | 0.341 | 80 (60-88) | <0.001 | 0.178 | NP | 0.57-1.48 |
| preeclampsia(60) | folate supplement | Any use | cohort | Folate supplement vs. no | 7 | 1,242,398 | 43,818 | ES | 0.99 (0.90, 1.08) | 0.738 | 75 (37-86) | <0.001 | 0.777 | NP | 0.78-1.24 |
| **Other outcomes** | | | | | | | | | | | | | | | |
| Serrated Colorectal Polyps(62) | dietary folate intake | Adults undergoing endoscopic investigation | Case-control and cohort | Highest vs lowest | 3 | 32,462 | 831 | RR | 0.65 (0.50, 0.85) | 0.001 | 34 (0-81) | 0.222 | 0.409 | NP | 0.05-7.71 |

### Supplementary Table S4. AMSTAR 2 scores for different health outcomes in meta-analysis of observational studies.

| **Outcomes** | **Exposure** | **Population** | **Comparison** | **Study design** | **1** | **2** | **3** | **4** | **5** | **6** | **7** | **8** | **9** | **10** | **11** | **12** | **13** | **14** | **15** | **16** | **Rating** |
| --- | --- | --- | --- | --- | --- | --- | --- | --- | --- | --- | --- | --- | --- | --- | --- | --- | --- | --- | --- | --- | --- |
| **All cause and cause specific mortality** | | | | | | | | | | | | | | | | | | | | | |
| All-cause mortality (1) | Dietary folate intake | Breast cancer patients | Highest vs lowest | Prospective cohort | Yes | No | No | PY | Yes | Yes | Yes | PY | NRSI  Yes | No | NRSI  Yes | Yes | Yes | Yes | Yes | Yes | Low |
| All-cause mortality (1) | Total folate intake | Breast cancer patients | Highest vs lowest | Prospective cohort | Yes | No | No | PY | Yes | Yes | Yes | PY | NRSI  Yes | No | NRSI  Yes | Yes | Yes | Yes | Yes | Yes | Low |
| Breast cancer mortality (1) | Dietary folate intake | Breast cancer patients | Highest vs lowest | Prospective cohort | Yes | No | No | PY | Yes | Yes | Yes | PY | NRSI  Yes | No | NRSI  Yes | Yes | Yes | Yes | Yes | Yes | Low |
| **Birth outcomes** | | | | | | | | | | | | | | | | | | | | | |
| Asthma (60) | folate supplement | Pregnancy | Folate supplement vs. no | Cohort | Yes | No | No | PY | Yes | Yes | Yes | PY | NRSI  Yes | No | NRSI  Yes | Yes | Yes | Yes | Yes | Yes | Low |
| asthma or wheezing (3) | folate supplement | Pregnancy with periconceptional period | Folate supplement vs. no | Cohort | Yes | PY | No | PY | Yes | Yes | Yes | PY | NRSI  Yes | No | NRSI  Yes | Yes | Yes | Yes | Yes | Yes | Moderate |
| atopic dermatitis (2) | folate supplement | Early pregnancy | Folate supplement vs. no | Cohort | Yes | No | No | PY | Yes | Yes | Yes | PY | NRSI  Yes | No | NRSI  Yes | Yes | Yes | Yes | Yes | Yes | Low |
| autism spectrum disorders (5) | folate supplement | pregnancy | Folate supplement vs. no | Cohort and case-control | Yes | No | No | PY | Yes | Yes | Yes | PY | No | No | NRSI  Yes | Yes | Yes | Yes | Yes | Yes | CL |
| All cleft (6) | folate supplement | Pregnancy | Folate supplement vs. no | Case-control | Yes | No | No | No | Yes | Yes | No | PY | No | No | NRSI  Yes | No | Yes | No | Yes | No | CL |
| All cleft (6) | folate supplement | Pregnancy | Folate supplement vs. no | Cohort | Yes | No | No | No | Yes | Yes | No | PY | No | No | NRSI  Yes | No | Yes | No | Yes | No | CL |
| Cleft lip with or without cleft palate (8) | folate supplement | pregnancy | folate supplement vs. control | Case-control and cohort | Yes | Yes | No | PY | Yes | Yes | Yes | PY | NRSI  Yes | No | NRSI  Yes | Yes | Yes | Yes | Yes | Yes | Moderate |
| Cleft lip with or without cleft palate (Prevalence) (9) | folate supplement | Pregnancy | Folate supplement vs. no | Case-control | Yes | No | No | PY | Yes | Yes | Yes | PY | NRSI  Yes | No | NRSI  Yes | Yes | Yes | Yes | Yes | Yes | Low |
| Cleft palate (8) | folate supplement | pregnancy | folate supplement vs. control | Case-control and cohort | Yes | Yes | No | PY | Yes | Yes | Yes | PY | NRSI  Yes | No | NRSI  Yes | Yes | Yes | Yes | Yes | Yes | Moderate |
| Cleft palate (prevalence) (9) | folate supplement | Pregnancy | Folate supplement vs. no | Case-control | Yes | No | No | PY | Yes | Yes | Yes | PY | NRSI  Yes | No | NRSI  Yes | Yes | Yes | Yes | Yes | Yes | Low |
| Congenital Heart Defects (11) | folate supplement | General | Folate supplement vs. no | Case-control and cohort | Yes | No | No | PY | Yes | Yes | Yes | PY | NRSI  Yes | No | NRSI  Yes | Yes | Yes | Yes | Yes | Yes | Low |
| Eczema (2) | folate supplement | Other period in pregnancy | Folate supplement vs. no | Cohort | Yes | No | No | PY | Yes | Yes | Yes | PY | NRSI  Yes | No | NRSI  Yes | Yes | Yes | Yes | Yes | Yes | Low |
| Low birthweight (13) | folate intake | Post-conception pregnancy | Folate supplement vs. no | Cohort | Yes | Yes | Yes | PY | Yes | Yes | Yes | PY | No | No | No | No | Yes | Yes | No | Yes | CL |
| Low birthweight (13) | folate intake | Pre-conception pregnancy | Folate supplement vs. no | Cohort | Yes | Yes | Yes | PY | Yes | Yes | Yes | PY | No | No | No | No | Yes | Yes | No | Yes | CL |
| Neural tube defects (14) | folate supplement | pregnancy | Folate supplement vs. no | before and after studies | Yes | No | No | PY | Yes | Yes | Yes | Yes | NRSI  Yes | No | NRSI  Yes | Yes | Yes | Yes | No | Yes | CL |
| Neural tube defects (prevalence) (15) | folate supplement | Women with a previous pregnancy with NTD indicates | Highest vs lowest | Case-control | Yes | No | No | PY | No | No | Yes | PY | No | No | NRSI  Yes | Yes | Yes | Yes | No | Yes | CL |
| Neural tube disorders recurrent (12) | folate supplement | Pregnancy | Folate supplement vs. no | Case-control | Yes | No | No | PY | Yes | Yes | Yes | No | No | No | NRSI  Yes | No | No | No | No | Yes | CL |
| Non-syndromic cleft lip with or without cleft palate (9) | folate supplement | Pregnancy | Folate supplement vs. no | Case-control | Yes | No | No | PY | Yes | Yes | Yes | PY | NRSI  Yes | No | NRSI  Yes | Yes | Yes | Yes | Yes | Yes | Low |
| Non-syndromic cleft palate only (9) | folate supplement | Pregnancy | Folate supplement vs. no | Case-control | Yes | No | No | PY | Yes | Yes | Yes | PY | NRSI  Yes | No | NRSI  Yes | Yes | Yes | Yes | Yes | Yes | Low |
| Non-syndromic orofacial clefts (9) | folate supplement | Pregnancy | Folate supplement vs. no | Case-control | Yes | No | No | PY | Yes | Yes | Yes | PY | NRSI  Yes | No | NRSI  Yes | Yes | Yes | Yes | Yes | Yes | Low |
| Orofacial clefts (9) | folate supplement | Pregnancy | Folate supplement vs. no | Case-control | Yes | No | No | PY | Yes | Yes | Yes | PY | NRSI  Yes | No | NRSI  Yes | Yes | Yes | Yes | Yes | Yes | Low |
| Preterm delivery (16) | folate supplement | pregnancy | High dose folate supplement vs no | Cohort | Yes | No | Yes | PY | Yes | Yes | Yes | PY | NRSI  Yes | No | NRSI  Yes | Yes | Yes | Yes | No | Yes | CL |
| Preterm delivery (16) | folate supplement | pregnancy | Moderate to low dose folate vs no | Cohort | Yes | No | Yes | PY | Yes | Yes | Yes | PY | NRSI  Yes | No | NRSI  Yes | Yes | Yes | Yes | No | Yes | CL |
| Sensitization (2) | folate supplement | Other period in pregnancy | Folate supplement vs. no | Cohort | Yes | No | No | PY | Yes | Yes | Yes | PY | NRSI  Yes | No | NRSI  Yes | Yes | Yes | Yes | Yes | Yes | Low |
| Small for gestational age (16) | folate supplement | pregnancy | Moderate to low dose folate vs no | Cohort | Yes | No | Yes | PY | Yes | Yes | Yes | PY | NRSI  Yes | No | NRSI  Yes | Yes | Yes | Yes | No | Yes | CL |
| Wheeze (2) | folate supplement | Any Pregnancy | Folate supplement vs. no | Cohort | Yes | No | No | PY | Yes | Yes | Yes | PY | NRSI  Yes | No | NRSI  Yes | Yes | Yes | Yes | Yes | Yes | Low |
| Wheeze (2) | folate supplement | Early pregnancy | Folate supplement vs. no | Cohort | Yes | No | No | PY | Yes | Yes | Yes | PY | NRSI  Yes | No | NRSI  Yes | Yes | Yes | Yes | Yes | Yes | Low |
| Wheeze (2) | folate supplement | Other period in pregnancy | Folate supplement vs. no | Cohort | Yes | No | No | PY | Yes | Yes | Yes | PY | NRSI  Yes | No | NRSI  Yes | Yes | Yes | Yes | Yes | Yes | Low |
| **Cancer outcomes** | | | | | | | | | | | | | | | | | | | | | |
| Bladder cancer (17) | total folate intake | General | Highest vs lowest | case-control + cohort | Yes | No | No | PY | Yes | Yes | Yes | PY | NRSI  Yes | No | NRSI  Yes | Yes | Yes | Yes | Yes | Yes | Low |
| Breast cancer (20) | blood folate | General | Highest vs lowest | case-control | Yes | No | No | PY | Yes | Yes | Yes | PY | NRSI  Yes | No | NRSI  Yes | Yes | Yes | Yes | Yes | Yes | Low |
| Breast cancer (20) | blood folate | General | Highest vs lowest | cohort | Yes | No | No | PY | Yes | Yes | Yes | PY | NRSI  Yes | No | NRSI  Yes | Yes | Yes | Yes | Yes | Yes | Low |
| Breast cancer (21) | dietary folate intake | General | Highest vs lowest | cohort | Yes | No | No | PY | Yes | Yes | No | PY | No | No | NRSI  Yes | Yes | Yes | Yes | Yes | No | CL |
| breast cancer (22) | dietary folate intake | Postmenopausal women | 100- μ g/d increase | case-control | Yes | No | No | PY | Yes | Yes | No | PY | No | No | NRSI  Yes | No | Yes | No | Yes | No | CL |
| breast cancer (22) | dietary folate intake | Postmenopausal women | 100- μ g/d increase | cohort | Yes | No | No | PY | Yes | Yes | No | PY | No | No | NRSI  Yes | No | Yes | No | Yes | No | CL |
| breast cancer (22) | dietary folate intake | Premenopausal women | 100- μ g/d increase | case-control | Yes | No | No | PY | Yes | Yes | No | PY | No | No | NRSI  Yes | No | Yes | No | Yes | No | CL |
| breast cancer (22) | dietary folate intake | Premenopausal women | 100- μ g/d increase | cohort | Yes | No | No | PY | Yes | Yes | No | PY | No | No | NRSI  Yes | No | Yes | No | Yes | No | CL |
| breast cancer (20) | folate supplement | General | Highest vs lowest | cohort | Yes | No | No | PY | Yes | Yes | Yes | PY | NRSI  Yes | No | NRSI  Yes | Yes | Yes | Yes | Yes | Yes | Low |
| breast cancer (23) | total folate intake | General | Highest vs lowest | case-control and cohort | Yes | No | Yes | PY | Yes | Yes | Yes | PY | No | No | NRSI  Yes | No | No | Yes | Yes | Yes | CL |
| cervical cancer (24) | serum folate | General | Deficient vs. normal | case-control | Yes | No | No | PY | Yes | Yes | Yes | PY | NRSI  Yes | No | NRSI  Yes | Yes | Yes | Yes | Yes | Yes | Low |
| cervical neoplasm (25) | folate intake and serum folate | General | Folate supplement vs. no | case–control | Yes | No | Yes | PY | Yes | Yes | Yes | PY | No | No | NRSI  Yes | No | Yes | Yes | No | Yes | CL |
| colon cancer (26) | dietary folate intake | General | Highest vs lowest | prospective study | Yes | No | Yes | No | No | No | No | PY | No | No | NRSI  Yes | No | Yes | Yes | No | No | CL |
| colon cancer (26) | folate intake and ciuculating folate | General | Highest vs lowest | prospective study | Yes | No | Yes | No | No | No | No | PY | No | No | NRSI  Yes | No | Yes | Yes | No | No | CL |
| Colorectal adenoma (27) | Circulating folate | General | Lowest vs highest | case-control + cross sectional + cohort + clinical trials | Yes | No | No | No | Yes | Yes | Yes | PY | No | No | NRSI  Yes | No | No | Yes | No | Yes | CL |
| Colorectal cancer (28) | circulating folate | General | Highest vs lowest | cohort | Yes | No | No | No | No | No | Yes | PY | No | No | NRSI  Yes | No | Yes | Yes | No | Yes | CL |
| Colorectal cancer (30) | dietary folate intake | General | Highest vs lowest | case-control | Yes | No | No | PY | Yes | Yes | Yes | PY | No | No | NRSI  Yes | No | Yes | Yes | Yes | Yes | CL |
| Colorectal cancer (30) | dietary folate intake | General | Highest vs lowest | cohort | Yes | No | No | PY | Yes | Yes | Yes | PY | No | No | NRSI  Yes | No | Yes | Yes | Yes | Yes | CL |
| Colorectal cancer (31) | folate supplement | General | Highest vs lowest | prospective cohort | Yes | No | Yes | PY | Yes | Yes | Yes | PY | No | No | NRSI  Yes | No | Yes | Yes | Yes | Yes | CL |
| Colorectal cancer (33) | folate supplement | Patients with Inflammatory Bowel Disease | Folate supplement vs. no | case-control +cohort | Yes | No | No | PY | Yes | Yes | Yes | PY | NRSI  Yes | No | NRSI  Yes | Yes | Yes | Yes | Yes | No | CL |
| Colorectal cancer (32) | RBC folate | General | Folate supplement vs. no | case-control | Yes | No | Yes | PY | No | No | Yes | PY | NRSI  Yes | No | Yes | Yes | Yes | Yes | Yes | Yes | CL |
| colorectal cancer (34) | total folate intake | General | Highest vs lowest | cohort studies | Yes | No | No | No | Yes | Yes | Yes | PY | NRSI  Yes | No | NRSI  Yes | Yes | Yes | Yes | Yes | Yes | CL |
| endometrial cancer (35) | total folate intake | General | Highest vs lowest | case-control and cohort | Yes | No | No | PY | Yes | Yes | Yes | PY | No | No | NRSI  Yes | No | Yes | Yes | Yes | Yes | CL |
| esophageal adenocarcinoma (36) | dietary folate intake | General | Highest vs lowest | case-control | Yes | No | No | No | No | No | No | PY | No | No | NRSI  Yes | No | Yes | No | Yes | Yes | CL |
| esophageal cancer (37) | serum folate | General | Highest vs lowest | case-control and cohort | Yes | No | No | PY | Yes | Yes | Yes | PY | NRSI  Yes | No | NRSI  Yes | Yes | Yes | Yes | Yes | Yes | Low |
| esophageal cancer (37) | dietary folate intake | General | Highest vs lowest | case-control and cohort | Yes | No | No | PY | Yes | Yes | Yes | PY | NRSI  Yes | No | NRSI  Yes | Yes | Yes | Yes | Yes | Yes | Low |
| esophageal cancer (41) | folate intake and serum folate | General | Highest vs lowest | case-control + cohort | Yes | No | No | PY | Yes | Yes | Yes | PY | No | No | NRSI  Yes | No | Yes | Yes | Yes | No | CL |
| gastric cancer (39) | dietary folate intake | General | Highest vs lowest | cohort | Yes | No | No | PY | Yes | Yes | Yes | PY | No | No | NRSI  Yes | No | Yes | Yes | Yes | Yes | CL |
| gastric cancer (41) | folate intake and serum folate | General | Highest vs lowest | case-control + cohort | Yes | No | No | PY | Yes | Yes | Yes | PY | No | No | NRSI  Yes | No | Yes | Yes | Yes | No | CL |
| Head and neck squamous cell carcinoma (42) | folate intake and serum folate | General | Highest vs lowest | case-control | Yes | No | No | PY | Yes | Yes | Yes | PY | NRSI  Yes | No | NRSI  Yes | Yes | Yes | Yes | Yes | Yes | Low |
| Lung Cancer(44) | dietary folate intake | General | Highest vs lowest | cohort | Yes | No | No | PY | Yes | Yes | Yes | PY | NRSI  Yes | No | NRSI  Yes | Yes | Yes | Yes | Yes | No | CL |
| Lung Cancer (43) | serum folate | General | Highest vs lowest | case-control + cohort | Yes | No | No | PY | Yes | Yes | Yes | PY | No | No | NRSI  Yes | No | No | Yes | Yes | No | CL |
| Lung Cancer (43) | total folate intake | General | Highest vs lowest | case-control | Yes | No | No | PY | Yes | Yes | Yes | PY | No | No | NRSI  Yes | No | No | Yes | Yes | No | CL |
| ovarian cancer (45) | dietary folate intake | General | Highest vs lowest | case control+cohort | Yes | No | No | PY | Yes | Yes | Yes | PY | No | No | NRSI  Yes | No | Yes | Yes | Yes | Yes | CL |
| pancreatic cancer (46) | blood folate | General | Highest vs lowest | case-control + cohort | Yes | No | No | PY | Yes | Yes | No | PY | No | No | NRSI  Yes | No | Yes | No | Yes | Yes | CL |
| pancreatic cancer (39) | dietary folate intake | General | Highest vs lowest | cohort | Yes | No | No | PY | Yes | Yes | Yes | PY | No | No | NRSI  Yes | No | Yes | Yes | Yes | Yes | CL |
| pancreatic cancer (41) | folate intake and serum folate | General | Highest vs lowest | case-control + cohort | Yes | No | No | PY | Yes | Yes | Yes | PY | No | No | NRSI  Yes | No | Yes | Yes | Yes | No | CL |
| pancreatic cancer (46) | folate supplement | General | Highest vs lowest | case-control + cohort | Yes | No | No | PY | Yes | Yes | No | PY | No | No | NRSI  Yes | No | Yes | No | Yes | Yes | CL |
| prostate cancer (49) | blood folate | General | Highest vs lowest | case-control and cohort | Yes | No | Yes | PY | Yes | No | Yes | PY | No | No | NRSI  Yes | No | No | Yes | Yes | Yes | CL |
| prostate cancer (51) | Circulating Folate | General | 10mmol/L increase | case-control + cohort | Yes | No | No | PY | Yes | Yes | No | No | No | No | NRSI  Yes | No | No | Yes | No | Yes | CL |
| prostate cancer (49) | dietary folate intake | General | Highest vs lowest | case+cohort | Yes | No | Yes | PY | Yes | No | Yes | PY | No | No | NRSI  Yes | No | No | Yes | Yes | Yes | CL |
| prostate cancer (48) | serum folate | General | Highest vs lowest | prospective cohort | Yes | No | Yes | PY | Yes | Yes | Yes | PY | NRSI  Yes | No | NRSI  Yes | Yes | Yes | Yes | Yes | Yes | Low |
| prostate cancer (49) | total folate intake | General | Highest vs lowest | case-control and cohort | Yes | No | Yes | PY | Yes | No | Yes | PY | No | No | NRSI  Yes | No | No | Yes | Yes | Yes | CL |
| Renal Cell Cancer (52) | folate supplement | General | Highest vs lowest | case-control+cohort | Yes | No | No | PY | Yes | Yes | Yes | PY | NRSI  Yes | No | NRSI  Yes | Yes | Yes | Yes | Yes | No | Low |
| acute lymphoblastic leukemia (53) | folate supplement | 1 month before pregnancy | Folate supplement vs. no | case-control | Yes | No | No | No | No | No | Yes | PY | No | No | No | No | Yes | No | No | No | CL |
| **Cardiovascular outcomes** | | | | | | | | | | | | | | | | | | | | | |
| coronary heart disease (54) | blood folate | General | Highest vs lowest | prospective cohort | Yes | No | No | PY | Yes | Yes | Yes | PY | No | No | NRSI  Yes | No | Yes | Yes | Yes | Yes | CL |
| coronary heart disease (54) | dietary folate intake | General | Highest vs lowest | prospective cohort | Yes | No | No | PY | Yes | Yes | Yes | PY | No | No | NRSI  Yes | No | Yes | Yes | Yes | Yes | CL |
| **Neurocognitive disorders** | | | | | | | | | | | | | | | | | | | | | |
| Alzheimer's disease (55) | serum folate | General | Lower vs higher | cohort | Yes | No | No | PY | No | Yes | Yes | PY | No | No | NRSI  Yes | No | No | Yes | No | No | CL |
| cognitive impairment (56) | serum folate | Seniors | Lower vs higher | cross-sectional and cohort studies | Yes | No | No | No | Yes | Yes | Yes | PY | No | No | NRSI  Yes | No | Yes | Yes | Yes | Yes | CL |
| Depression (57) | circulating folate | General | Highest vs lowest | Cross-sectional, case–control and cohort | Yes | No | No | PY | Yes | Yes | No | PY | No | No | NRSI  Yes | No | No | Yes | Yes | Yes | CL |
| Depression (58) | serum folate | General | Highest vs lowest | Case-control, cohort, and cross-sectional | Yes | No | No | No | Yes | Yes | Yes | PY | NRSI  Yes | No | NRSI  Yes | Yes | Yes | Yes | Yes | Yes | CL |
| Parkinson's disease (59) | dietary folate intake | General | Highest vs lowest | Case-control and cohort | Yes | No | No | No | No | No | Yes | PY | No | No | NRSI  Yes | No | No | No | Yes | Yes | CL |
| **Pregnancy outcomes** | | | | | | | | | | | | | | | | | | | | | |
| gestational hypertension (60) | folate supplement | Any use | Folate supplement vs. no | cohort | Yes | No | No | PY | Yes | Yes | Yes | PY | NRSI  Yes | No | NRSI  Yes | Yes | Yes | Yes | Yes | Yes | Low |
| gestational hypertension/preeclampsia (61) | folate supplement | Pregnancy | Highest vs lowest | cohort | Yes | No | No | PY | Yes | Yes | Yes | PY | NRSI  Yes | No | NRSI  Yes | Yes | Yes | Yes | Yes | Yes | Low |
| Preeclampsia (60) | folate supplement | Any use | Folate supplement vs. no | cohort | Yes | No | No | PY | Yes | Yes | Yes | PY | NRSI  Yes | No | NRSI  Yes | Yes | Yes | Yes | Yes | Yes | Low |
| **Other outcomes** | | | | | | | | | | | | | | | | | | | | | |
| Serrated Colorectal Polyps (62) | dietary folate intake | Adults undergoing endoscopic investigation | Highest vs lowest | Cohort and case-control studies | Yes | No | No | PY | Yes | Yes | Yes | PY | NRSI  Yes | No | NRSI  Yes | Yes | Yes | Yes | Yes | Yes | Low |

PY=partial yes; CL=critically low; NRSI= non-randomized studies.

### Supplementary Table S5. Health outcomes and GRADE classification in meta-analysis of observational studies.

| **Outcomes** | **Exposure** | **Population** | **Comparison** | **N studies** | **RCT** | **Cohort** | **Case-control** | **Risk of Bias** | **Inconsistency** | **Indirectness** | **Imprecision** | **Publication bias** | **Large effect** | **Plausible Confounding** | **Dose-response** | **Quality** |
| --- | --- | --- | --- | --- | --- | --- | --- | --- | --- | --- | --- | --- | --- | --- | --- | --- |
| **All cause and cause specific mortality** | | | | | | | | | | | | | | | | |
| All-cause mortality (1) | Dietary folate intake | Breast cancer patients | Highest vs lowest | 5 | 0 | 5 | 0 | Serious risk | Serious inconsistency | No Serious Indirectness | No serious imprecision | Undetected | No | Would reduce effect | Yes | ⊕⊕〇〇 Low |
| All-cause mortality (1) | Total folate intake | Breast cancer patients | Highest vs lowest | 4 | 0 | 4 | 0 | Serious risk | No serious inconsistency | No Serious Indirectness | No serious imprecision | Undetected | No | Would not reduce effect | No | ⊕〇〇〇 Very Low |
| Breast cancer mortality (1) | Dietary folate intake | Breast cancer patients | Highest vs lowest | 2 | 0 | 2 | 0 | Serious risk | No serious inconsistency | No Serious Indirectness | No serious imprecision | Undetected | No | Would reduce effect | No | ⊕⊕〇〇 Low |
| **Birth outcomes** | | | | | | | | | | | | | | | | |
| Asthma (60) | folate supplement | Pregnancy | Folate supplement vs. no | 5 | 0 | 5 | 0 | Serious risk | No serious inconsistency | No Serious Indirectness | No serious imprecision | Undetected | No | Would not reduce effect | No | ⊕〇〇〇 Very Low |
| asthma or wheezing (3) | folate supplement | Pregnancy with periconceptional period | Folate supplement vs. no | 5 | 0 | 5 | 0 | Serious risk | No serious inconsistency | No Serious Indirectness | No serious imprecision | Undetected | No | would reduce effect | No | ⊕〇〇〇 Very Low |
| atopic dermatitis (2) | folate supplement | Early pregnancy | Folate supplement vs. no | 2 | 0 | 2 | 0 | Serious risk | No serious inconsistency | No Serious Indirectness | No serious imprecision | Undetected | No | would not reduce effect | No | ⊕〇〇〇 Very Low |
| autism spectrum disorders (5) | folate supplement | pregnancy | Folate supplement vs. no | 16 | 0 | 6 | 10 | Very serious risk | Serious inconsistency | No Serious Indirectness | No serious imprecision | Undetected | No | would reduce effect | No | ⊕〇〇〇 Very Low |
| All cleft (6) | folate supplement | Pregnancy | Folate supplement vs. no | 12 | 0 | 0 | 12 | Very serious risk | Serious inconsistency | No Serious Indirectness | No serious imprecision | Undetected | No | would reduce effect | No | ⊕〇〇〇 Very Low |
| All cleft (6) | folate supplement | Pregnancy | Folate supplement vs. no | 5 | 0 | 5 | 0 | Serious risk | No serious inconsistency | No Serious Indirectness | No serious imprecision | Undetected | No | would reduce effect | No | ⊕⊕〇〇 Low |
| Cleft lip with or without cleft palate (8) | folate supplement | pregnancy | folate supplement vs. control | 17 | 0 | 6 | 11 | Very serious risk | Serious inconsistency | No Serious Indirectness | No serious imprecision | Very Likely | No | Would not reduce effect | No | ⊕〇〇〇 Very Low |
| Cleft lip with or without cleft palate (Prevalence) (9) | folate supplement | Pregnancy | Folate supplement vs. no | 10 | 0 | 0 | 10 | Very serious risk | Serious inconsistency | No Serious Indirectness | No serious imprecision | Undetected | No | Would not reduce effect | No | ⊕〇〇〇 Very Low |
| Cleft palate (8) | folate supplement | pregnancy | folate supplement vs. control | 14 | 0 | 4 | 10 | Very serious risk | Very serious inconsistency | No Serious Indirectness | No serious imprecision | Very Likely | No | Would not reduce effect | No | ⊕〇〇〇 Very Low |
| Cleft palate (prevalence) (9) | folate supplement | Pregnancy | Folate supplement vs. no | 10 | 0 | 0 | 10 | Very serious risk | Very serious inconsistency | No Serious Indirectness | No serious imprecision | Undetected | No | Would not reduce effect | No | ⊕〇〇〇 Very Low |
| Congenital Heart Defects (11) | folate supplement | General | Folate supplement vs. no | 20 | 0 | 1 | 19 | Very serious risk | Very serious inconsistency | No Serious indirectness | No serious imprecision | Undetected | NO | Would reduce effect | No | ⊕⊕〇〇 Low |
| Eczema (2) | folate supplement | Other period in pregnancy | Folate supplement vs. no | 2 | 0 | 2 | 0 | Serious risk | No serious inconsistency | No Serious Indirectness | No serious imprecision | Undetected | No | would not reduce effect | No | ⊕〇〇〇 Very Low |
| Low birthweights (13) | folate intake | Post-conception pregnancy | Folate supplement vs. no | 2 | 0 | 2 | 0 | Serious risk | NA | No Serious Indirectness | Serious imprecision | NA | No | Would not reduce effect | No | ⊕⊕⊕〇 Moderate |
| Low birthweights (13) | folate intake | Pre-conception pregnancy | Folate supplement vs. no | 3 | 0 | 3 | 0 | Serious risk | NA | No Serious Indirectness | No serious imprecision | NA | No | Would reduce effect | No | ⊕⊕⊕〇 Moderate |
| Neural tube defects (14) | folate supplement | pregnancy | Folate supplement vs. no | 11 | 0 | 0 | 11 | Very serious risk | Very serious inconsistency | No Serious Indirectness | No serious imprecision | Undetected | No | would reduce effect | No | ⊕〇〇〇 Very Low |
| Neural tube defects (prevalence) (15) | folate supplement | Women with a previous pregnancy with NTD indicates | Highest vs lowest | 8 | 0 | 0 | 8 | Very serious risk | No serious inconsistency | No Serious Indirectness | No serious imprecision | Undetected | No | would reduce effect | No | ⊕〇〇〇 Very Low |
| Neural tube disorders recurrent (12) | folate supplement | Pregnancy | Folate supplement vs. no | 2 | 0 | 0 | 2 | NA | Serious inconsistency | No Serious Indirectness | NA | NA | NA | NA | NA | ⊕〇〇〇 Very Low |
| Non-syndromic cleft lip with or without cleft palate (9) | folate supplement | Pregnancy | Folate supplement vs. no | 3 | 0 | 0 | 3 | Very serious risk | No serious inconsistency | No Serious Indirectness | No serious imprecision | Undetected | No | Would reduce effect | No | ⊕⊕⊕⊕High |
| Non-syndromic cleft palate only (9) | folate supplement | Pregnancy | Folate supplement vs. no | 3 | 0 | 0 | 3 | Very serious risk | Serious inconsistency | No Serious Indirectness | No serious imprecision | Undetected | No | Would not reduce effect | No | ⊕⊕⊕〇 Moderate |
| Non-syndromic orofacial clefts (9) | folate supplement | Pregnancy | Folate supplement vs. no | 5 | 0 | 0 | 5 | Very serious risk | Serious inconsistency | No Serious Indirectness | No serious imprecision | Undetected | No | Would not reduce effect | No | ⊕〇〇〇 Very Low |
| Orofacial clefts (9) | folate supplement | Pregnancy | Folate supplement vs. no | 15 | 0 | 0 | 15 | Very serious risk | Serious inconsistency | No Serious Indirectness | No serious imprecision | Undetected | No | Would not reduce effect | No | ⊕〇〇〇 Very Low |
| Preterm delivery (16) | folate supplement | pregnancy | High dose folate supplement vs no | 2 | 0 | 0 | 2 | Serious risk | No serious inconsistency | No Serious Indirectness | No serious imprecision | NA | No | Would reduce effect | No | ⊕⊕〇〇 Low |
| Preterm delivery (16) | folate supplement | pregnancy | Moderate to low dose folate vs no | 7 | 0 | 0 | 7 | Serious risk | Serious inconsistency | No Serious Indirectness | No serious imprecision | NA | No | Would reduce effect | No | ⊕⊕〇〇 Low |
| Sensitization (2) | folate supplement | Other period in pregnancy | Folate supplement vs. no | 2 | 0 | 2 | 0 | Serious risk | No serious inconsistency | No Serious Indirectness | No serious imprecision | Undetected | No | would not reduce effect | No | ⊕〇〇〇 Very Low |
| Small for gestational age (16) | folate supplement | pregnancy | Moderate to low dose folate vs no | 6 | 0 | 0 | 6 | Serious risk | Serious inconsistency | No Serious Indirectness | No serious imprecision | NA | No | Would reduce effect | No | ⊕⊕⊕⊕High |
| Wheeze (2) | folate supplement | Any Pregnancy | Folate supplement vs. no | 2 | 0 | 2 | 0 | Serious risk | No serious inconsistency | No Serious Indirectness | No serious imprecision | Undetected | No | would not reduce effect | No | ⊕〇〇〇 Very Low |
| Wheeze (2) | folate supplement | Early pregnancy | Folate supplement vs. no | 3 | 0 | 3 | 0 | Serious risk | No serious inconsistency | No Serious Indirectness | No serious imprecision | Undetected | No | would reduce effect | No | ⊕⊕〇〇 Low |
| Wheeze (2) | folate supplement | Other period in pregnancy | Folate supplement vs. no | 3 | 0 | 3 | 0 | Serious risk | No serious inconsistency | No Serious Indirectness | No serious imprecision | Undetected | No | would not reduce effect | No | ⊕⊕⊕〇 Moderate |
| **Cancer outcomes** | | | | | | | | | | | | | | | | |
| bladder cancer (17) | total folate intake | General | Highest vs lowest | 13 | 0 | 7 | 6 | Very serious risk | Serious inconsistency | No Serious Indirectness | No serious imprecision | Undetected | No | would not reduce effect | No | ⊕〇〇〇 Very Low |
| breast cancer (20) | blood folate | General | Highest vs lowest | 4 | 0 | 0 | 4 | Very serious risk | Very serious inconsistency | No Serious Indirectness | Serious imprecision | Undetected | No | would not reduce effect | No | ⊕〇〇〇 Very Low |
| breast cancer (20) | blood folate | General | Highest vs lowest | 6 | 0 | 0 | 6 | Very serious risk | Serious inconsistency | No Serious Indirectness | No serious imprecision | Undetected | No | would not reduce effect | No | ⊕〇〇〇 Very Low |
| breast cancer (21) | dietary folate intake | General | Highest vs lowest | 15 | 0 | 15 | 0 | Serious risk | Serious inconsistency | No Serious Indirectness | No serious imprecision | Undetected | No | would not reduce effect | No | ⊕〇〇〇 Very Low |
| breast cancer (22) | dietary folate intake | Postmenopausal women | 100- μ g/d increase | 4 | 0 | 0 | 4 | Very serious risk | Serious inconsistency | No Serious Indirectness | No serious imprecision | Undetected | No | would not reduce effect | No | ⊕〇〇〇 Very Low |
| breast cancer (22) | dietary folate intake | Postmenopausal women | 100- μ g/d increase | 6 | 0 | 6 | 0 | Serious risk | Serious inconsistency | No Serious Indirectness | No serious imprecision | Undetected | No | would not reduce effect | No | ⊕〇〇〇 Very Low |
| breast cancer (22) | dietary folate intake | Premenopausal women | 100- μ g/d increase | 5 | 0 | 0 | 5 | Very serious risk | Serious inconsistency | No Serious Indirectness | No serious imprecision | Undetected | No | would reduce effect | Yes | ⊕〇〇〇 Very Low |
| breast cancer (22) | dietary folate intake | Premenopausal women | 100- μ g/d increase | 3 | 0 | 3 | 0 | Serious risk | Serious inconsistency | No Serious Indirectness | No serious imprecision | Undetected | No | would not reduce effect | No | ⊕〇〇〇 Very Low |
| breast cancer (20) | folate supplement | General | Highest vs lowest | 3 | 0 | 3 | 0 | Serious risk | No serious inconsistency | No Serious Indirectness | No serious imprecision | Undetected | No | would not reduce effect | No | ⊕〇〇〇 Very Low |
| breast cancer (23) | total folate intake | General | Highest vs lowest | 15 | 0 | 11 | 4 | Serious risk | Serious inconsistency | No Serious Indirectness | No serious imprecision | Undetected | No | would not reduce effect | No | ⊕〇〇〇 Very Low |
| cervical cancer (24) | serum folate | General | Deficient vs. normal | 6 | 0 | 0 | 6 | Very serious risk | Very serious inconsistency | No Serious Indirectness | Serious imprecision | Very Likely | Large | Would reduce effect | No | ⊕⊕⊕〇 Moderate |
| cervical neoplasm (25) | folate intake and serum folate | General | Folate supplement vs. no | 9 | 0 | 0 | 9 | Very serious risk | Serious inconsistency | No Serious Indirectness | No serious imprecision | Undetected | No | Would not reduce effect | No | ⊕〇〇〇 Very Low |
| colon cancer (26) | dietary folate intake | General | Highest vs lowest | 13 | 0 | 13 | 0 | Serious risk | Serious inconsistency | No Serious Indirectness | No serious imprecision | Undetected | No | would not reduce effect | No | ⊕〇〇〇 Very Low |
| colon cancer (26) | folate intake and ciuculating folate | General | Highest vs lowest | 8 | 0 | 8 | 0 | Serious risk | Serious inconsistency | No Serious Indirectness | No serious imprecision | Undetected | No | Would not reduce effect | No | ⊕〇〇〇 Very Low |
| Colorectal adenoma (27) | Circulating folate | General | Lowest vs highest | 11 | 4 | 1 | 6 | Very serious risk | Serious inconsistency | No Serious Indirectness | No serious imprecision | Undetected | No | Would reduce effect | Yes | ⊕⊕⊕〇 Moderate |
| Colorectal cancer (28) | circulating folate | General | Highest vs lowest | 8 | 0 | 8 | 0 | Serious risk | No serious inconsistency | No Serious Indirectness | No serious imprecision | NA | No | would not reduce effect | No | ⊕〇〇〇 Very Low |
| Colorectal cancer (30) | dietary folate intake | General | Highest vs lowest | 13 | 0 | 0 | 13 | Very serious risk | Serious inconsistency | No Serious Indirectness | No serious imprecision | Very Likely | No | would not reduce effect | No | ⊕⊕⊕〇 Moderate |
| Colorectal cancer (30) | dietary folate intake | General | Highest vs lowest | 9 | 0 | 9 | 0 | Serious risk | Serious inconsistency | No Serious Indirectness | No serious imprecision | Undetected | No | would not reduce effect | No | ⊕⊕〇〇 Low |
| Colorectal cancer (31) | folate supplement | General | Highest vs lowest | 3 | 0 | 3 | 0 | Serious risk | No serious inconsistency | No Serious Indirectness | No serious imprecision | Undetected | No | Would not reduce effect | No | ⊕〇〇〇 Very Low |
| Colorectal cancer (33) | folate supplement | Patients with Inflammatory Bowel Disease | Folate supplement vs. no | 10 | 0 | 4 | 6 | Very serious risk | Serious inconsistency | No Serious Indirectness | No serious imprecision | Undetected | No | would reduce effect | No | ⊕〇〇〇 Very Low |
| Colorectal cancer (32) | RBC folate | General | Folate supplement vs. no | 8 | 0 | 0 | 8 | Very serious risk | Serious inconsistency | No Serious Indirectness | No serious imprecision | Undetected | No | would not reduce effect | No | ⊕〇〇〇 Very Low |
| colorectal cancer (34) | total folate intake | General | Highest vs lowest | 23 | 0 | 23 | 0 | Serious risk | Serious inconsistency | No Serious Indirectness | No serious imprecision | Undetected | No | Would not reduce effect | No | ⊕〇〇〇 Very Low |
| endometrial cancer (35) | total folate intake | General | Highest vs lowest | 14 | 0 | 5 | 9 | Very serious risk | Serious inconsistency | No Serious Indirectness | No serious imprecision | Undetected | No | would not reduce effect | No | ⊕〇〇〇 Very Low |
| esophageal adenocarcinoma (36) | dietary folate intake | General | Highest vs lowest | 3 | 0 | 0 | 3 | Very serious risk | No serious inconsistency | No Serious Indirectness | No serious imprecision | Undetected | No | would reduce effect | No | ⊕⊕⊕〇 Moderate |
| esophageal cancer (37) | serum folate | General | Highest vs lowest | 4 | 0 | 2 | 2 | Very serious risk | Very serious inconsistency | No Serious Indirectness | No serious imprecision | Undetected | No | would reduce effect | No | ⊕⊕〇〇 Low |
| esophageal cancer (37) | dietary folate intake | General | Highest vs lowest | 15 | 0 | 1 | 14 | Very serious risk | Serious inconsistency | No Serious Indirectness | No serious imprecision | Undetected | No | would reduce effect | Yes | ⊕〇〇〇 Very Low |
| esophageal cancer (41) | folate intake and serum folate | General | Highest vs lowest | 19 | 0 | 2 | 17 | Very serious risk | Serious inconsistency | No Serious Indirectness | No serious imprecision | Very Likely | No | Would reduce effect | Yes | ⊕⊕⊕〇 Moderate |
| gastric cancer (39) | dietary folate intake | General | Highest vs lowest | 16 | 0 | 4 | 12 | Very serious risk | Serious inconsistency | No Serious Indirectness | No serious imprecision | Undetected | No | would not reduce effect | No | ⊕〇〇〇 Very Low |
| gastric cancer (41) | folate intake and serum folate | General | Highest vs lowest | 21 | 0 | 5 | 16 | Very serious risk | Serious inconsistency | No Serious Indirectness | No serious imprecision | Undetected | No | Would reduce effect | Yes | ⊕⊕⊕〇 Moderate |
| Head and neck squamous cell carcinoma (42) | folate intake and serum folate | General | Highest vs lowest | 9 | 0 | 0 | 9 | Very serious risk | Serious inconsistency | No Serious Indirectness | No serious imprecision | Undetected | No | Would reduce effect | No | ⊕⊕⊕〇 Moderate |
| Lung Cancer (44) | dietary folate intake | General | Highest vs lowest | 9 | 0 | 9 | 0 | Serious risk | No serious inconsistency | No Serious Indirectness | No serious imprecision | Undetected | No | Would not reduce effect | No | ⊕⊕⊕〇 Moderate |
| Lung Cancer (43) | serum folate | General | Highest vs lowest | 5 | 0 | 2 | 3 | Very serious risk | No serious inconsistency | No Serious Indirectness | Serious imprecision | Undetected | No | Would not reduce effect | No | ⊕⊕⊕⊕High |
| Lung Cancer (43) | total folate intake | General | Highest vs lowest | 7 | 0 | 0 | 7 | Very serious risk | No serious inconsistency | No Serious Indirectness | No serious imprecision | Undetected | NO | Would reduce effect | No | ⊕⊕〇〇 Low |
| ovarian cancer (45) | dietary folate intake | General | Highest vs lowest | 8 | 0 | 4 | 4 | Very serious risk | Serious inconsistency | No Serious Indirectness | No serious imprecision | Undetected | No | Would not reduce effect | No | ⊕⊕〇〇 Low |
| pancreatic cancer (46) | blood folate | General | Highest vs lowest | 3 | 0 | 3 | 0 | Serious risk | Serious inconsistency | No Serious Indirectness | No serious imprecision | Undetected | No | Would not reduce effect | No | ⊕〇〇〇 Very Low |
| pancreatic cancer (39) | dietary folate intake | General | Highest vs lowest | 8 | 0 | 5 | 3 | Very serious risk | Serious inconsistency | No Serious Indirectness | No serious imprecision | Undetected | No | would reduce effect | No | ⊕⊕〇〇 Low |
| pancreatic cancer (41) | folate intake and serum folate | General | Highest vs lowest | 12 | 0 | 7 | 5 | Very serious risk | Serious inconsistency | No Serious Indirectness | No serious imprecision | Undetected | No | Would reduce effect | Yes | ⊕⊕⊕〇 Moderate |
| pancreatic cancer (46) | folate supplement | General | Highest vs lowest | 4 | 0 | 3 | 1 | Serious risk | No serious inconsistency | No Serious Indirectness | No serious imprecision | Undetected | No | Would not reduce effect | No | ⊕〇〇〇 Very Low |
| prostate cancer (49) | blood folate | General | Highest vs lowest | 7 | 0 | 6 | 1 | Serious risk | Very serious inconsistency | No Serious Indirectness | No serious imprecision | Undetected | No | Would not reduce effect | No | ⊕〇〇〇 Very Low |
| prostate cancer (51) | Circulating Folate | General | 10mmol/L increase | 7 | 0 | 6 | 1 | Serious risk | Serious inconsistency | No Serious Indirectness | No serious imprecision | a | No | Would not reduce effect | No | ⊕⊕⊕〇 Moderate |
| prostate cancer (49) | dietary folate intake | General | Highest vs lowest | 11 | 0 | 5 | 6 | Very serious risk | Serious inconsistency | No Serious Indirectness | No serious imprecision | Undetected | No | would not reduce effect | No | ⊕〇〇〇 Very Low |
| prostate cancer (48) | serum folate | General | Highest vs lowest | 5 | 0 | 5 | 0 | Serious risk | No serious inconsistency | No Serious Indirectness | No serious imprecision | Undetected | No | would reduce effect | Yes | ⊕⊕⊕〇 Moderate |
| prostate cancer (49) | total folate intake | General | Highest vs lowest | 5 | 0 | 2 | 3 | Very serious risk | Serious inconsistency | No Serious Indirectness | No serious imprecision | Undetected | No | would not reduce effect | No | ⊕〇〇〇 Very Low |
| Renal Cell Cancer (52) | folate supplement | General | Highest vs lowest | 8 | 0 | 5 | 3 | Very serious risk | Serious inconsistency | No Serious Indirectness | No serious imprecision | Undetected | No | would not reduce effect | Yes | ⊕〇〇〇 Very Low |
| acute lymphoblastic leukemia (53) | folate supplement | 1 month before pregnancy | Folate supplement vs. no | 2 | 0 | 0 | 2 | Very serious risk | Serious inconsistency | No Serious Indirectness | No serious imprecision | Undetected | No | would not reduce effect | No | ⊕⊕⊕〇 Moderate |
| **Cardiovascular outcomes** | | | | | | | | | | | | | | | |  |
| coronary heart disease (54) | blood folate | General | Highest vs lowest | 8 | 0 | 8 | 0 | Serious risk | Serious inconsistency | No Serious Indirectness | No serious imprecision | Undetected | No | Would not reduce effect | No | ⊕⊕⊕〇 Moderate |
| coronary heart disease (54) | dietary folate intake | General | Highest vs lowest | 7 | 0 | 7 | 0 | Serious risk | No serious inconsistency | No Serious Indirectness | No serious imprecision | Undetected | No | Would reduce effect | Yes | ⊕⊕⊕〇 Moderate |
| **Neurocognitive disorders** | | | | | | | | | | | | | | | |  |
| Alzheimer's disease (55) | serum folate | General | Lower vs higher | 6 | 0 | 4 | 2 | Serious risk | No serious inconsistency | No Serious Indirectness | No serious imprecision | NA | Large | Would reduce effect | No | ⊕⊕⊕〇 Moderate |
| cognitive impairment (56) | serum folate | Seniors | Lower vs higher | 13 | 0 | 3 | 10 (cross-sectional) | Very serious risk | Serious inconsistency | No Serious Indirectness | No serious imprecision | Undetected | No | would reduce effect | No | ⊕〇〇〇 Very Low |
| Depression (57) | circulating folate | General | Highest vs lowest | 10 | 0 | 1 | 9 (include cross-sectional) | Very serious risk | Serious inconsistency | No Serious Indirectness | No serious imprecision | Undetected | Large | would reduce effect | No | ⊕〇〇〇 Very Low |
| Depression (58) | serum folate | General | Highest vs lowest | 11 | 0 | 1 | 10(include cross-sectional) | Very serious risk | Serious inconsistency | No Serious Indirectness | No serious imprecision | Undetected | No | Would reduce effect | No | ⊕〇〇〇 Very Low |
| Parkinson's disease (59) | dietary folate intake | General | Highest vs lowest | 3 | 0 | 2 | 1 | Serious risk | No serious inconsistency | No Serious Indirectness | Serious imprecision | NA | No | Would not reduce effect | No | ⊕⊕⊕〇 Moderate |
| **Pregnancy outcomes** | | | | | | | | | | | | | | | |  |
| gestational hypertension (60) | folate supplement | Any use | Folate supplement vs. no | 4 | 0 | 4 | 0 | Serious risk | Very serious inconsistency | No Serious Indirectness | No serious imprecision | Undetected | No | would not reduce effect | No | ⊕〇〇〇 Very Low |
| gestational hypertension/preeclampsia (61) | folate supplement | Pregnancy | Highest vs lowest | 9 | 0 | 9 | 0 | Serious risk | Serious inconsistency | No Serious Indirectness | No serious imprecision | a | No | Would not reduce effect | No | ⊕⊕⊕⊕High |
| Preeclampsia (60) | folate supplement | Any use | Folate supplement vs. no | 7 | 0 | 7 | 0 | Serious risk | Serious inconsistency | No Serious Indirectness | No serious imprecision | Undetected | No | would not reduce effect | No | ⊕〇〇〇 Very Low |
| **Other outcomes** | | | | | | | | | | | | | | | |  |
| Serrated Colorectal Polyps (62) | dietary folate intake | Adults undergoing endoscopic investigation | Highest vs lowest | 3 | 0 | 2 | 1 | Very serious risk | Serious inconsistency | No Serious Indirectness | No serious imprecision | Undetected | No | would reduce effect | No | ⊕⊕⊕〇 Moderate |

### Supplementary Table S6. General characteristics and main findings of the 144 meta-analysis of RCT studies.

| **Authors** | **Year** | **Outcome** | **Population** | **Versus** | **N Studies** | **N**  **Participants** | **N**  **Cases** | **Type of metric** | **Effect model reported** | **Reported summary effect (95% CI)** | **Recalculated summary effect (95% CI)** | **Concordance in overlapped MA** |
| --- | --- | --- | --- | --- | --- | --- | --- | --- | --- | --- | --- | --- |
| **All cause and cause specific mortality** | | | | | | | | | | | | |
| Schwingshack l(63) | 2017 | All-cause mortality | Preexisting diseases | Placebo/no treatment | 4 | 22,075 | 637 | RR | NA | 0.96 (0.75, 1.23) | NA | Yes (63-66) |
| Bazzano (64) | 2006 | All-cause mortality | Preexisting cardiovascular disease | Placebo/no treatment | 10 | 14,995 | 1,821 | RR | Fixed | 0.96 (0.88, 1.04) | NA |  |
| Yang (66) | 2012 | All-cause mortality | Preexisting diseases | Placebo/no treatment | 24 | 56,841 | 7,700 | RR | Fixed | 1.00 (0.96, 1.04) | 1.00 (0.96, 1.04) |  |
| Miller (65) | 2010 | All-cause mortality | Preexisting diseases | Placebo/no treatment | 12 | 4,211 | 2,110 | RR | Random | 1.01(0.95, 1.07) | NA |  |
| Qin (67) | 2012 | Cancer mortality | Preexisting diseases | Placebo/no treatment | 6 | 31,930 | 1,005 | RR | Random | 1.02 (0.90, 1.15) | 1.01 (0.90, 1.15) | Yes (47, 67) |
| Wien (47) | 2011 | Cancer mortality | Preexisting diseases | Placebo/no treatment | 5 | 32,327 | 1,134 | RR | Random | 1.09 (0.92, 1.3) | NA |  |
| **Birth outcomes** | | | | | | | | | | | | |
| De-Regil (68) | 2015 | anencephaly | Pregnancy | Placebo/no treatment | 4 | 4,807 | 17 | RR | Random | 0.36 (0.13, 1.03) | 0.35 (0.13, 0.97) |  |
| Fekete (69) | 2012 | Birth weight | Pregnancy | Placebo/no treatment | 9 | 707 |  | β | Random | 0.03 (0.01, 0.05) | 1.02 (1.01, 1.03) | No (69-71) (discordance in statistical significance) |
| Lassi (70) | 2016 | Birth weight | Pregnancy | Placebo/no treatment | 5 | 774 |  | MD | Random | 104.96 (-25.5, 235.41) | NA |  |
| Saccone (71) | 2016 | Birth weight | Pregnancy | Placebo/no treatment | 5 | NA |  | MD | NA | 85.58 (-55.17, 226.36) | NA |  |
| De-Regil (68) | 2015 | Cleft lip | Pregnancy | Placebo/no treatment | 3 | 5,612 | 8 | RR | Random | 0.79 (0.14, 4.36) | 0.73 (0.12, 4.41) |  |
| De-Regil (68) | 2015 | Cleft palate | Pregnancy | Placebo/no treatment | 3 | 5,612 | 3 | RR | Random | 0.73 (0.05, 10.89) | 0.73 (0.05, 10.56) |  |
| De-Regil (68) | 2015 | Congenital cardiovascular anomalies | Pregnancy | Placebo/no treatment | 3 | 5,612 | 22 | RR | Random | 0.57 (0.24, 1.33) | 0.54 (0.23, 1.27) |  |
| De-Regil (68) | 2015 | Elective termination of pregnancy for fetal anomalies | Pregnancy | Placebo/no treatment | 4 | 7,110 | 50 | RR | Random | 0.29 (0.15, 0.56) | 0.29 (0.15, 0.57) |  |
| De-Regil (68) | 2015 | Low birthweight | Pregnancy | Placebo/no treatment | 2 | 5,048 | 208 | RR | Random | 1.13 (0.84, 1.52) | NA | Yes (68, 70, 71) |
| Lassi (70) | 2016 | Low birthweight | Pregnancy | Placebo/no treatment | 4 | 3,113 | 273 | RR | Fixed | 0.83 (0.66, 1.04) | 0.89 (0.66, 1.18) |  |
| Saccone (71) | 2016 | Low birthweight | Pregnancy | Placebo/no treatment | 4 | NA | NA | RR | NA | 0.79 (0.49, 1.28) | NA |  |
| Lassi (70) | 2016 | Megaloblastic anaemia | Pregnancy | Placebo/no treatment | 4 | 3,839 | 89 | RR | Fixed | 0.21 (0.11, 0.38) | 0.26 (0.14, 0.46) |  |
| De-Regil (68) | 2015 | Neural tube defects | Pregnancy | Placebo/no treatment | 5 | 6,708 | 54 | RR | Random | 0.31 (0.17, 0.58) | 0.33 (0.18, 0.62) |  |
| Blencowe (15) | 2010 | Neural tube disorders recurrent | Pregnancy | Placebo/no treatment | 3 | 1,563 | 34 | RR | Fixed | 0.30 (0.14, 0.65) | 0.30 (0.14, 0.65) | Yes (12, 15) |
| Dean (12) | 2014 | Neural tube disorders recurrent | Pregnancy | Placebo/no treatment | 3 | 1,563 | 34 | RR | Random | 0.31 (0.14, 0.66) | NA |  |
| De-Regil (68) | 2015 | Other congenital anomalies | Pregnancy | Placebo/no treatment | 3 | 5,612 | 87 | RR | Random | 0.94 (0.53, 1.66) | 0.93 (0.53, 1.65) |  |
| Saccone (71) | 2016 | Perinatal death | Pregnancy | Placebo/no treatment | 2 | 4,002 | 101 | RR | Fixed | 0.90 (0.60, 1.34) | 0.92 (0.58, 1.47) |  |
| Fekete (69) | 2012 | Placental weight | Pregnancy | Placebo/no treatment | 5 | 198 |  | β | Random | 0.04 (0, 0.09) | 1.03 (1.00, 1.06) |  |
| Lassi (70) | 2016 | Preterm birth | Pregnancy | Placebo/no treatment | 3 | 2,959 |  | RR | Fixed | 1.01 (0.73, 1.38) | 0.99 (0.71, 1.40) |  |
| De-Regil (68) | 2015 | Spina bifida | Pregnancy | Placebo/no treatment | 3 | 4,546 | 6 | RR | Random | 0.32 (0.06, 1.62) | 0.33 (0.06, 1.67) |  |
| De-Regil (68) | 2015 | Stillbirths | Pregnancy | Placebo/no treatment | 4 | 6,597 | 34 | RR | Random | 1.05 (0.54, 2.05) | 1.01 (0.51, 2.01) |  |
| Lassi (70) | 2016 | Stillbirths/neonatal deaths | Pregnancy | Placebo/no treatment | 3 | 3,110 | 120 | RR | Fixed | 1.33 (0.96, 1.85) | 1.34 (0.97, 1.85) |  |
| **Cancer outcomes** | | | | | | | | | | | | |
| Qin (67) | 2012 | Breast cancer | Preexisting diseases | Placebo/no treatment | 4 | 19,800 | 203 | RR | Random | 0.82 (0.63, 1.07) | 0.84 (0.63, 1.12) |  |
| Wien (47) | 2011 | Cancer incidence | Preexisting diseases | Placebo/no treatment | 9 | 38,233 | 3,515 | RR | Random | 1.07 (1.00, 1.14) | NA | No (47, 67, 72-74) (discordance in statistical significance) |
| Zhou (74) | 2011 | Cancer incidence | Preexisting diseases | Placebo/no treatment | 6 | 26,544 | 2,472 | RR | Fixed | 1.08 (0.98, 1.21) | NA |  |
| Vollset (73) | 2013 | Cancer incidence | Preexisting diseases | Placebo/no treatment | 13 | 49,621 | 3,713 | RR | NA | 1.06 (0.99, 1.13) | 1.05 (0.97, 1.15) |  |
| Qin (67) | 2012 | Cancer incidence | Preexisting diseases | Placebo/no treatment | 13 | 49,406 | 3,741 | RR | Random | 1.05 (0.99, 1.11) | NA |  |
| Baggott (72) | 2011 | Cancer incidence | Preexisting diseases | Placebo/no treatment | 6 | 26,385 | 2,416 | RR | NA | 1.21 (1.05, 1.39) | NA |  |
| Vollset (73) | 2013 | Colorectal adenoma | Preexisting diseases | Placebo/no treatment | 3 | 2,652 | 168 | RR | Random | 1.33 (0.98, 1.80) | NA |  |
| CARROLL (75) | 2010 | Colorectal adenoma recurrence | Patients with a history of adenomas | Placebo/no treatment | 3 | 840 | 310 | RR | Random | 0.93 (0.61, 1.41) | NA | Yes (75-78) |
| Figueiredo (76) | 2010 | Colorectal adenoma recurrence | patients with an adenoma history | Placebo/no treatment | 3 | 1,957 | 682 | RR | Random | 0.98 (0.82, 1.17) | NA |  |
| Ibrahim (77) | 2009 | Colorectal adenoma recurrence | patients with an adenoma history | Placebo/no treatment | 4 | 1,486 | 564 | OR | Fixed | 1.08 (0.87, 1.33) | 1.08 (0.87, 1.33) |  |
| Dijk (78) | 2015 | Colorectal adenoma recurrence | patients with an adenoma history | Placebo/no treatment | 4 | 1,315 | 444 | RR | Random | 0.93 (0.69, 1.25) | NA |  |
| Fife (79) | 2009 | Colorectal adenomatous lesion | General (folate supplementation upto 3 years) | Placebo/no treatment | 2 | 3,686 | 850 | OR | Fixed | 1.09 (0.93, 1.28) | 1.09 (0.93, 1.28) |  |
| Fife (79) | 2009 | Colorectal adenomatous lesion | General (folate supplementation for over 3 years) | Placebo/no treatment | 2 | 6,736 | 383 | OR | Fixed | 1.35 (1.06, 1.70) | 1.34 (1.06, 1.70) |  |
| Qin (80) | 2015 | Colorectal cancer | General | Placebo/no treatment | 8 | 34,598 | 381 | RR | Fixed | 1.00 (0.82, 1.22) | 1.00 (0.82, 1.22) | Yes (75, 80) |
| CARROLL (75) | 2010 | Colorectal cancer | General | Placebo/no treatment | 3 | 11,062 | 120 | RR | Random | 1.13 (0.77, 1.64) | NA |  |
| Wien (47) | 2011 | Colorectal cancer | Preexisting diseases | Placebo/no treatment | 8 | NA | NA | RR | NA | 1.00 (0.83, 1.21) | NA | Yes (32, 47, 67) |
| Moazzen (32) | 2017 | Colorectal cancer | Preexisting diseases | Placebo/no treatment | 11 | 37,182 | 653 | RR | Random | 1.07 (0.86, 1.43) | NA |  |
| Qin (67) | 2012 | Colorectal cancer | Preexisting diseases | Placebo/no treatment | 7 | 33,824 | 367 | RR | Random | 1.01 (0.82, 1.23) | NA |  |
| Wien (47) | 2011 | Hematological cancers | Preexisting diseases | Placebo/no treatment | 4 | NA | NA | RR | NA | 1.16 (0.76, 1.78) | 1.16 (0.55, 2.43) |  |
| Qin (67) | 2012 | Hematological malignancy | Preexisting diseases | Placebo/no treatment | 3 | 25,670 | 170 | RR | Random | 0.87 (0.64, 1.17) | 0.70 (0.24, 1.99) |  |
| Wien (47) | 2011 | Lung Cancer | Preexisting diseases | Placebo/no treatment | 5 | NA | NA | RR | NA | 1.11 (0.92, 1.33) | 1.07 (0.88, 1.29) | Yes (47, 67) |
| Qin (67) | 2012 | Lung Cancer | Preexisting diseases | Placebo/no treatment | 5 | 31,864 | 447 | RR | Random | 1.00 (0.84, 1.21) | NA |  |
| Qin (67) | 2012 | Melanoma | Preexisting diseases | Placebo/no treatment | 3 | 19,128 | 38 | RR | Random | 0.47 (0.23, 0.94) | 0.51 (0.24, 1.10) |  |
| Wien (47) | 2011 | Prostate cancer | Preexisting diseases | Placebo/no treatment | 6 | 25,738 | 632 | RR | Random | 1.24 (1.03, 1.49) | 1.24 (1.04, 1.49) | No (47, 67) (discordance in statistical significance) |
| Qin (67) | 2012 | Prostate cancer | Preexisting diseases | Placebo/no treatment | 5 | 27,065 | 508 | RR | Random | 1.17 (0.84, 1.62) | NA |  |
| Figueiredo (76) | 2010 | Advanced colorectal lesion | Patients with an adenoma history | Placebo/no treatment | 3 | 1,922 | 202 | RR | Random | 1.0 6(0.81, 1.39) | 1.07 (0.82, 1.39) |  |
| **Cardiovascular outcomes** | | | | | | | | | | | | |
| Myung (81) | 2013 | Angina | Preexisting diseases | Placebo/no treatment | 4 | NA | NA | RR | Random | 0.93 (0.72, 1.20) | NA |  |
| Zhou (74) | 2011 | Cardiovascular disease | Preexisting diseases | Placebo/no treatment | 12 | 38,015 | 8,238 | RR | Fixed | 0.98 (0.93, 1.04) | NA | No (64-66, 73, 74, 81-83) (discordance in statistical significance) |
| Bazzano (64) | 2006 | Cardiovascular disease | Preexisting diseases | Placebo/no treatment | 10 | 14,440 | 2,706 | RR | Fixed | 0.95 (0.88, 1.03) | NA |  |
| Vollset (73) | 2016 | Cardiovascular disease | Preexisting diseases | Placebo/no treatment | 10 | 46,969 | 3,545 | RR | Random | 1.04 (0.98, 1.11) | NA |  |
| Yang (66) | 2012 | Cardiovascular disease | Preexisting diseases | Placebo/no treatment | 23 | 54,607 | 9,613 | RR | Fixed | 0.98 (0.95, 1.02) | NA |  |
| Li (82) | 2016 | Cardiovascular disease | Preexisting diseases | Placebo/no treatment | 22 | 74,346 | 9,739 | RR | Fixed | 0.96 (0.92, 0.99) | 0.94 (0.90, 0.99) |  |
| Qin (83) | 2011 | Cardiovascular disease | Preexisting diseases | Placebo/no treatment | 7 | 3,886 | 822 | RR | Random | 0.85 (0.76, 0.96) | NA |  |
| Miller (65) | 2010 | Cardiovascular disease | Preexisting diseases | Placebo/no treatment | 8 | 6,661 | 3,357 | RR | Random | 1.02 (0.98, 1.06) | NA |  |
| Myung (81) | 2013 | Cardiovascular disease | Preexisting diseases | Placebo/no treatment | 17 | NA | NA | RR | Fixed | 0.99 (0.96, 1.03) | NA |  |
| Myung (81) | 2013 | Cardiovascular disease | Preexisting diseases | Placebo/no treatment | 4 | NA | NA | RR | Fixed | 0.89 (0.78, 1.03) | NA |  |
| Schwingshackl (63) | 2017 | Cardiovascular events | General | Placebo/no treatment | 3 | 21,257 | NA | RR | Fixed | 0.81 (0.70, 0.94) | NA | Yes (63, 74, 81) |
| Zhou (74) | 2011 | Cardiovascular events | Preexisting diseases | Placebo/no treatment | 14 | 44,340 | 6,314 | RR | Fixed | 1.00 (0.96, 1.05) | NA |  |
| Myung (81) | 2013 | Cardiovascular events | Preexisting diseases | Placebo/no treatment | 21 | 57,592 | 9,531 | RR | Fixed | 0.99 (0.95, 1.02) | 0.98 (0.93, 1.03) |  |
| Qin (84) | 2011 | Carotid intima-media thickness | Preexisting diseases | Placebo/no treatment | 10 | 2,052 |  | MD | Random | -0.04 (-0.07, -0.02) | 0.92 (0.89, 0.96) |  |
| Zhou (74) | 2011 | Coronary syndrom | Preexisting diseases | Placebo/no treatment | 5 | 19,050 | 3,148 | RR | NA | 1.06 (0.97, 1.15) | 0.98 (0.85, 1.14) |  |
| Qin (85) | 2014 | Coronary artery bypass grafting | General | Placebo/no treatment | 5 | 10,703 | 811 | RR | Random | 0.90 (0.79, 1.03) | 0.90 (0.78, 1.03) |  |
| Bazzano (64) | 2006 | Coronary heart disease | Preexisting diseases | Placebo/no treatment | 11 | 16,877 | 1,862 | RR | Fixed | 1.04 (0.92, 1.17) | NA | Yes (64-66, 82) |
| Yang (66) | 2012 | Coronary heart disease | Preexisting diseases | Placebo/no treatment | 21 | 54,678 | 5,866 | RR | Fixed | 1.03 (0.98, 1.08) | NA |  |
| Li (82) | 2016 | Coronary heart disease | Preexisting diseases | Placebo/no treatment | 25 | 78,192 | 5,899 | RR | Fixed | 1.04 (0.99, 1.09) | 1.04 (0.99, 1.09) |  |
| Miller (65) | 2010 | Coronary heart disease | Preexisting diseases | Placebo/no treatment | 12 | 5,151 | 2,600 | RR | Random | 1.04 (0.94, 1.16) | NA |  |
| Qin (85) | 2014 | Coronary restenosis | General | Placebo/no treatment | 3 | 926 | 224 | RR | Random | 1.04 (0.52, 2.11) | 0.52 (0.32, 0.85) |  |
| Qin (85) | 2014 | Coronary revascularization | General | Placebo/no treatment | 9 | 27,418 | 2,575 | RR | Random | 0.99 (0.88, 1.11) | 0.99 (0.89, 1.11) |  |
| McRae (86) | 2009 | Diastolic blood pressure | hypertensive patients | Placebo/no treatment | 8 | 293 |  | MD | Random | 0.01 (-0.12, 1.13) | NA |  |
| YI (87) | 2014 | Diastolic blood pressure | patients with coronary artery disease | Placebo/no treatment | 4 | 237 |  | MD | Fixed | 0.08 (-2.10, 2.27) | 1.17 (0.02, 60.47) |  |
| Tabrizi (88) | 2017 | Diastolic blood pressure | patients with metabolic diseases | Placebo/no treatment | 6 | 262 |  | SMD | Random | -0.59 (-1.55 ,0.37) | 0.34 (0.06, 1.96) |  |
| YI (87) | 2014 | End diastolic diameter | patients with coronary artery disease | Placebo/no treatment | 4 | 237 |  | MD | Fixed | -0.03 (-0.2,0 0.15) | 0.95 (0.69, 1.31) |  |
| Tabrizi (88) | 2017 | HDL-cholesterol | patients with metabolic diseases | Placebo/no treatment | 10 | 492 |  | SMD | Random | 0.04 (-0.35 ,0.44) | 1.08 (0.52, 2.23) |  |
| YI (87) | 2014 | Heart rate | patients with coronary artery disease | Placebo/no treatment | 4 | 237 |  | MD | Fixed | -0.39 (-2.89, 2.11) | 0.49 (0.01, 45.64) |  |
| Tabrizi (88) | 2017 | LDL-cholesterol | patients with metabolic diseases | Placebo/no treatment | 9 | 432 |  | SMD | Random | 0.14 (-0.55, 0.28) | 0.78 (0.37, 1.64) |  |
| Zhou (74) | 2011 | Myocardial infarction | Preexisting diseases | Placebo/no treatment | 11 | 2,917 | 39,923 | RR | NA | 1.00 (0.93, 1.07) | 0.99 (0.93, 1.07) | Yes (74, 81) |
| Myung (81) | 2013 | Myocardial infarction | Preexisting diseases | Placebo/no treatment | 15 | NA | NA | RR | Fixed | 0.99 (0.93, 1.06) | NA |  |
| Qin (85) | 2014 | Percutaneous coronary intervention | General | Placebo/no treatment | 5 | 10,703 | 1,592 | RR | Random | 1.05 (0.89, 1.23) | 0.97 (0.84, 1.13) |  |
| Miller (65) | 2010 | Primary cardiovascular clinical end point | Preexisting diseases | Placebo/no treatment | 14 | 19,497 | 7,265 | RR | Random | 1.02 (0.93, 1.13) | 1.02 (0.95, 1.09) |  |
| Qin (85) | 2014 | Revascularization | General | Placebo/no treatment | 7 | 29,314 | 2,979 | RR | Random | 1.06 (0.99, 1.13) | 1.06 (0.99, 1.13) |  |
| Zhou (74) | 2011 | Revascularization | Preexisting diseases | Placebo/no treatment | 10 | 38,068 | 2,939 | RR | NA | 1.05 (0.95, 1.16) | 1.10 (0.96, 1.26) |  |
| Li (82) | 2016 | Stroke | Preexisting diseases | Placebo/no treatment | 20 | 77,816 | 3,164 | RR | Fixed | 0.90 (0.84, 0.96) | NA | No (64-66, 81, 82, 89-94) (discordance in statistical significance) |
| Bazzano (64) | 2006 | Stroke | preexisting disease | Placebo/no treatment | 8 | 13,806 | 722 | RR | Fixed | 0.86 (0.71, 1.04) | NA |  |
| Yang (66) | 2012 | Stroke | Preexisting diseases | Placebo/no treatment | 18 | 54,682 | 2,486 | RR | Fixed | 0.93 (0.86, 1.00) | NA |  |
| Wang (92) | 2007 | Stroke | Preexisting diseases | Placebo/no treatment | 8 | 16,841 | 778 | RR | Random | 0.82 (0.68, 1.00) | NA |  |
| Huo (89) | 2012 | Stroke | Preexisting diseases | Placebo/no treatment | 15 | 55,764 | 2,517 | RR | Random | 0.91 (0.83, 1.00) | NA |  |
| Lee (90) | 2010 | Stroke | Preexisting diseases | Placebo/no treatment | 13 | 39,005 | 1,575 | RR | Fixed | 0.93 (0.85, 1.03) | NA |  |
| Myung (81) | 2013 | Stroke | Preexisting diseases | Placebo/no treatment | 7 | NA | NA | RR | Fixed | 0.90 (0.79, 1.01) | NA |  |
| Tian (91) | 2017 | Stroke | Preexisting diseases | Placebo/no treatment | 11 | 62,832 | 2,826 | RR | Fixed | 0.90 (0.84, 0.97) | NA |  |
| Zhao (93) | 2017 | Stroke | Preexisting diseases | Placebo/no treatment | 22 | 82,723 | 3,308 | RR | Fixed | 0.89 (0.84, 0.96) | 0.88 (0.81 ,0.97) |  |
| Miller (65) | 2010 | Stroke | Preexisting diseases | Placebo/no treatment | 7 | 1,425 | 696 | RR | Random | 0.95 (0.84, 1.08) | NA |  |
| Hsu (94) | 2018 | Stroke | Preexisting diseases | Placebo/no treatment | 13 | 65,812 | 2,235 | RR | Random | 0.85 (0.77, 0.95) | NA |  |
| McRae (86) | 2009 | Systolic blood pressure | hypertensive patients | Placebo/no treatment | 8 | 293 |  | MD | Random | -2.03 (-3.63, -0.43) | NA |  |
| YI (87) | 2014 | Systolic blood pressure | patients with coronary artery disease | Placebo/no treatment | 4 | 237 |  | MD | Fixed | -1.07 (-5.71, 3.03) | 0.14 (0,24, 6.78) |  |
| Tabrizi (88) | 2017 | Systolic blood pressure | patients with metabolic diseases | Placebo/no treatment | 6 | 262 |  | SMD | Random | -0.87 (-1.83, 0.09) | 0.21 (0.04, 1.18) |  |
| Tabrizi (88) | 2017 | Total cholesterol | patients with metabolic diseases | Placebo/no treatment | 10 | 492 |  | SMD | Random | 0.06 (-0.31, 0.43) | 1.03 (0.56, 1.87) |  |
| Myung (81) | 2013 | Transient ischemic attack | Preexisting diseases | Placebo/no treatment | 2 | NA | NA | RR | Fixed | 1.12 (0.88, 1.42) | NA |  |
| Tabrizi (88) | 2017 | Triglycerides | patients with metabolic diseases | Placebo/no treatment | 11 | 232 |  | SMD | Random | 0.10 (-0.42, 0.63) | 0.98 (0.31, 3.11) |  |
| Tabrizi (88) | 2017 | VLDL-cholesterol | patients with metabolic diseases | Placebo/no treatment | 3 | 71 |  | SMD | Random | 0.08 (-0.24, 0.41) | 1.15 (0.64, 2.19) |  |
| **Endothelial function** | | | | | | | | | | | | |
| YI (87) | 2014 | Flow-mediated dilation | patients with coronary artery disease | Placebo/no treatment | 4 | 287 |  | MD | Fixed | 57.72 (50.14, 65.31) | NA |  |
| McRae (86) | 2009 | Flow-mediated dilation | hypertensive patients | Placebo/no treatment | 8 | 358 |  | OR | Random | 1.61 (1.27, 1.96) | NA |  |
| Bree (95) | 2007 | Flow-mediated dilatation | General | Placebo/no treatment | 14 | 732 |  | OR | Random | 1.08 (0.57, 1.59) | NA |  |
| YI (87) | 2014 | Glyceryl-trinitrate (GTN) diameter change | patients with coronary artery disease | Placebo/no treatment | 3 | 187 |  | MD | Fixed | 1.74 (-17.13, 20.61) | 23.39 (0, 1.60E+16) |  |
| YI (87) | 2014 | Peak hyperemic flow | patients with coronary artery disease | Placebo/no treatment | 3 | 187 |  | MD | Fixed | -2.25 (-23.32, 18.82) | 0.23(0, 4.60E+16) |  |
| **Neurocognitive disorders** | | | | | | | | | | | | |
| Wald (96) | 2010 | Cognitive function test scores | General | Placebo/no treatment | 9 | 71 |  | SMD | NA | 0.01 (-0.08, 0.01) | 1.02 (0.87, 1.20) |  |
| Schefft (97) | 2017 | Endpoint scores of the respective rating scales | depressive disorder | Placebo/no treatment | 3 | 567 |  | SMD | Random | -0.26 (-0.67, 0.15) | 0.62 (0.30, 1.31) |  |
| Ranganathan (98) | 2005 | Epilepsy seizure frequency | General | Placebo/no treatment | 2 | 75 | 18 | OR | Fixed | 0.98 (0.32, 2.98) | 0.98 (0.32, 2.98) |  |
| [Taylor](https://www.ncbi.nlm.nih.gov/pubmed/?term=Taylor%20MJ%5BAuthor%5D&cauthor=true&cauthor_uid=15260915) (99) | 2004 | Hamilton Depression Rating Scale score | depressive disorder | Placebo/no treatment | 2 | 124 | NA | MD | Fixed | -2.65 (-4.93, -0.38) | 0.01 (0, 0.51) |  |
| Roberts (100) | 2018 | BDI: Beck Depression Inventory/ HAMD: Hamilton Depression Scale | patients with unipolar depressive illness | Placebo/no treatment | 4 | 657 | NA | SMD | Random | -0.40 (-0.88, 0.08) | NA |  |
| **Pregnancy outcomes** | | | | | | | | | | | | |
| Hua (61) | 2016 | gestational hypertension/preeclampsia | Pregnancy | Placebo/no treatment | 2 | 3,774 | 155 | RR | NA | 0.62 (0.45, 0.87) | 0.62 (0.44, 0.89) |  |
| Fekete (69) | 2012 | Length of gestation | Pregnancy | Placebo/no treatment | 6 | 380 |  | β | Random | 0 (-0.01, 0.01) | 1.002 (0.997, 1.01) |  |
| Lassi (70) | 2016 | Low pre-delivery serum folate | Pregnancy | Placebo/no treatment | 2 | 696 | 75 | RR | Fixed | 0.38 (0.25, 0.59) | 0.41 (0.28, 0.62) |  |
| De-Regil (68) | 2015 | Miscarriage | Pregnancy | Placebo/no treatment | 5 | 7,391 | 769 | RR | Random | 1.10 (0.94, 1.28) | 1.09 (0.93, 1.29) |  |
| De-Regil (68) | 2015 | Multiple pregnancy | Pregnancy | Placebo/no treatment | 4 | 7,280 | 82 | RR | Random | 1.38 (0.89, 2.14) | 1.39 (0.89, 2.15) |  |
| Lassi (70) | 2016 | Pre-delivery anaemia | Pregnancy | Placebo/no treatment | 8 | 4,149 | 577 | RR | Random | 0.62 (0.35, 1.10) | 0.62 (0.36, 1.09) |  |
| Lassi (70) | 2016 | Pre-delivery hemoglobin level | Pregnancy | Placebo/no treatment | 12 | 1,806 |  | MD | Random | -0.03 (-0.25, 0.19) | 0.95 (0.64, 1.42) |  |
| Lassi (70) | 2016 | Pre-delivery serum folate | Pregnancy | Placebo/no treatment | 8 | 1,250 | NA | SMD | Random | 2.03 (0.80, 3.27) | 39.86 (4.27, 372.09) |  |
| **Diabetes related outcomes** | | | | | | | | | | | | |
| Akbari (101) | 2018 | Fasting Glucose | Metabolic Diseases | Placebo/no treatment | 10 | 521 |  | SMD | Random | -0.30 (-0.63, 0.02) | NA |  |
| Zhao (102) | 2018 | Fasting Glucose | General | Placebo/no treatment | 15 | 16,768 |  | MD | Random | -0.15 (-0.29, -0.01) | NA |  |
| Sudchada (103) | 2012 | HbA1c levels | type 2 diabetes | Placebo/no treatment | 3 | 142 | NA | MD | Random | -0.37 (-1.10, 0.35) | 0.51 (0.14, 1.89) |  |
| Akbari (101) | 2018 | HbA1c levels | Metabolic Diseases | Placebo/no treatment | 6 | 288 | NA | SMD | Random | -0.29 (-0.61, 0.03) | NA |  |
| Zhao (102) | 2018 | HbA1c levels | General | Placebo/no treatment | 4 | 313 | NA | MD | Random | -0.17 (-0.49, 0.16) | NA |  |
| Akbari (101) | 2018 | HOMA-IR | Metabolic Diseases | Placebo/no treatment | 9 | 494 |  | SMD | Random | -1.07 (-1.80, -0.33) | NA |  |
| Zhao (102) | 2018 | HOMA-IR | General | Placebo/no treatment | 9 | 435 |  | MD | Random | -0.83 (-1.31, -0.34) | NA |  |
| Akbari (101) | 2018 | Insulin | Metabolic Diseases | Placebo/no treatment | 8 | 463 |  | SMD | Random | -1.28 (-1.99, -0.56) | NA |  |
| Zhao (102) | 2018 | Insulin | General | Placebo/no treatment | 8 | 380 |  | MD | Random | -1.94 (-3.28, -0.61) | NA |  |
| **Other outcomes** | | | | | | | | | | | | |
| Zhou (74) | 2011 | Amputation | Preexisting diseases | Placebo/no treatment | 2 | 2,294 | 116 | RR | NA | 1.14 (0.80, 1.62) | 2.10 (0.20, 22.62) |  |
| YI (87) | 2014 | Baseline hyperemic flow | patients with coronary artery disease | Placebo/no treatment | 3 | 187 |  | MD | Fixed | 1.02 (-4.81, 6.84) | 6.29 (0, 240000) |  |
| Ranganathan (98) | 2005 | Dental Hyperplasia index | General | Placebo/no treatment | 2 | 28 |  | MD | Fixed | 0.01 (-0.24, 0.26) | 2.10 (0.20, 22.62) |  |
| Ranganathan (98) | 2005 | Dental Plaque index | General | Placebo/no treatment | 2 | 28 |  | MD | Fixed | -0.02 (-0.46, 0.42) | 6.29 (0, 240000) |  |
| Ranganathan (98) | 2005 | Gingival health index | General | Placebo/no treatment | 2 | 28 |  | MD | Fixed | -0.01 (-0.14, 0.11) | 1.02 (0.65, 1.60) |  |
| Shea (104) | 2014 | Liver toxicity | patients receiving methotrexate | Placebo/no treatment | 2 | 302 | 65 | RR | Fixed | 0.19 (0.1, 0.36) | 0.97 (0.43, 2.15) |  |
| Shea (104) | 2014 | Nausea / GI upset | patients receiving methotrexate | Placebo/no treatment | 3 | 355 | 123 | RR | Fixed | 0.76 (0.57, 1.01) | 0.99 (0.79, 1.25) |  |
| Shea (104) | 2014 | Neutropenia | patients receiving methotrexate | Placebo/no treatment | 2 | 302 | 8 | RR | Fixed | 1.70 (0.42, 6.96) | 0.20 (0.11, 0.36) |  |
| Sudchada (103) | 2012 | Plasma homocysteine | type 2 diabetes | Placebo/no treatment | 4 | 183 |  | MD | Random | -3.52 (-4.44, -2.6) | 0.77 (0.58, 1.03) |  |
| Berti (105) | 2012 | Plasma homocysteine | childbearing age, pregnant and lactating women | Placebo/no treatment | 9 | 683 |  | β | Random | -0.10 (-0.17, -0.04) | 1.68 (0.40, 6.98) |  |
| CLARKE (106) | 2000 | plasma homocysteine | General | Placebo/no treatment | 9 | 1,114 |  | MD | NA | 0.25 (0.23, 0.28) | NA | NA (106, 107) |
| Homocysteine Lowering Trialists’ Collaboration (107) | 2005 | Plasma homocysteine | General | Placebo/no treatment | 25 | 3,001 | NA | OR | NA | NA | 0.79 (0.76, 0.81) |  |
| YI (87) | 2014 | Plasma homocysteine | patients with coronary artery disease | Placebo/no treatment | 4 | 327 |  | MD | Random | -3.66 (-5.44, -1.87) | 0.002 (0, 0.025) |  |
| Miller (65) | 2010 | Plasma homocysteine | Preexisting diseases | Placebo/no treatment | 12 | 38,418 |  | MD | NA | -2.90 (-3.40,- 2.40) | NA |  |
| Berti (105) | 2012 | Red blood cell folate | childbearing age, pregnant and lactating women | Placebo/no treatment | 9 | 692 |  | β | Random | 0.56 (0.40, 0.72) | 1.47 (1.31, 1.66) |  |
| Duffy (108) | 2014 | Red blood cell folate | General | Placebo/no treatment | 7 | 1,441 | NA | OR | Random | 1.32 (1.25, 1.83) | 1.32 (1.26, 1.39) |  |
| Berti (105) | 2012 | Serum/plasma folate | childbearing age, pregnant and lactating women | Placebo/no treatment | 9 | 692 |  | β | Random | 0.56 (0.40, 0.72) | 1.47 (1.31, 1.65) |  |
| Duffy (108) | 2014 | Serum/plasma folate | General | Placebo/no treatment | 17 | 2,294 | NA | OR | Random | 1.56 (1.42, 1.69) | 1.55 (1.42, 1.70) |  |
| Shea (104) | 2014 | Stomatitis | patients receiving methotrexate | Placebo/no treatment | 2 | 302 | 45 | RR | Fixed | 0.90 (0.53, 1.54) | 0.89 (0.52, 1.52) |  |
| Shea (104) | 2014 | Total withdrawals | patients receiving methotrexate | Placebo/no treatment | 3 | 355 | 89 | RR | Fixed | 0.43 (0.29, 0.64) | 0.44 (0.29, 0.65) |  |

### Supplementary Table S7. Unique health outcomes reported in meta-analysis of randomized controlled trials.

| **Outcomes** | **Population** | **Versus** | **N studies** | **N participants** | **N cases** | **Type of metric** | **Relative Risk (95% CI)** | **P value** | ***I^2^***  **(95% CI)** | ***P* value for heterogeneity** | ***P* value for Egger test** | ***P* value for excess significance test** | **95% prediction interval** |
| --- | --- | --- | --- | --- | --- | --- | --- | --- | --- | --- | --- | --- | --- |
| **All cause and cause specific mortality** | | | | | | | | | | | | | |
| All-cause mortality (66) | Preexisting diseases | Placebo/no treatment | 24 | 56,841 | 7,700 | RR | 1.00 (0.96, 1.04) | 0.887 | 0 (0-40) | 0.499 | 0.152 | 0.506 | 0.96-1.05 |
| Cancer mortality (67) | Preexisting diseases | Placebo/no treatment | 6 | 31,930 | 1,005 | RR | 1.01 (0.90, 1.15) | 0.815 | 0 (0-61) | 0.807 | 0.404 | NP | 0.85-1.21 |
| **Birth outcomes** | | | | | | | | | | | | | |
| Anencephaly (68) | Pregnancy | Placebo/no treatment | 4 | 4,807 | 17 | RR | 0.35 (0.13, 0.97) | 0.044 | 0 (0-68) | 0.729 | 0.171 | NP | 0.04-3.30 |
| Birth weight (69) | Pregnancy | Placebo/no treatment | 9 | 707 | NA | β to OR | 1.02 (1.01, 1.03) | 5.85E-03 | 67 (16-82) | 0.002 | 0.498 | NP | 0.98-1.06 |
| Cleft lip (68) | Pregnancy | Placebo/no treatment | 3 | 5,612 | 8 | RR | 0.73 (0.12, 4.41) | 0.732 | NA | NA | NA | NP | NA |
| Cleft palate (68) | Pregnancy | Placebo/no treatment | 3 | 5,612 | 3 | RR | 0.73 (0.05, 10.56) | 0.820 | NA | NA | NA | NP | NA |
| Congenital cardiovascular anomalies (68) | Pregnancy | Placebo/no treatment | 3 | 5,612 | 22 | RR | 0.54 (0.23, 1.27) | 0.161 | 0 (0-73) | 0.427 | 0.417 | NP | 0.00-135.11 |
| Elective termination of pregnancy for fetal anomalies (68) | Pregnancy | Placebo/no treatment | 4 | 7,110 | 50 | RR | 0.29 (0.15, 0.57) | 2.80E-04 | 0 (0-68) | 0.931 | 0.418 | 0.890 | 0.07-1.25 |
| Low birthweight (70) | Pregnancy | Placebo/no treatment | 4 | 3,113 | 273 | RR | 0.89 (0.66, 1.18) | 0.413 | 18 (0-73) | 0.299 | 0.621 | NP | 0.36-2.08 |
| Megaloblastic anaemia (70) | Pregnancy | Placebo/no treatment | 4 | 3,839 | 89 | RR | 0.26 (0.14 ,0.46) | 4.96E-06 | 3 (0-69) | 0.379 | 0.207 | NP | 0.07-0.99 |
| Neural tube defects (68) | Pregnancy | Placebo/no treatment | 5 | 6,708 | 54 | RR | 0.33 (0.18, 0.62) | 5.98E-04 | 0 (0-64) | 0.741 | 0.833 | NP | 0.12-0.92 |
| Neural tube disorders recurrent (15) | Previous pregnancy with NTD indicates | Placebo/no treatment | 3 | 1,563 | 34 | RR | 0.30 (0.14, 0.65) | 2.13E-03 | 0 (0-73) | 0.852 | 0.884 | 0.772 | 0.00-41.70 |
| Other congenital anomalies (68) | Pregnancy | Placebo/no treatment | 3 | 5,612 | 87 | RR | 0.93 (0.53, 1.65) | 0.815 | 40 (0-82) | 0.190 | 0.664 | NP | 0.00-229.44 |
| Perinatal death (71) | Pregnancy | Placebo/no treatment | 2 | 4,002 | 101 | RR | 0.92 (0.58, 1.47) | 0.739 | NA | NA | NA | NP | NA |
| Placental weight (69) | Pregnancy | Placebo/no treatment | 5 | 198 | NA | β to OR | 1.03 (1.00, 1.06) | 0.056 | 67 (0-85) | 0.017 | 0.472 | NP | 0.94-1.13 |
| Preterm birth (70) | Pregnancy | Placebo/no treatment | 3 | 2,959 | NA | RR | 0.99 (0.71, 1.40) | 0.969 | 9 (0-71) | 0.350 | 0.078 | NP | 0.41-2.40 |
| Spina bifida (68) | Pregnancy | Placebo/no treatment | 3 | 4,546 | 6 | RR | 0.33 (0.06, 1.67) | 0.179 | 0 (0-73) | 0.925 | 0.625 | NP | 0.00-12503.42 |
| Stillbirths (68) | Pregnancy | Placebo/no treatment | 4 | 6,597 | 34 | RR | 1.01 (0.51, 2.01) | 0.979 | 4 (0-69) | 0.374 | 0.244 | NP | 0.20-5.22 |
| Stillbirths/neonatal deaths (70) | Pregnancy | Placebo/no treatment | 3 | 3,110 | 120 | RR | 1.34 (0.97, 1.85) | 0.078 | 0 (0-73) | 0.962 | 0.906 | NP | 0.16-11.00 |
| **Cancer outcomes** | | | | | | | | | | | | | |
| Advanced colorectal lesion (76) | patients with an adenoma history | Placebo/no treatment | 3 | 1,922 | 202 | RR | 1.07(0.82,1.39) | 0.641 | 2 (0-73) | 0.642 | 0.580 | NP | 0.18-6.27 |
| breast cancer (67) | Preexisting diseases | Placebo/no treatment | 4 | 19,800 | 203 | RR | NA | NA | NA | NA | NA | NA | NA |
| cancer incidence (73) | Preexisting diseases | Placebo/no treatment | 13 | 49,621 | 3,713 | RR | 1.05 (0.97, 1.15) | 0.223 | 0 (0-49) | 0.642 | 0.224 | NP | 0.96-1.16 |
| Colorectal adenoma (73) | Preexisting diseases | Placebo/no treatment | 3 | 2,652 | 168 | RR | 1.32 (0.88, 1.97) | 0.173 | 0 (0-73) | 0.725 | 0.557 | NP | 0.10-17.61 |
| colorectal adenoma recurrence (77) | General | Placebo/no treatment | 4 | 1,486 | 564 | OR | 1.08 (0.87, 1.33) | 0.482 | 0 (0-68) | 0.539 | 0.612 | NP | 0.68-1.72 |
| colorectal adenomatous lesion (79) (folate supplementation upto 3 years) | General | Placebo/no treatment | 2 | 3,686 | 850 | OR | 1.09 (0.93, 1.28) | 0.301 | 0 (0-68) | 0.691 | 0.610 | NP | 0.76-1.55 |
| colorectal adenomatous lesion (79) (folate supplementation for over 3 years) | General | Placebo/no treatment | 2 | 6,736 | 383 | OR | 1.34 (1.06, 1.70) | 0.014 | 0 (0-73) | 0.545 | 0.098 | NP | 0.29-6.19 |
| colorectal cancer (80) | General | Placebo/no treatment | 8 | 34,598 | 381 | RR | 1.00 (0.82, 1.22) | 0.979 | 0 (0-56) | 0.826 | 0.347 | NP | 0.78-1.29 |
| colorectal cancer (32) | Preexisting diseases | Placebo/no treatment | 11 | 37,182 | 653 | RR | (,) |  |  |  |  |  |  |
| Hematological cancers (47) | Preexisting diseases | Placebo/no treatment | 3 | NA | NA | RR | NA | NA | NA | NA | NA | NA | NA |
| hematological malignancy (67) | Preexisting diseases | Placebo/no treatment | 3 | 25,670 | 170 | RR | NA | NA | NA | NA | NA | NA | NA |
| Lung Cancer (47) | Preexisting diseases | Placebo/no treatment | 5 | NA | NA | RR | NA | NA | NA | NA | NA | NA | NA |
| Melanoma (67) | Preexisting diseases | Placebo/no treatment | 3 | 19,128 | 38 | RR | 0.51 (0.24, 1.10) | 0.086 | 0 (0-73) | 0.654 | 0.559 | NP | 0.00-71.33 |
| prostate cancer (47) | Preexisting diseases | Placebo/no treatment | 6 | 25,738 | 632 | RR | 1.24 (1.04, 1.49) | 0.020 | 16 (0-70) | 0.311 | 0.538 | 0.791 | 0.83-1.86 |
| **Cardiovascular outcomes** | | | | | | | | | | | | | |
| Angina (81) | Preexisting diseases | Placebo/no treatment | 4 | NA | NA | RR | NA | NA | NA | NA | NA | NA | NA |
| cardiovascular disease (82) | Preexisting diseases | Placebo/no treatment | 22 | 74,346 | 9,739 | RR | 0.94 (0.90, 0.99) | 0.016 | 19 (0-52) | 0.207 | 0.035 | NP | 0.84-1.05 |
| cardiovascular events (81) | Preexisting diseases | Placebo/no treatment | 21 | 57,592 | 9,531 | RR | 0.98 (0.93, 1.03) | 0.431 | 34 (0-60) | 0.063 | 0.915 | NP | 0.84-1.13 |
| Carotid intima-media thickness (84) | Preexisting diseases | Placebo/no treatment | 10 | 2,052 | NA | MD to OR | 0.92 (0.89, 0.96) | 4.36E-05 | 95 (93-96) | <0.001 | 0.044 | NP | 0.82-1.04 |
| Coronary syndrome (74) | Preexisting diseases | Placebo/no treatment | 5 | 19,050 | 3,148 | RR | NA | NA | NA | NA | NA | NA | NA |
| coronary artery bypass grafting (85) | General | Placebo/no treatment | 5 | 10,703 | 811 | RR | NA | NA | NA | NA | NA | NA | NA |
| coronary heart disease (82) | Preexisting diseases | Placebo/no treatment | 25 | 78,192 | 5,899 | RR | 1.04 (0.99, 1.09) | 0.157 | 0 (0-39) |  | 0.403 | NP | 0.98-1.09 |
| coronary restenosis (85) | General | Placebo/no treatment | 3 | 926 | 224 | RR | NA | NA | NA | NA | NA | NA | NA |
| coronary revascularization (85) | General | Placebo/no treatment | 9 | 27,418 | 2,575 | RR | 0.99 (0.89, 1.11) | 0.899 | 34 (0-69) | 0.719 | 0.370 | 0.297 | 0.77-1.28 |
| diastolic blood pressure (86) | hypertensive patients | Placebo/no treatment | 8 | 293 | NA | MD to OR | NA | NA | NA | NA | NA | NA | NA |
| diastolic blood pressure (87) | patients with coronary artery disease | Placebo/no treatment | 4 | 237 | NA | MD to OR | 1.17 (0.02, 60.47) | 0.939 | 0 (0-68) | 0.683 | 0.837 | NP | 0.00-6786.40 |
| diastolic blood pressure (88) | patients with metabolic diseases | Placebo/no treatment | 6 | 262 | NA | SMD to OR | 0.34 (0.06, 1.96) | 0.229 | 92 (86-95) | <0.001 | 0.223 | NP | 0.00-184.36 |
| end diastolic diameter (87) | patients with coronary artery disease | Placebo/no treatment | 4 | 237 | NA | MD to OR | 0.95 (0.69, 1.31) | 0.751 | 0 (0-68) | 0.997 | 0.641 | NP | 0.47-1.92 |
| HDL-cholesterol (88) | patients with metabolic diseases | Placebo/no treatment | 10 | 492 | NA | SMD to OR | 1.08 (0.52, 2.23) | 0.832 | 79 (57-87) | <0.001 | 0.155 | NP | 0.09-13.31 |
| heart rate (87) | patients with coronary artery disease | Placebo/no treatment | 4 | 237 | NA | MD to OR | 0.49 (0.01, 45.64) | 0.759 | 0 (0-68) | 0.737 | 0.731 | NP | 0.00-1026.62 |
| LDL-cholesterol (88) | patients with metabolic diseases | Placebo/no treatment | 9 | 432 | NA | SMD to OR | 0.78 (0.37, 1.64) | 0.513 | 77 (50-86) | <0.001 | 0.364 | NP | 0.06-9.58 |
| Myocardial infarction (74) | Preexisting diseases | Placebo/no treatment | 11 | 2,917 | 39,923 | RR | 0.99 (0.93, 1.07) | 0.881 | 0 (0-51) | 0.551 | 0.805 | NP | 0.92-1.08 |
| Percutaneous coronary intervention (85) | General | Placebo/no treatment | 5 | 10,703 | 1,592 | RR | NA | NA | NA | 0.183 | NA | NA | NA |
| Primary cardiovascular clinical end point r (65) | Preexisting diseases | Placebo/no treatment | 14 | 19,497 | 7,265 | RR | 1.02 (0.95, 1.09) | 0.573 | 38 (0-66) | 0.074 | 0.851 | 0.217 | 0.87-1.20 |
| Revascularization (85) | General | Placebo/no treatment | 7 | 29,314 | 2,979 | RR | 1.06 (0.99, 1.13) | 0.102 | 0 (0-58) | 0.723 | 0.025 | NP | 0.97-1.15 |
| Revascularization (74) | Preexisting diseases | Placebo/no treatment | 10 | 38,068 | 2,939 | RR | NA | NA | NA | NA | NA | NA | NA |
| Stroke (93) | Preexisting diseases | Placebo/no treatment | 22 | 82,723 | 3,308 | RR | 0.88 (0.81, 0.97) | 8.14E-03 | 24 (0-54) | 0.152 | 0.758 | 0.852 | 0.71-1.10 |
| systolic blood pressure (86) | hypertensive patients | Placebo/no treatment | 8 | 293 | NA | MD to OR | NA | NA | NA | NA | NA | NA | NA |
| systolic blood pressure (87) | patients with coronary artery disease | Placebo/no treatment | 4 | 237 | NA | MD to OR | 0.14 (0, 246.78) | 0.610 | 0 (0-68) | 0.965 | 0.914 | NP | 0.00-18.14E+5 |
| systolic blood pressure (88) | patients with metabolic diseases | Placebo/no treatment | 6 | 262 | NA | SMD to OR | 0.21 (0.04, 1.18) | 0.076 | 92 (86-95) | <0.001 | 0.136 | NP | 0.00-107.21 |
| total cholesterol (88) | patients with metabolic diseases | Placebo/no treatment | 10 | 492 | NA | SMD to OR | 1.03 (0.56, 1.87) | 0.933 | 72 (36-84) | <0.001 | 0.067 | NP | 0.14-7.64 |
| Transient ischemic attack (81) | Preexisting diseases | Placebo/no treatment | 2 | NA | NA | RR | NA | NA | NA | NA | NA | NA | NA |
| Triglycerides (88) | patients with metabolic diseases | Placebo/no treatment | 11 | 232 | NA | SMD to OR | 0.98 (0.31, 3.11) | 0.979 | 92 (87-94) | <0.001 | 0.513 | NP | 0.01-75.50 |
| VLDL-cholesterol (88) | patients with metabolic diseases | Placebo/no treatment | 3 | 71 | NA | SMD to OR | 1.15 (0.64, 2.19) | 0.633 | 0 (0-73) | 0.647 | 0.290 | NP | 0.02-53.36 |
| **Endothelial function** | | | | | | | | | | | | | |
| flow‑mediated dilation (87) | patients with coronary artery disease | Placebo/no treatment | 4 | 287 | NA | MD to OR | NA | NA | NA | NA | NA | NA | NA |
| flow-mediated dilation (86) | hypertensive patients | Placebo/no treatment | 8 | 358 | NA | OR | NA | NA | NA | NA | NA | NA | NA |
| Flow-mediated dilatation (95) | General | Placebo/no treatment | 14 | 732 | NA | OR | NA | NA | NA | NA | NA | NA | NA |
| glyceryl‑trinitrate (GTN) diameter change (87) | patients with coronary artery disease | Placebo/no treatment | 3 | 187 | NA | MD to OR | 23.39 (0, 1.60E+16) | 0.856 | 0 (0-73) | 0.867 | 0.600 | NP | NA |
| peak hyperemic flow (87) | patients with coronary artery disease | Placebo/no treatment | 3 | 187 | NA | MD to OR | 0.23 (0, 4.60E+16) | 0.942 | NA | NA | NA | NP | NA |
| **Neurocognitive disorders** | | | | | | | | | | | | | |
| cognitive function test scores (96) | General | Placebo/no treatment | 9 | 71 | NA | SMD to OR | 1.02 (0.87, 1.20) | 0.825 | 0 (0-54) | 0.569 | 0.939 | NP | 0.84-1.24 |
| endpoint scores of the respective rating scales (97) | depressive disorder | Placebo/no treatment | 3 | 567 | NA | SMD to OR | 0.62 (0.30, 1.31) | 0.211 | 68 (0-89) | 0.046 | 0.185 | NP | 0.00-2202.78 |
| epilepsy seizure frequency (98) | General | Placebo/no treatment | 2 | 75 | 18 | OR | 0.98 (0.32, 2.98) | 0.976 | 0 | 0.712 | NA | NP | NA |
| Hamilton Depression Rating Scale score (99) | depressive disorder | Placebo/no treatment | 2 | 124 | NA | MD to OR | 0.01 (0, 0.51) | 0.023 | NA | NA | NA | NP | NA |
| BDI: Beck Depression Inventory/ HAMD: Hamilton Depression Scale (100) | patients with unipolar depressive illness | Placebo/no treatment | 4 | 657 | NA | SMD to OR | 0.49 (0.21, 1.14) | 0.099 | 83 (37-92) | <0.001 | 0.101 | NP | 0.01-21.90 |
| **Pregnancy outcomes** | | | | | | | | | | | | | |
| gestational hypertension/preeclampsia (61) | Pregnancy | Placebo/no treatment | 2 | 3,774 | 155 | RR | 0.62 (0.44, 0.89) | 8.89E-03 | NA | NA | NA | 8.12E-04 | NA |
| Length of gestation (69) | Pregnancy | Placebo/no treatment | 6 | 380 | NA | β to OR | 1.002 (0.997, 1.01) | 0.505 | 0 (0-61) | 0.464 | 0.274 | NP | 0.99-1.01 |
| Low pre-delivery serum folate (70) | Pregnancy | Placebo/no treatment | 2 | 696 | 75 | RR | 0.41 (0.28, 0.62) | 1.99E-05 | NA | NA | NA | 0.474 | NA |
| Miscarriage (68) | Pregnancy | Placebo/no treatment | 5 | 7,391 | 769 | RR | 1.09 (0.93, 1.29) | 0.287 | 10 (0-67) | 0.352 | 0.359 | NP | 0.77-1.55 |
| Multiple pregnancy (68) | Pregnancy | Placebo/no treatment | 4 | 7,280 | 82 | RR | 1.39 (0.89, 2.15) | 0.145 | NA | NA | NA | NP | NA |
| Pre-delivery anaemia (70) | Pregnancy | Placebo/no treatment | 8 | 4,149 | 577 | RR | 0.62 (0.36, 1.09) | 0.096 | 89 (82-93) | <0.001 | 0.647 | 2.33E-04 | 0.10-3.39 |
| pre-delivery hemoglobin level (70) | Pregnancy | Placebo/no treatment | 12 | 1,806 | NA | MD to OR | 0.95 (0.64, 1.42) | 0.818 | 95 (94-96) | <0.001 | 0.945 | NP | 0.22-4.16 |
| pre-delivery serum folate (70) | Pregnancy | Placebo/no treatment | 8 | 1,250 | NA | SMD to OR | 39.86 (4.27, 372.09) | 1.22E-03 | NA | NA | 0.116 | NP | 0.01-133582.34 |
| **Diabetes related outcomes** | | | | | | | | | | | | | |
| Fasting Glucose (101) | Metabolic Diseases | Placebo/no treatment | 10 | 521 |  | SMD to OR | 0.58 (0.32, 1.04) | 0.066 | 69 (28-82) | NA | 0.731 | <1E-06 | 0.08-3.93 |
| Fasting Glucose (102) | General | Placebo/no treatment | 15 | 16,768 |  | MD to OR | 0.76 (0.59, 0.97) | 0.031 | 53 (0-73) | NA | 0.036 | NP | 0.39-1.48 |
| HbA1c (103) | type 2 diabetes | Placebo/no treatment | 3 | 142 | NA | MD to OR | 0.51 (0.14, 1.89) | 0.309 | 84 (12-93) | 0.002 | 0.142 | NP | 0.00-3564667.75 |
| HbA1c (101) | Metabolic Diseases | Placebo/no treatment | 6 | 288 |  | SMD to OR | 0.59 (0.33, 1.04) | 0.070 | 40 (0-75) | NA | 0.353 | NP | 0.13-2.61 |
| HbA1c (102) | General | Placebo/no treatment | 4 | 313 |  | MD to OR | 0.74 (0.41, 1.34) | 0.321 | 78 (0-90) | NA | 0.042 | NP | 0.06-9.36 |
| HOMA-IR (101) | Metabolic Diseases | Placebo/no treatment | 9 | 494 |  | SMD to OR | 0.15 (0.04, 0.51) | 0.002 | 93 (89-95) | NA | 0.162 | NP | 0.00-13.01 |
| HOMA-IR (102) | General | Placebo/no treatment | 9 | 435 |  | MD to OR | 0.22 (0.10, 0.51) | 4.06E-04 | 81 (62-88) | NA | 0.646 | NP | 0.01-3.37 |
| Insulin (101) | Metabolic Diseases | Placebo/no treatment | 8 | 463 |  | SMD to OR | 0.1(0.03,0.36) | 4.44E-04 | 91 (86-94) | NA | 0.008 | NP | 0.00-10.00 |
| Insulin (102) | General | Placebo/no treatment | 8 | 380 |  | MD to OR | 0.03(0,0.33) | 0.004 | 66(4-82) | NA | 0.084 | NP | 0.00-38.80 |
| **Other outcomes** | | | | | | | | | | | | | |
| Amputation (74) | Preexisting diseases | Placebo/no treatment | 2 | 2,294 | 116 | RR | NA | NA | NA | NA | NA | NA | NA |
| baseline hyperemic flow (87) | patients with coronary artery disease | Placebo/no treatment | 3 | 187 | NA | MD to OR | 6.29 (0, 2.40+05) | 0.733 | 0 (0-73) | 0.888 | 0.973 | NP | 0.00-3.05E+30 |
| Dental Hyperplasia index (98) | General | Placebo/no treatment | 2 | 28 | NA | MD to OR | 1.02 (0.65 ,1.60) | 0.939 | 0 | 0.721 | NA | NP | NA |
| dental Plaque index (98) | General | Placebo/no treatment | 2 | 28 | NA | MD to OR | 0.97 (0.43, 2.15) | 0.932 | 0 | 0.460 | NA | NP | NA |
| Gingival health index (98) | General | Placebo/no treatment | 2 | 28 | NA | MD to OR | 0.99 (0.79, 1.25) | 0.956 | 0 | 0.841 | NA | NP | NA |
| Liver toxicity (104) | patients receiving methotrexate | Placebo/no treatment | 2 | 302 | 65 | RR | 0.20 (0.11, 0.36) | 7.54E-08 | NA | NA | NA | NP | NA |
| Nausea / GI upset (104) | patients receiving methotrexate | Placebo/no treatment | 3 | 355 | 123 | RR | 0.77 (0.58, 1.03) | 0.074 | 0 (0-73) | 0.450 | 0.159 | NP | 0.12-4.95 |
| Neutropenia (104) | patients receiving methotrexate | Placebo/no treatment | 2 | 302 | 8 | RR | 1.68 (0.40, 6.98) | 0.477 | NA | NA | NA | NP | NA |
| plasma homocysteine (103) | type 2 diabetes | Placebo/no treatment | 4 | 183 | NA | MD to OR | 0 (0, 0.01) | 7.50E-14 | 44 (0-80) | 0.149 | 0.002 | 0.995 | 0.00-0.82 |
| plasma homocysteine (105) | childbearing age, pregnant and lactating women | Placebo/no treatment | 9 | 683 | NA | β to OR | 0.93 (0.89, 0.97) | 1.06E-03 | 72 (34-84) | <0.001 | 0.964 | NP | 0.81-1.06 |
| plasma homocysteine (107) | General | Placebo/no treatment | 25 | 3,001 | NA | OR | 0.79 (0.76, 0.81) | 2.34E-53 | 80 (72-85) | <0.001 | 0.042 | 0.986 | 0.68-0.91 |
| plasma homocysteine (87) | patients with coronary artery disease | Placebo/no treatment | 4 | 327 | NA | MD to OR | 0 (0, 0.03) | 5.45E-06 | 84 (62-91) | <0.001 | 0.024 | 0.994 | 0.00-18.85 |
| plasma homocysteine (65) | Preexisting diseases | Placebo/no treatment | 12 | 38,418 | NA | MD to OR | 0.01 (0, 0.01) | 7.88E-36 | 91 (87-94) |  | 0.211 | NP | 0-0.12 |
| red blood cell folate (105) | childbearing age, pregnant and lactating women | Placebo/no treatment | 9 | 692 | NA | β to OR | 1.47 (1.31, 1.66) | 9.83E-11 | 92 (87-94) | <0.001 | 0.883 | NP | 0.97-2.24 |
| red blood cell folate (108) | General | Placebo/no treatment | 7 | 1,441 | NA | OR | 1.32 (1.26, 1.39) | 1.05E-27 | 87 (78-91) | <0.001 | 0.028 | NP | 1.11-1.58 |
| Serum/plasma folate (105) | childbearing age, pregnant and lactating women | Placebo/no treatment | 9 | 692 | NA | β to OR | 1.47 (1.31, 1.65) | 1.01E-10 | 92 (88-94) | <0.001 | 0.666 | NP | 0.97-2.22 |
| Serum/plasma folate (108) | General | Placebo/no treatment | 17 | 2,294 | NA | OR | 1.55 (1.42, 1.70) | 2.83E-21 | 98 (0-100) | <0.001 | 0.956 | 0.987 | 0.96-2.50 |
| Stomatitis (104) | patients receiving methotrexate | Placebo/no treatment | 2 | 302 | 45 | RR | 0.89 (0.52, 1.52) | 0.675 | NA | NA | NA | NP | NA |
| Total withdrawals (104) | patients receiving methotrexate | Placebo/no treatment | 3 | 355 | 89 | RR | 0.44 (0.29, 0.65) | 5.38E-05 | 0 (0-73) | 0.781 | 0.025 | NP | 0.03-5.87 |

### Supplementary Table S8. AMSTAR 2 scores for different health outcomes in meta-analysis of RCT studies.

| **Outcomes** | **Population** | **Versus** | 1 | 2 | 3 | 4 | 5 | 6 | 7 | 8 | 9 | 10 | 11 | 12 | 13 | 14 | 15 | 16 | Rating |
| --- | --- | --- | --- | --- | --- | --- | --- | --- | --- | --- | --- | --- | --- | --- | --- | --- | --- | --- | --- |
| **All cause and cause specific mortality** | | | | | | | | | | | | | | | | | | | |
| All-cause mortality (66) | Preexisting diseases | Placebo/no treatment | Yes | No | No | PY | Yes | Yes | Yes | PY | No | No | Yes | No | Yes | Yes | Yes | Yes | CL |
| Cancer mortality (67) | Preexisting diseases | Placebo/no treatment | Yes | No | Yes | PY | Yes | Yes | Yes | PY | Yes | No | Yes | Yes | Yes | Yes | Yes | No | CL |
| **Birth outcomes** | | | | | | | | | | | | | | | | | | | |
| Anencephaly (68) | Pregnancy | Placebo/no treatment | Yes | Yes | No | PY | Yes | Yes | Yes | PY | Yes | No | Yes | Yes | Yes | Yes | No | No | Low |
| Birth weight (69) | Pregnancy | Placebo/no treatment | Yes | No | No | PY | Yes | Yes | Yes | PY | Yes | Yes | Yes | Yes | Yes | No | No | Yes | CL |
| Cleft lip (68) | Pregnancy | Placebo/no treatment | Yes | Yes | No | PY | Yes | Yes | Yes | PY | Yes | No | Yes | Yes | Yes | Yes | No | No | Low |
| Cleft palate (68) | Pregnancy | Placebo/no treatment | Yes | Yes | No | PY | Yes | Yes | Yes | PY | Yes | No | Yes | Yes | Yes | Yes | No | No | Low |
| Congenital cardiovascular anomalies (68) | Pregnancy | Placebo/no treatment | Yes | Yes | No | PY | Yes | Yes | Yes | PY | Yes | No | Yes | Yes | Yes | Yes | No | No | Low |
| Elective termination of pregnancy for fetal anomalies (68) | Pregnancy | Placebo/no treatment | Yes | Yes | No | PY | Yes | Yes | Yes | PY | Yes | No | Yes | Yes | Yes | Yes | No | No | Low |
| Low birthweight (70) | Pregnancy | Placebo/no treatment | Yes | Yes | No | PY | Yes | Yes | Yes | PY | Yes | No | Yes | Yes | Yes | Yes | Yes | Yes | Moderate |
| Megaloblastic anaemia (70) | Pregnancy | Placebo/no treatment | Yes | Yes | No | PY | Yes | Yes | Yes | PY | Yes | No | Yes | Yes | Yes | Yes | Yes | Yes | Moderate |
| Neural tube defects (68) | Pregnancy | Placebo/no treatment | Yes | Yes | No | PY | Yes | Yes | Yes | PY | Yes | No | Yes | Yes | Yes | Yes | No | No | Low |
| Neural tube disorders recurrent (15) | Previous pregnancy with NTD indicates | Placebo/no treatment | Yes | No | No | PY | No | No | Yes | PY | Yes | No | Yes | Yes | Yes | Yes | No | Yes | CL |
| Other congenital anomalies (68) | Pregnancy | Placebo/no treatment | Yes | Yes | No | PY | Yes | Yes | Yes | PY | Yes | No | Yes | Yes | Yes | Yes | No | No | Low |
| Perinatal death (71) | Pregnancy | Placebo/no treatment | Yes | Yes | No | PY | Yes | Yes | Yes | PY | Yes | No | Yes | Yes | Yes | Yes | Yes | Yes | Moderate |
| Placental weight (69) | Pregnancy | Placebo/no treatment | Yes | No | No | PY | Yes | Yes | Yes | PY | Yes | Yes | Yes | Yes | Yes | No | No | Yes | CL |
| Preterm birth (70) | Pregnancy | Placebo/no treatment | Yes | Yes | No | PY | Yes | Yes | Yes | PY | Yes | No | Yes | Yes | Yes | Yes | Yes | Yes | Moderate |
| Spina bifida (68) | Pregnancy | Placebo/no treatment | Yes | Yes | No | PY | Yes | Yes | Yes | PY | Yes | No | Yes | Yes | Yes | Yes | No | No | Low |
| Stillbirths (68) | Pregnancy | Placebo/no treatment | Yes | Yes | No | PY | Yes | Yes | Yes | PY | Yes | No | Yes | Yes | Yes | Yes | No | No | Low |
| Stillbirths/neonatal deaths (70) | Pregnancy | Placebo/no treatment | Yes | Yes | No | PY | Yes | Yes | Yes | PY | Yes | No | Yes | Yes | Yes | Yes | Yes | Yes | Moderate |
| **Cancer outcomes** | | | | | | | | | | | | | | | | | | | |
| Advanced colorectal lesion (76) | patients with an adenoma history | Placebo/no treatment | Yes | No | No | No | No | No | No | PY | No | No | Yes | No | No | Yes | No | No | CL |
| breast cancer (67) | Preexisting diseases | Placebo/no treatment | Yes | No | Yes | PY | Yes | Yes | Yes | PY | Yes | No | Yes | Yes | Yes | Yes | Yes | No | CL |
| cancer incidence (73) | Preexisting diseases | Placebo/no treatment | Yes | No | No | No | No | No | No | PY | No | No | Yes | No | No | Yes | No | Yes | CL |
| Colorectal adenoma (73) | Preexisting diseases | Placebo/no treatment | Yes | No | No | No | No | No | No | PY | No | No | Yes | No | No | Yes | No | Yes | CL |
| colorectal adenoma recurrence (77) | General | Placebo/no treatment | Yes | No | No | PY | No | No | No | PY | Yes | No | Yes | Yes | Yes | Yes | Yes | No | CL |
| colorectal adenomatous lesion (79) (folate supplementation up to 3 years) | General | Placebo/no treatment | Yes | No | No | PY | Yes | Yes | Yes | PY | Yes | No | Yes | Yes | Yes | Yes | No | Yes | CL |
| colorectal adenomatous lesion (79) (folate supplementation for over 3 years) | General | Placebo/no treatment | Yes | No | No | PY | Yes | Yes | Yes | PY | Yes | No | Yes | Yes | Yes | Yes | No | Yes | CL |
| colorectal cancer (80) | General | Placebo/no treatment | Yes | No | No | PY | Yes | Yes | Yes | PY | Yes | No | Yes | Yes | Yes | Yes | Yes | Yes | Low |
| colorectal cancer (32) | Preexisting diseases | Placebo/no treatment | Yes | No | Yes | PY | No | No | Yes | PY | Yes | No | Yes | Yes | Yes | Yes | Yes | Yes | CL |
| Hematological cancers (47) | Preexisting diseases | Placebo/no treatment | Yes | Yes | No | PY | Yes | Yes | Yes | PY | Yes | No | Yes | Yes | Yes | Yes | Yes | Yes | Moderate |
| hematological malignancy (67) | Preexisting diseases | Placebo/no treatment | Yes | No | Yes | PY | Yes | Yes | Yes | PY | Yes | No | Yes | Yes | Yes | Yes | Yes | No | CL |
| Lung Cancer (47) | Preexisting diseases | Placebo/no treatment | Yes | Yes | No | PY | Yes | Yes | Yes | PY | Yes | No | Yes | Yes | Yes | Yes | Yes | Yes | Moderate |
| Melanoma (67) | Preexisting diseases | Placebo/no treatment | Yes | No | Yes | PY | Yes | Yes | Yes | PY | Yes | No | Yes | Yes | Yes | Yes | Yes | No | CL |
| prostate cancer (47) | Preexisting diseases | Placebo/no treatment | Yes | Yes | No | PY | Yes | Yes | Yes | PY | Yes | No | Yes | Yes | Yes | Yes | Yes | Yes | Moderate |
| **Cardiovascular outcomes** | | | | | | | | | | | | | | | | | | | |
| Angina (81) | Preexisting diseases | Placebo/no treatment | Yes | No | No | PY | Yes | Yes | Yes | PY | Yes | Yes | Yes | Yes | Yes | Yes | Yes | Yes | Low |
| cardiovascular disease (82) | Preexisting diseases | Placebo/no treatment | Yes | No | No | PY | Yes | Yes | Yes | PY | Yes | No | Yes | Yes | Yes | Yes | Yes | Yes | Low |
| cardiovascular events (81) | Preexisting diseases | Placebo/no treatment | Yes | No | No | PY | Yes | Yes | Yes | PY | Yes | Yes | Yes | Yes | Yes | Yes | Yes | Yes | Low |
| Carotid intima-media thickness (84) | Preexisting diseases | Placebo/no treatment | Yes | No | No | No | Yes | Yes | Yes | PY | Yes | No | Yes | Yes | Yes | Yes | No | Yes | CL |
| Coronary syndrome (74) | Preexisting diseases | Placebo/no treatment | Yes | No | No | PY | Yes | Yes | Yes | PY | Yes | No | Yes | Yes | Yes | Yes | No | Yes | CL |
| coronary artery bypass grafting (85) | General | Placebo/no treatment | Yes | No | Yes | No | Yes | Yes | Yes | PY | Yes | No | Yes | Yes | Yes | Yes | Yes | Yes | CL |
| coronary heart disease (82) | Preexisting diseases | Placebo/no treatment | Yes | No | No | PY | Yes | Yes | Yes | PY | Yes | No | Yes | Yes | Yes | Yes | Yes | Yes | Low |
| coronary restenosis (85) | General | Placebo/no treatment | Yes | No | Yes | No | Yes | Yes | Yes | PY | Yes | No | Yes | Yes | Yes | Yes | Yes | Yes | CL |
| coronary revascularization (85) | General | Placebo/no treatment | Yes | No | Yes | No | Yes | Yes | Yes | PY | Yes | No | Yes | Yes | Yes | Yes | Yes | Yes | CL |
| diastolic blood pressure (86) | hypertensive patients | Placebo/no treatment | Yes | No | No | No | No | No | Yes | PY | No | No | Yes | No | No | No | Yes | No | CL |
| diastolic blood pressure (87) | patients with coronary artery disease | Placebo/no treatment | Yes | No | No | No | Yes | Yes | Yes | PY | Yes | No | Yes | Yes | Yes | Yes | Yes | No | CL |
| diastolic blood pressure (88) | patients with metabolic diseases | Placebo/no treatment | Yes | No | No | PY | No | No | Yes | PY | Yes | No | Yes | Yes | Yes | Yes | Yes | Yes | CL |
| end diastolic diameter (87) | patients with coronary artery disease | Placebo/no treatment | Yes | No | No | No | Yes | Yes | Yes | PY | Yes | No | Yes | Yes | Yes | Yes | Yes | No | CL |
| HDL-cholesterol (88) | patients with metabolic diseases | Placebo/no treatment | Yes | No | No | PY | No | No | Yes | PY | Yes | No | Yes | Yes | Yes | Yes | Yes | Yes | CL |
| heart rate (87) | patients with coronary artery disease | Placebo/no treatment | Yes | No | No | No | Yes | Yes | Yes | PY | Yes | No | Yes | Yes | Yes | Yes | Yes | No | CL |
| LDL-cholesterol (88) | patients with metabolic diseases | Placebo/no treatment | Yes | No | No | PY | No | No | Yes | PY | Yes | No | Yes | Yes | Yes | Yes | Yes | Yes | CL |
| Myocardial infarction (74) | Preexisting diseases | Placebo/no treatment | Yes | No | No | PY | Yes | Yes | Yes | PY | Yes | No | Yes | Yes | Yes | Yes | No | Yes | CL |
| Percutaneous coronary intervention (85) | General | Placebo/no treatment | Yes | No | Yes | No | Yes | Yes | Yes | PY | Yes | No | Yes | Yes | Yes | Yes | Yes | Yes | CL |
| Primary cardiovascular clinical end point (65) | Preexisting diseases | Placebo/no treatment | Yes | No | No | No | Yes | Yes | Yes | PY | No | No | Yes | No | No | Yes | Yes | No | CL |
| Revascularization (85) | General | Placebo/no treatment | Yes | No | Yes | No | Yes | Yes | Yes | PY | Yes | No | Yes | Yes | Yes | Yes | Yes | Yes | CL |
| Revascularization (74) | Preexisting diseases | Placebo/no treatment | Yes | No | No | PY | Yes | Yes | Yes | PY | Yes | No | Yes | Yes | Yes | Yes | No | Yes | CL |
| Stroke (93) | Preexisting diseases | Placebo/no treatment | Yes | No | No | No | Yes | Yes | Yes | PY | Yes | No | Yes | Yes | Yes | Yes | Yes | No | CL |
| systolic blood pressure (86) | hypertensive patients | Placebo/no treatment | Yes | No | No | No | No | No | Yes | PY | No | No | Yes | No | No | No | Yes | No | CL |
| systolic blood pressure (87) | patients with coronary artery disease | Placebo/no treatment | Yes | No | No | No | Yes | Yes | Yes | PY | Yes | No | Yes | Yes | Yes | Yes | Yes | No | CL |
| systolic blood pressure (88) | patients with metabolic diseases | Placebo/no treatment | Yes | No | No | PY | No | No | Yes | PY | Yes | No | Yes | Yes | Yes | Yes | Yes | Yes | CL |
| total cholesterol (88) | patients with metabolic diseases | Placebo/no treatment | Yes | No | No | PY | No | No | Yes | PY | Yes | No | Yes | Yes | Yes | Yes | Yes | Yes | CL |
| Transient ischemic attack (81) | Preexisting diseases | Placebo/no treatment | Yes | No | No | PY | Yes | Yes | Yes | PY | Yes | Yes | Yes | Yes | Yes | Yes | Yes | Yes | Low |
| Triglycerides (88) | patients with metabolic diseases | Placebo/no treatment | Yes | No | No | PY | No | No | Yes | PY | Yes | No | Yes | Yes | Yes | Yes | Yes | Yes | CL |
| VLDL-cholesterol (88) | patients with metabolic diseases | Placebo/no treatment | Yes | No | No | PY | No | No | Yes | PY | Yes | No | Yes | Yes | Yes | Yes | Yes | Yes | CL |
| **Endothelial function** | | | | | | | | | | | | | | | | | | | |
| flow‑mediated dilation (87) | patients with coronary artery disease | Placebo/no treatment | Yes | No | No | No | Yes | Yes | Yes | PY | Yes | No | Yes | Yes | Yes | Yes | Yes | No | CL |
| flow-mediated dilation (86) | hypertensive patients | Placebo/no treatment | Yes | No | No | No | No | No | Yes | PY | No | No | Yes | No | No | No | Yes | No | CL |
| Flow-mediated dilatation (95) | General | Placebo/no treatment | Yes | No | No | No | Yes | Yes | Yes | PY | Yes | No | Yes | Yes | Yes | Yes | Yes | No | CL |
| glyceryl‑trinitrate (GTN) diameter change (87) | patients with coronary artery disease | Placebo/no treatment | Yes | No | No | No | Yes | Yes | Yes | PY | Yes | No | Yes | Yes | Yes | Yes | Yes | No | CL |
| peak hyperemic flow (87) | patients with coronary artery disease | Placebo/no treatment | Yes | No | No | No | Yes | Yes | Yes | PY | Yes | No | Yes | Yes | Yes | Yes | Yes | No | CL |
| **Neurocognitive disorders** | | | | | | | | | | | | | | | | | | | |
| cognitive function test scores (96) | General | Placebo/no treatment | Yes | No | No | PY | Yes | Yes | No | PY | No | No | Yes | No | No | Yes | Yes | Yes | CL |
| endpoint scores of the respective rating scales (97) | depressive disorder | Placebo/no treatment | Yes | No | No | PY | No | No | Yes | PY | Yes | No | Yes | Yes | Yes | Yes | Yes | Yes | Low |
| epilepsy seizure frequency (98) | General | Placebo/no treatment | Yes | Yes | No | PY | Yes | Yes | Yes | PY | No | No | Yes | No | Yes | Yes | Yes | Yes | Low |
| Hamilton Depression Rating Scale score (99) | depressive disorder | Placebo/no treatment | Yes | No | No | PY | Yes | Yes | Yes | Yes | No | No | Yes | No | No | Yes | Yes | No | CL |
| BDI: Beck Depression Inventory/ HAMD: Hamilton Depression Scale (100) | patients with unipolar depressive illness | Placebo/no treatment | Yes | No | No | PY | Yes | Yes | No | PY | Yes | No | Yes | Yes | Yes | Yes | No | Yes | CL |
| **Pregnancy outcomes** | | | | | | | | | | | | | | | | | | | |
| gestational hypertension/preeclampsia (61) | Pregnancy | Placebo/no treatment | Yes | No | No | PY | Yes | Yes | Yes | PY | Yes | No | Yes | Yes | Yes | Yes | Yes | Yes | Low |
| Length of gestation (69) | Pregnancy | Placebo/no treatment | Yes | No | No | PY | Yes | Yes | Yes | PY | Yes | Yes | Yes | Yes | Yes | No | No | Yes | CL |
| Low pre-delivery serum folate (70) | Pregnancy | Placebo/no treatment | Yes | Yes | No | PY | Yes | Yes | Yes | PY | Yes | No | Yes | Yes | Yes | Yes | Yes | Yes | Moderate |
| Miscarriage (68) | Pregnancy | Placebo/no treatment | Yes | Yes | No | PY | Yes | Yes | Yes | PY | Yes | No | Yes | Yes | Yes | Yes | No | No | Low |
| Multiple pregnancy (68) | Pregnancy | Placebo/no treatment | Yes | Yes | No | PY | Yes | Yes | Yes | PY | Yes | No | Yes | Yes | Yes | Yes | No | No | Low |
| Pre-delivery anaemia (70) | Pregnancy | Placebo/no treatment | Yes | Yes | No | PY | Yes | Yes | Yes | PY | Yes | No | Yes | Yes | Yes | Yes | Yes | Yes | Moderate |
| pre-delivery hemoglobin level (70) | Pregnancy | Placebo/no treatment | Yes | Yes | No | PY | Yes | Yes | Yes | PY | Yes | No | Yes | Yes | Yes | Yes | Yes | Yes | Moderate |
| pre-delivery serum folate (70) | Pregnancy | Placebo/no treatment | Yes | Yes | No | PY | Yes | Yes | Yes | PY | Yes | No | Yes | Yes | Yes | Yes | Yes | Yes | Moderate |
| **Diabetes related outcomes** | | | | | | | | | | | | | |  |  |  |  |  |  |
| Fasting Glucose (101) | Metabolic Diseases | Placebo/no treatment | Yes | No | No | PY | Yes | Yes | Yes | PY | Yes | No | Yes | Yes | Yes | Yes | Yes | Yes | Low |
| Fasting Glucose (102) | General | Placebo/no treatment | Yes | No | No | PY | Yes | Yes | Yes | PY | Yes | Yes | Yes | Yes | Yes | Yes | Yes | No | Low |
| HbA1c (103) | type 2 diabetes | Placebo/no treatment | Yes | No | No | PY | Yes | Yes | Yes | PY | Yes | No | Yes | Yes | Yes | Yes | Yes | Yes | Low |
| HbA1c (101) | Metabolic Diseases | Placebo/no treatment | Yes | No | No | PY | Yes | Yes | Yes | PY | Yes | No | Yes | Yes | Yes | Yes | Yes | Yes | Low |
| HbA1c (102) | General | Placebo/no treatment | Yes | No | No | PY | Yes | Yes | Yes | PY | Yes | Yes | Yes | Yes | Yes | Yes | Yes | No | Low |
| HOMA-IR (101) | Metabolic Diseases | Placebo/no treatment | Yes | No | No | PY | Yes | Yes | Yes | PY | Yes | No | Yes | Yes | Yes | Yes | Yes | Yes | Low |
| HOMA-IR (102) | General | Placebo/no treatment | Yes | No | No | PY | Yes | Yes | Yes | PY | Yes | Yes | Yes | Yes | Yes | Yes | Yes | No | Low |
| Insulin (101) | Metabolic Diseases | Placebo/no treatment | Yes | No | No | PY | Yes | Yes | Yes | PY | Yes | No | Yes | Yes | Yes | Yes | Yes | Yes | Low |
| Insulin (102) | General | Placebo/no treatment | Yes | No | No | PY | Yes | Yes | Yes | PY | Yes | Yes | Yes | Yes | Yes | Yes | Yes | No | Low |
| **Other outcomes** | | | | | | | | | | | | | |  |  |  |  |  |  |
| Amputation (74) | Preexisting diseases | Placebo/no treatment | Yes | No | No | PY | Yes | Yes | Yes | PY | Yes | No | Yes | Yes | Yes | Yes | No | Yes | CL |
| baseline hyperemic flow (87) | patients with coronary artery disease | Placebo/no treatment | Yes | No | No | No | Yes | Yes | Yes | PY | Yes | No | Yes | Yes | Yes | Yes | Yes | No | CL |
| Dental Hyperplasia index(98) | General | Placebo/no treatment | Yes | Yes | No | PY | Yes | Yes | Yes | PY | No | No | Yes | No | Yes | Yes | Yes | Yes | Low |
| dental Plaque index (98) | General | Placebo/no treatment | Yes | Yes | No | PY | Yes | Yes | Yes | PY | No | No | Yes | No | Yes | Yes | Yes | Yes | Low |
| Gingival health index (98) | General | Placebo/no treatment | Yes | Yes | No | PY | Yes | Yes | Yes | PY | No | No | Yes | No | Yes | Yes | Yes | Yes | Low |
| Liver toxicity (104) | patients receiving methotrexate | Placebo/no treatment | Yes | No | No | PY | Yes | Yes | Yes | PY | Yes | No | Yes | Yes | Yes | Yes | No | Yes | CL |
| Nausea / GI upset (104) | patients receiving methotrexate | Placebo/no treatment | Yes | No | No | PY | Yes | Yes | Yes | PY | Yes | No | Yes | Yes | Yes | Yes | No | Yes | CL |
| Neutropenia (104) | patients receiving methotrexate | Placebo/no treatment | Yes | No | No | PY | Yes | Yes | Yes | PY | Yes | No | Yes | Yes | Yes | Yes | No | Yes | CL |
| plasma homocysteine (103) | type 2 diabetes | Placebo/no treatment | Yes | No | No | PY | Yes | Yes | Yes | PY | Yes | No | Yes | Yes | Yes | Yes | Yes | Yes | Low |
| plasma homocysteine (105) | childbearing age, pregnant and lactating women | Placebo/no treatment | Yes | Yes | No | PY | Yes | Yes | Yes | PY | Yes | Yes | Yes | Yes | Yes | Yes | No | Yes | Low |
| plasma homocysteine (107) | General | Placebo/no treatment | Yes | No | No | No | No | Yes | No | PY | No | No | Yes | No | No | Yes | No | Yes | CL |
| plasma homocysteine (87) | patients with coronary artery disease | Placebo/no treatment | Yes | No | No | No | Yes | Yes | Yes | PY | Yes | No | Yes | Yes | Yes | Yes | Yes | No | CL |
| plasma homocysteine (65) | Preexisting diseases | Placebo/no treatment | Yes | No | No | No | Yes | Yes | Yes | PY | No | No | Yes | No | No | Yes | Yes | No | CL |
| red blood cell folate (105) | childbearing age, pregnant and lactating women | Placebo/no treatment | Yes | Yes | No | PY | Yes | Yes | Yes | PY | Yes | Yes | Yes | Yes | Yes | Yes | No | Yes | Low |
| red blood cell folate (108) | General | Placebo/no treatment | Yes | Yes | No | PY | Yes | Yes | Yes | PY | Yes | Yes | Yes | Yes | Yes | Yes | Yes | Yes | Moderate |
| Serum/plasma folate (105) | childbearing age, pregnant and lactating women | Placebo/no treatment | Yes | Yes | No | PY | Yes | Yes | Yes | PY | Yes | Yes | Yes | Yes | Yes | Yes | No | Yes | Low |
| Serum/plasma folate (108) | General | Placebo/no treatment | Yes | Yes | No | PY | Yes | Yes | Yes | PY | Yes | Yes | Yes | Yes | Yes | Yes | Yes | Yes | Moderate |
| Stomatitis (104) | patients receiving methotrexate | Placebo/no treatment | Yes | No | No | PY | Yes | Yes | Yes | PY | Yes | No | Yes | Yes | Yes | Yes | No | Yes | CL |
| Total withdrawals (104) | patients receiving methotrexate | Placebo/no treatment | Yes | No | No | PY | Yes | Yes | Yes | PY | Yes | No | Yes | Yes | Yes | Yes | No | Yes | CL |

PY=partial yes; CL=critically low; NRSI= non-randomized studies

### Supplementary Table S9. Health outcomes and GRADE classification in meta-analysis of RCT studies.

| **Outcomes** | **Population** | **Versus** | **N studies** | **Risk of Bias** | **Inconsistency** | **Indirectness** | **Imprecision** | **Publication bias** | **Large effect** | **Plausible Confounding** | **Dose-response** | **Quality** |
| --- | --- | --- | --- | --- | --- | --- | --- | --- | --- | --- | --- | --- |
| **All cause and cause specific mortality** | | | | | | | | | | | | |
| All-cause mortality (66) | Preexisting diseases | Placebo/no treatment | 24 | Serious risk | No serious inconsistency | No Serious Indirectness | No serious imprecision | Undetected | No | Would not reduce effect | No | ⊕⊕⊕〇 Moderate |
| Cancer mortality (67) | Preexisting diseases | Placebo/no treatment | 6 | Serious risk | No serious inconsistency | No Serious Indirectness | No serious imprecision | Undetected | No | would not reduce effect | No | ⊕⊕⊕〇 Moderate |
| **Birth outcomes** | | | | | | | | | | | | |
| Anencephaly (68) | Pregnancy | Placebo/no treatment | 4 | Serious risk | No serious inconsistency | No Serious Indirectness | Serious imprecision | Undetected | No | Would not reduce effect | No | ⊕⊕〇〇 Low |
| Birth weight (69) | Pregnancy | Placebo/no treatment | 9 | Serious risk | Serious inconsistency | No Serious Indirectness | No serious imprecision | NA | No | Would reduce effect | No | ⊕⊕⊕〇 Moderate |
| Cleft lip (68) | Pregnancy | Placebo/no treatment | 3 | Serious risk | No serious inconsistency | No Serious Indirectness | Very serious imprecision | Undetected | No | Would not reduce effect | No | ⊕〇〇〇 Very Low |
| Cleft palate (68) | Pregnancy | Placebo/no treatment | 3 | Serious risk | Serious inconsistency | No Serious Indirectness | Very serious imprecision | Undetected | No | Would not reduce effect | No | ⊕〇〇〇 Very Low |
| Congenital cardiovascular anomalies (68) | Pregnancy | Placebo/no treatment | 3 | Serious risk | No serious inconsistency | No Serious Indirectness | Serious imprecision | Undetected | No | Would not reduce effect | No | ⊕⊕〇〇 Low |
| Elective termination of pregnancy for fetal anomalies (68) | Pregnancy | Placebo/no treatment | 4 | Serious risk | No serious inconsistency | No Serious Indirectness | No serious imprecision | Undetected | Large | Would reduce effect | No | ⊕⊕⊕⊕High |
| Low birthweight (70) | Pregnancy | Placebo/no treatment | 4 | Serious risk | No serious inconsistency | No Serious Indirectness | No serious imprecision | Undetected | No | would not reduce effect | No | ⊕⊕⊕〇 Moderate |
| Megaloblastic anaemia (70) | Pregnancy | Placebo/no treatment | 4 | Serious risk | No serious inconsistency | No Serious Indirectness | No serious imprecision | Undetected | Large | would reduce effect | No | ⊕⊕⊕⊕High |
| Neural tube defects (68) | Pregnancy | Placebo/no treatment | 5 | Serious risk | No serious inconsistency | No Serious Indirectness | No serious imprecision | Undetected | Large | Would reduce effect | No | ⊕⊕⊕⊕High |
| Neural tube disorders recurrent (15) | Previous pregnancy with NTD indicates | Placebo/no treatment | 3 | Serious risk | No serious inconsistency | No Serious Indirectness | No serious imprecision | Undetected | Large | would reduce effect | No | ⊕〇〇〇 Very Low |
| Other congenital anomalies (68) | Pregnancy | Placebo/no treatment | 3 | Serious risk | Serious inconsistency | No Serious Indirectness | Serious imprecision | Undetected | No | Would not reduce effect | No | ⊕〇〇〇 Very Low |
| Perinatal death (71) | Pregnancy | Placebo/no treatment | 2 | Serious risk | No serious inconsistency | No Serious Indirectness | Serious imprecision | Undetected | No | would not reduce effect | No | ⊕⊕〇〇 Low |
| Placental weight (69) | Pregnancy | Placebo/no treatment | 5 | Serious risk | Serious inconsistency | No Serious Indirectness | No serious imprecision | NA | No | Would not reduce effect | No | ⊕⊕〇〇 Low |
| Preterm birth (70) | Pregnancy | Placebo/no treatment | 3 | Serious risk | No serious inconsistency | No Serious Indirectness | No serious imprecision | Undetected | No | would not reduce effect | No | ⊕⊕⊕〇 Moderate |
| Spina bifida (68) | Pregnancy | Placebo/no treatment | 3 | Serious risk | No serious inconsistency | No Serious Indirectness | Serious imprecision | Undetected | No | Would not reduce effect | No | ⊕〇〇〇 Very Low |
| Stillbirths (68) | Pregnancy | Placebo/no treatment | 4 | Serious risk | No serious inconsistency | No Serious Indirectness | Serious imprecision | Undetected | No | Would not reduce effect | No | ⊕⊕〇〇 Low |
| Stillbirths/neonatal deaths (70) | Pregnancy | Placebo/no treatment | 3 | Serious risk | No serious inconsistency | No Serious Indirectness | No serious imprecision | Undetected | No | would not reduce effect | No | ⊕⊕⊕〇 Moderate |
| **Cancer outcomes** | | | | | | | | | | | | |
| Advanced colorectal lesion (76) | patients with an adenoma history | Placebo/no treatment | 3 | Serious risk | No serious inconsistency | No Serious Indirectness | No serious imprecision | a | No | would not reduce effect | No | ⊕〇〇〇 Very Low |
| breast cancer (67) | Preexisting diseases | Placebo/no treatment | 4 | Serious risk | No serious inconsistency | No Serious Indirectness | No serious imprecision | Undetected | No | would not reduce effect | No | ⊕〇〇〇 Very Low |
| cancer incidence (73) | Preexisting diseases | Placebo/no treatment | 13 | Serious risk | No serious inconsistency | No Serious Indirectness | No serious imprecision | NA | No | Would not reduce effect | No | ⊕⊕⊕〇 Moderate |
| Colorectal adenoma (73) | Preexisting diseases | Placebo/no treatment | 3 | Serious risk | No serious inconsistency | No Serious Indirectness | No serious imprecision | NA | No | Would not reduce effect | No | ⊕〇〇〇 Very Low |
| colorectal adenoma recurrence (77) | General | Placebo/no treatment | 4 | Serious risk | Serious inconsistency | No Serious Indirectness | No serious imprecision | Undetected | No | would not reduce effect | No | ⊕⊕〇〇 Low |
| colorectal adenomatous lesion (79) (folate supplementation upto 3 years) | General | Placebo/no treatment | 2 | Serious risk | No serious inconsistency | No Serious Indirectness | No serious imprecision | NA | No | would not reduce effect | No | ⊕⊕⊕〇 Moderate |
| colorectal adenomatous lesion (79) (folate supplementation for over 3 years) | General | Placebo/no treatment | 2 | Serious risk | No serious inconsistency | No Serious Indirectness | No serious imprecision | NA | No | would reduce effect | No | ⊕〇〇〇 Very Low |
| colorectal cancer (80) | General | Placebo/no treatment | 8 | Serious risk | No serious inconsistency | No Serious Indirectness | No serious imprecision | Undetected | No | would not reduce effect | No | ⊕⊕⊕〇 Moderate |
| colorectal cancer (32) | Preexisting diseases | Placebo/no treatment | 11 | Serious risk | Serious inconsistency | No Serious Indirectness | No serious imprecision | Undetected | No | would not reduce effect | No | ⊕〇〇〇 Very Low |
| Hematological cancers (47) | Preexisting diseases | Placebo/no treatment | 3 | Serious risk | NA | No Serious Indirectness | Serious imprecision | NA | No | Would not reduce effect | No | ⊕⊕〇〇 Low |
| hematological malignancy (67) | Preexisting diseases | Placebo/no treatment | 3 | Serious risk | No serious inconsistency | No Serious Indirectness | No serious imprecision | Undetected | No | would not reduce effect | No | ⊕〇〇〇 Very Low |
| Lung Cancer (47) | Preexisting diseases | Placebo/no treatment | 5 | Serious risk | NA | No Serious Indirectness | Serious imprecision | NA | No | Would not reduce effect | No | ⊕⊕〇〇 Low |
| Melanoma (67) | Preexisting diseases | Placebo/no treatment | 3 | Serious risk | No serious inconsistency | No Serious Indirectness | No serious imprecision | Undetected | No | would reduce effect | No | ⊕〇〇〇 Very Low |
| prostate cancer (47) | Preexisting diseases | Placebo/no treatment | 6 | Serious risk | No serious inconsistency | No Serious Indirectness | No serious imprecision | Undetected | No | Would reduce effect | No | ⊕⊕⊕⊕High |
| **Cardiovascular outcomes** | | | | | | | | | | | | |
| Angina (81) | Preexisting diseases | Placebo/no treatment | 4 | Serious risk | Serious inconsistency | No Serious Indirectness | No serious imprecision | Undetected | No | Would not reduce effect | No | ⊕⊕〇〇 Low |
| cardiovascular disease (82) | Preexisting diseases | Placebo/no treatment | 22 | Serious risk | Serious inconsistency | No Serious Indirectness | No serious imprecision | Undetected | No | would reduce effect | No | ⊕⊕⊕〇 Moderate |
| cardiovascular events (81) | Preexisting diseases | Placebo/no treatment | 21 | Serious risk | Serious inconsistency | No Serious Indirectness | No serious imprecision | Undetected | No | Would not reduce effect | No | ⊕⊕〇〇 Low |
| Carotid intima-media thickness (84) | Preexisting diseases | Placebo/no treatment | 10 | Serious risk | Very serious inconsistency | No Serious Indirectness | No serious imprecision | Likely | No | Would reduce effect | No | ⊕〇〇〇 Very Low |
| Coronary syndrome (74) | Preexisting diseases | Placebo/no treatment | 5 | Serious risk | No serious inconsistency | No Serious Indirectness | No serious imprecision | NA | No | Would not reduce effect | No | ⊕〇〇〇 Very Low |
| coronary artery bypass grafting (85) | General | Placebo/no treatment | 5 | Serious risk | No serious inconsistency | No Serious Indirectness | No serious imprecision | Undetected | No | would not reduce effect | No | ⊕⊕⊕〇 Moderate |
| coronary heart disease (82) | Preexisting diseases | Placebo/no treatment | 25 | Serious risk | No serious inconsistency | No Serious Indirectness | No serious imprecision | Undetected | No | would not reduce effect | No | ⊕〇〇〇 Very Low |
| coronary restenosis (85) | General | Placebo/no treatment | 3 | Serious risk | Very serious inconsistency | No Serious Indirectness | Serious imprecision | Undetected | No | would not reduce effect | No | ⊕〇〇〇 Very Low |
| coronary revascularization (85) | General | Placebo/no treatment | 9 | Serious risk | Serious inconsistency | No Serious Indirectness | No serious imprecision | Undetected | No | would not reduce effect | No | ⊕〇〇〇 Very Low |
| diastolic blood pressure (86) | hypertensive patients | Placebo/no treatment | 8 | Serious risk | Serious inconsistency | No Serious Indirectness | No serious imprecision | Undetected | No | would not reduce effect |  | ⊕⊕〇〇 Low |
| diastolic blood pressure (87) | patients with coronary artery disease | Placebo/no treatment | 4 | Serious risk | No serious inconsistency | No Serious Indirectness | No serious imprecision | Undetected | No | Would not reduce effect | No | ⊕⊕⊕〇 Moderate |
| diastolic blood pressure (88) | patients with metabolic diseases | Placebo/no treatment | 6 | Serious risk | Very serious inconsistency | No Serious Indirectness | No serious imprecision | Undetected | No | would not reduce effect | No | ⊕〇〇〇 Very Low |
| end diastolic diameter (87) | patients with coronary artery disease | Placebo/no treatment | 4 | Serious risk | No serious inconsistency | No Serious Indirectness | No serious imprecision | Undetected | No | Would not reduce effect | No | ⊕⊕⊕〇 Moderate |
| HDL-cholesterol (88) | patients with metabolic diseases | Placebo/no treatment | 10 | Serious risk | Very serious inconsistency | No Serious Indirectness | No serious imprecision | Undetected | No | would not reduce effect | No | ⊕〇〇〇 Very Low |
| heart rate (87) | patients with coronary artery disease | Placebo/no treatment | 4 | Serious risk | No serious inconsistency | No Serious Indirectness | Very serious imprecision | Undetected | No | Would not reduce effect | No | ⊕⊕〇〇 Low |
| LDL-cholesterol (88) | patients with metabolic diseases | Placebo/no treatment | 9 | Serious risk | Very serious inconsistency | No Serious Indirectness | No serious imprecision | Undetected | No | would not reduce effect | No | ⊕〇〇〇 Very Low |
| Myocardial infarction (74) | Preexisting diseases | Placebo/no treatment | 11 | Serious risk | No serious inconsistency | No Serious Indirectness | No serious imprecision | NA | No | Would not reduce effect | No | ⊕⊕⊕〇 Moderate |
| Percutaneous coronary intervention (85) | General | Placebo/no treatment | 5 | Serious risk | No serious inconsistency | No Serious Indirectness | No serious imprecision | Undetected | No | would not reduce effect | No | ⊕〇〇〇 Very Low |
| Primary cardiovascular clinical end point (65) | Preexisting diseases | Placebo/no treatment | 14 | Serious risk | Serious inconsistency | No Serious Indirectness | No serious imprecision | Undetected | No | would not reduce effect | No | ⊕〇〇〇 Very Low |
| Revascularization (85) | General | Placebo/no treatment | 7 | Serious risk | No serious inconsistency | No Serious Indirectness | No serious imprecision | Undetected | No | would not reduce effect | No | ⊕〇〇〇 Very Low |
| Revascularization (74) | Preexisting diseases | Placebo/no treatment | 10 | Serious risk | Serious inconsistency | No Serious Indirectness | No serious imprecision | NA | No | Would not reduce effect | No | ⊕⊕〇〇 Low |
| Stroke (93) | Preexisting diseases | Placebo/no treatment | 22 | Serious risk | No serious inconsistency | No Serious Indirectness | No serious imprecision | Undetected | No | would reduce effect | No | ⊕〇〇〇 Very Low |
| systolic blood pressure (86) | hypertensive patients | Placebo/no treatment | 8 | Serious risk | Serious inconsistency | No Serious Indirectness | No serious imprecision | Undetected | No | Would not reduce effect |  | ⊕〇〇〇 Very Low |
| systolic blood pressure (87) | patients with coronary artery disease | Placebo/no treatment | 4 | Serious risk | No serious inconsistency | No Serious Indirectness | Very serious imprecision | Undetected | No | Would not reduce effect | No | ⊕〇〇〇 Very Low |
| systolic blood pressure (88) | patients with metabolic diseases | Placebo/no treatment | 6 | Serious risk | Very serious inconsistency | No Serious Indirectness | No serious imprecision | Undetected | No | would not reduce effect | No | ⊕〇〇〇 Very Low |
| total cholesterol (88) | patients with metabolic diseases | Placebo/no treatment | 10 | Serious risk | Serious inconsistency | No Serious Indirectness | No serious imprecision | Undetected | No | would not reduce effect | No | ⊕〇〇〇 Very Low |
| Transient ischemic attack (81) | Preexisting diseases | Placebo/no treatment | 2 | Serious risk | No serious inconsistency | No Serious Indirectness | No serious imprecision | Undetected | No | Would not reduce effect | No | ⊕⊕⊕〇 Moderate |
| Triglycerides (88) | patients with metabolic diseases | Placebo/no treatment | 11 | Serious risk | Very serious inconsistency | No Serious Indirectness | No serious imprecision | Undetected | No | would not reduce effect | No | ⊕〇〇〇 Very Low |
| VLDL-cholesterol (88) | patients with metabolic diseases | Placebo/no treatment | 3 | Serious risk | No serious inconsistency | No Serious Indirectness | No serious imprecision | Undetected | No | would not reduce effect | No | ⊕〇〇〇 Very Low |
| **Endothelial function** | | | | | | | | | | | | |
| flow‑mediated dilation (87) | patients with coronary artery disease | Placebo/no treatment | 4 | Serious risk | No serious inconsistency | No Serious Indirectness | No serious imprecision | Undetected | No | Would reduce effect | No | ⊕⊕⊕⊕High |
| flow-mediated dilation (86) | hypertensive patients | Placebo/no treatment | 8 | Serious risk | Serious inconsistency | No Serious Indirectness | No serious imprecision | Undetected | No | would reduce effect |  | ⊕〇〇〇 Very Low |
| Flow-mediated dilatation (95) | General | Placebo/no treatment | 14 | Serious risk | Serious inconsistency | No Serious Indirectness | No serious imprecision | Undetected | No | would not reduce effect | No | ⊕〇〇〇 Very Low |
| glyceryl‑trinitrate (GTN) diameter change (87) | patients with coronary artery disease | Placebo/no treatment | 3 | Serious risk | No serious inconsistency | No Serious Indirectness | Very serious imprecision | Undetected | No | Would not reduce effect | No | ⊕〇〇〇 Very Low |
| peak hyperemic flow (87) | patients with coronary artery disease | Placebo/no treatment | 3 | Serious risk | No serious inconsistency | No Serious Indirectness | Very serious imprecision | Undetected | No | Would not reduce effect | No | ⊕〇〇〇 Very Low |
| **Neurocognitive disorders** | | | | | | | | | | | | |
| cognitive function test scores (96) | General | Placebo/no treatment | 9 | Serious risk | No serious inconsistency | No Serious Indirectness | No serious imprecision | Undetected | No | Would not reduce effect | No | ⊕〇〇〇 Very Low |
| endpoint scores of the respective rating scales (97) | depressive disorder | Placebo/no treatment | 3 | Serious risk | Serious inconsistency | No Serious Indirectness | Serious imprecision | Undetected | No | Would not reduce effect | No | ⊕〇〇〇 Very Low |
| epilepsy seizure frequency (98) | General | Placebo/no treatment | 2 | Serious risk | No serious inconsistency | No Serious Indirectness | No serious imprecision | Undetected | No | Would not reduce effect | No | ⊕〇〇〇 Very Low |
| Hamilton Depression Rating Scale score (99) | depressive disorder | Placebo/no treatment | 2 | Serious risk | No serious inconsistency | No Serious Indirectness | No serious imprecision | Undetected | Very large | Would reduce effect | No | ⊕⊕⊕⊕High |
| BDI: Beck Depression Inventory/ HAMD: Hamilton Depression Scale (100) | patients with unipolar depressive illness | Placebo/no treatment | 4 | Serious risk | Very serious inconsistency | No Serious Indirectness | Serious | Undetected | No | Would not reduce effect | No | ⊕〇〇〇 Very Low |
| **Pregnancy outcomes** | | | | | | | | | | | | |
| gestational hypertension/preeclampsia (61) | Pregnancy | Placebo/no treatment | 2 | Serious risk | No serious inconsistency | No Serious Indirectness | No serious imprecision | a | No | Would reduce effect | No | ⊕⊕⊕⊕High |
| Length of gestation (69) | Pregnancy | Placebo/no treatment | 6 | Serious risk | Serious inconsistency | No Serious Indirectness | No serious imprecision | NA | No | Would not reduce effect | No | ⊕⊕〇〇 Low |
| Low pre-delivery serum folate (70) | Pregnancy | Placebo/no treatment | 2 | Serious risk | No serious inconsistency | No Serious Indirectness | No serious imprecision | Undetected | Large | would reduce effect | No | ⊕⊕⊕⊕High |
| Miscarriage (68) | Pregnancy | Placebo/no treatment | 5 | Serious risk | No serious inconsistency | No Serious Indirectness | No serious imprecision | Undetected | No | Would not reduce effect | No | ⊕⊕⊕〇 Moderate |
| Multiple pregnancy (68) | Pregnancy | Placebo/no treatment | 4 | Serious risk | No serious inconsistency | No Serious Indirectness | No serious imprecision | Undetected | No | Would not reduce effect | No | ⊕⊕⊕〇 Moderate |
| Pre-delivery anaemia (70) | Pregnancy | Placebo/no treatment | 8 | Serious risk | Very serious inconsistency | No Serious Indirectness | No serious imprecision | Undetected | No | would not reduce effect | No | ⊕〇〇〇 Very Low |
| pre-delivery hemoglobin level (70) | Pregnancy | Placebo/no treatment | 12 | Serious risk | Very serious inconsistency | No Serious Indirectness | Very serious imprecision | Undetected | No | would not reduce effect | No | ⊕〇〇〇 Very Low |
| pre-delivery serum folate (70) | Pregnancy | Placebo/no treatment | 8 | Serious risk | Very serious inconsistency | No Serious Indirectness | Serious imprecision | Undetected | No | would not reduce effect | No | ⊕〇〇〇 Very Low |
| **Diabetes related outcomes** | | | | | | | | | | | | |
| Fasting Glucose (101) | Metabolic Diseases | Placebo/no treatment | 10 | Serious risk | Serious inconsistency | No Serious Indirectness | No serious imprecision | Undetected | No | Would not reduce effect | No | ⊕⊕〇〇 Low |
| Fasting Glucose (102) | General | Placebo/no treatment | 15 | Serious risk | Serious inconsistency | No Serious Indirectness | No serious imprecision | Undetected | No | would reduce effect | No | ⊕⊕⊕〇 Moderate |
| HbA1c (103) | type 2 diabetes | Placebo/no treatment | 3 | Serious risk | Very serious inconsistency | No Serious Indirectness | No serious imprecision | Undetected | Large | Would not reduce effect | No | ⊕〇〇〇 Very Low |
| HbA1c (101) | Metabolic Diseases | Placebo/no treatment | 6 | Serious risk | Serious inconsistency | No Serious Indirectness | No serious imprecision | Undetected | No | Would not reduce effect | No | ⊕⊕〇〇 Low |
| HbA1c (102) | General | Placebo/no treatment | 4 | Serious risk | Very serious inconsistency | No Serious Indirectness | No serious imprecision | Undetected | No | Would not reduce effect | No | ⊕〇〇〇 Very Low |
| HOMA-IR (101) | Metabolic Diseases | Placebo/no treatment | 9 | Serious risk | Very serious inconsistency | No Serious Indirectness | No serious imprecision | Undetected | No | would reduce effect | No | ⊕⊕〇〇 Low |
| HOMA-IR (102) | General | Placebo/no treatment | 9 | Serious risk | Very serious inconsistency | No Serious Indirectness | No serious imprecision | Undetected | No | would reduce effect | No | ⊕⊕〇〇 Low |
| Insulin (101) | Metabolic Diseases | Placebo/no treatment | 8 | Serious risk | Very serious inconsistency | No Serious Indirectness | No serious imprecision | Undetected | No | would reduce effect | No | ⊕⊕〇〇 Low |
| Insulin (102) | General | Placebo/no treatment | 8 | Serious risk | Serious inconsistency | No Serious Indirectness | No serious imprecision | Undetected | No | would reduce effect | No | ⊕⊕⊕〇 Moderate |
| **Other outcomes** | | | | | | | | | | | | |
| Amputation (74) | Preexisting diseases | Placebo/no treatment | 2 | Serious risk | No serious inconsistency | No Serious Indirectness | No serious imprecision | NA | No | Would not reduce effect | No | ⊕⊕⊕〇 Moderate |
| baseline hyperemic flow (87) | patients with coronary artery disease | Placebo/no treatment | 3 | Serious risk | No serious inconsistency | No Serious Indirectness | Very serious imprecision | Undetected | No | Would not reduce effect | No | ⊕〇〇〇 Very Low |
| Dental Hyperplasia index (98) | General | Placebo/no treatment | 2 | Serious risk | No serious inconsistency | No Serious Indirectness | No serious imprecision | Undetected | No | Would not reduce effect | No | ⊕〇〇〇 Very Low |
| dental Plaque index (98) | General | Placebo/no treatment | 2 | Serious risk | No serious inconsistency | No Serious Indirectness | No serious imprecision | Undetected | No | Would not reduce effect | No | ⊕〇〇〇 Very Low |
| Gingival health index (98) | General | Placebo/no treatment | 2 | Serious risk | No serious inconsistency | No Serious Indirectness | No serious imprecision | Undetected | No | Would not reduce effect | No | ⊕⊕⊕〇 Moderate |
| Liver toxicity (104) | patients receiving methotrexate | Placebo/no treatment | 2 | Serious risk | No serious inconsistency | No Serious Indirectness | No serious imprecision | Undetected | Large | would reduce effect | No | ⊕⊕⊕〇 Moderate |
| Nausea / GI upset (104) | patients receiving methotrexate | Placebo/no treatment | 3 | Serious risk | No serious inconsistency | No Serious Indirectness | No serious imprecision | Undetected | No | would not reduce effect | No | ⊕〇〇〇 Very Low |
| Neutropenia (104) | patients receiving methotrexate | Placebo/no treatment | 2 | Serious risk | No serious inconsistency | No Serious Indirectness | Very serious imprecision | Undetected | No | would not reduce effect | No | ⊕〇〇〇 Very Low |
| plasma homocysteine (103) | type 2 diabetes | Placebo/no treatment | 4 | Serious risk | Serious inconsistency | No Serious Indirectness | No serious imprecision | Undetected | Very large | Would reduce effect | No | ⊕⊕⊕⊕High |
| plasma homocysteine (105) | childbearing age, pregnant and lactating women | Placebo/no treatment | 9 | Serious risk | Serious inconsistency | No Serious Indirectness | No serious imprecision | NA | No | would reduce effect | No | ⊕〇〇〇 Very Low |
| plasma homocysteine (107) | General | Placebo/no treatment | 25 | Serious risk | NA | NA | NA | NA | NA | NA | NA | ⊕⊕⊕〇 Moderate |
| plasma homocysteine (87) | patients with coronary artery disease | Placebo/no treatment | 4 | Serious risk | No serious inconsistency | No Serious Indirectness | No serious imprecision | Undetected | No | Would reduce effect | No | ⊕〇〇〇 Very Low |
| plasma homocysteine (65) | Preexisting diseases | Placebo/no treatment | 12 | Serious risk | Very serious inconsistency | No Serious Indirectness | No serious imprecision | Undetected | No | would reduce effect | No | ⊕⊕〇〇 Low |
| red blood cell folate (105) | childbearing age, pregnant and lactating women | Placebo/no treatment | 9 | Serious risk | Very serious inconsistency | No Serious Indirectness | No serious imprecision | NA | No | would reduce effect | No | ⊕〇〇〇 Very Low |
| red blood cell folate (108) | General | Placebo/no treatment | 7 | Serious risk | Serious inconsistency | No Serious Indirectness | No serious imprecision | Undetected | No | Would reduce effect | No | ⊕⊕⊕〇 Moderate |
| Serum/plasma folate (105) | childbearing age, pregnant and lactating women | Placebo/no treatment | 9 | Serious risk | Very serious inconsistency | No Serious Indirectness | No serious imprecision | NA | No | would reduce effect | No | ⊕⊕〇〇 Low |
| Serum/plasma folate (108) | General | Placebo/no treatment | 17 | Serious risk | Serious inconsistency | No Serious Indirectness | No serious imprecision | Undetected | Large | Would reduce effect | No | ⊕⊕⊕⊕High |
| Stomatitis (104) | patients receiving methotrexate | Placebo/no treatment | 2 | Serious risk | No serious inconsistency | No Serious Indirectness | Serious imprecision | Undetected | No | would not reduce effect | No | ⊕〇〇〇 Very Low |
| Total withdrawals (104) | patients receiving methotrexate | Placebo/no treatment | 3 | Serious risk | No serious inconsistency | No Serious Indirectness | No serious imprecision | Undetected | No | would reduce effect | No | ⊕⊕〇〇 Low |

### Supplementary Table S10. Comparison of overlapping results between meta-analysis of observational Studies and RCTs.

| **Outcomes** | **Meta-analysis of RCTs** | | | **Meta-analysis of observational studies** | | | **Concordant**  **direction** | **CI excluded null (Significance)** | |
| --- | --- | --- | --- | --- | --- | --- | --- | --- | --- |
|  | Exposure | Metric | Effect size (95% CI) | Exposure | Metric | Effect size (95% CI) | Concordant  direction | randomized controlled trials | Observational studies |
| Cleft palate | Folate supplement | RR | 0.73 (0.05, 10.89) | folate supplement | OR | 0.74 (0.54, 1.03) | Yes | No | No |
| Neural tube defects | Folate supplement | RR | 0.59 (0.52, 0.68) | Folate supplement | RR | 0.33 (0.18, 0.62) | Yes | Yes | Yes |
| Neural tube defects recurrence | Folate supplement | RR | 0.30 (0.14, 0.65) | Folate supplement | OR | 0.63 (0.15, 2.64) | No | Yes | No |
| Colorectal cancer | Folate supplement | RR | 1.00 (0.82, 1.22) | Dietary folate intake | OR | 0.87 (0.74, 1.02) | Yes | No | No |
|  |  |  |  | Dietary folate intake | HR | 0.92 (0.81, 1.05) | Yes | No | No |
|  |  |  |  | Folate supplement | RR | 0.87 (0.74, 1.01) | Yes | No | No |
|  |  |  |  | Total folate intake | RR | 0.88 (0.81, 0.95) | No | No | Yes |
|  |  |  |  | RBC folate | RR | 1.04 (0.84, 1.29) | Yes | No | No |
|  |  |  |  | Circulating folate | RR | 1.01 (0.87, 1.17) | Yes | No | No |
| Gestational  hypertension/preeclampsia | Folate supplement | RR | 0.62 (0.44, 0.89) | Folate supplement | RR | 0.92 (0.79, 1.09) | No | Yes | No |

## Supplementary Figures


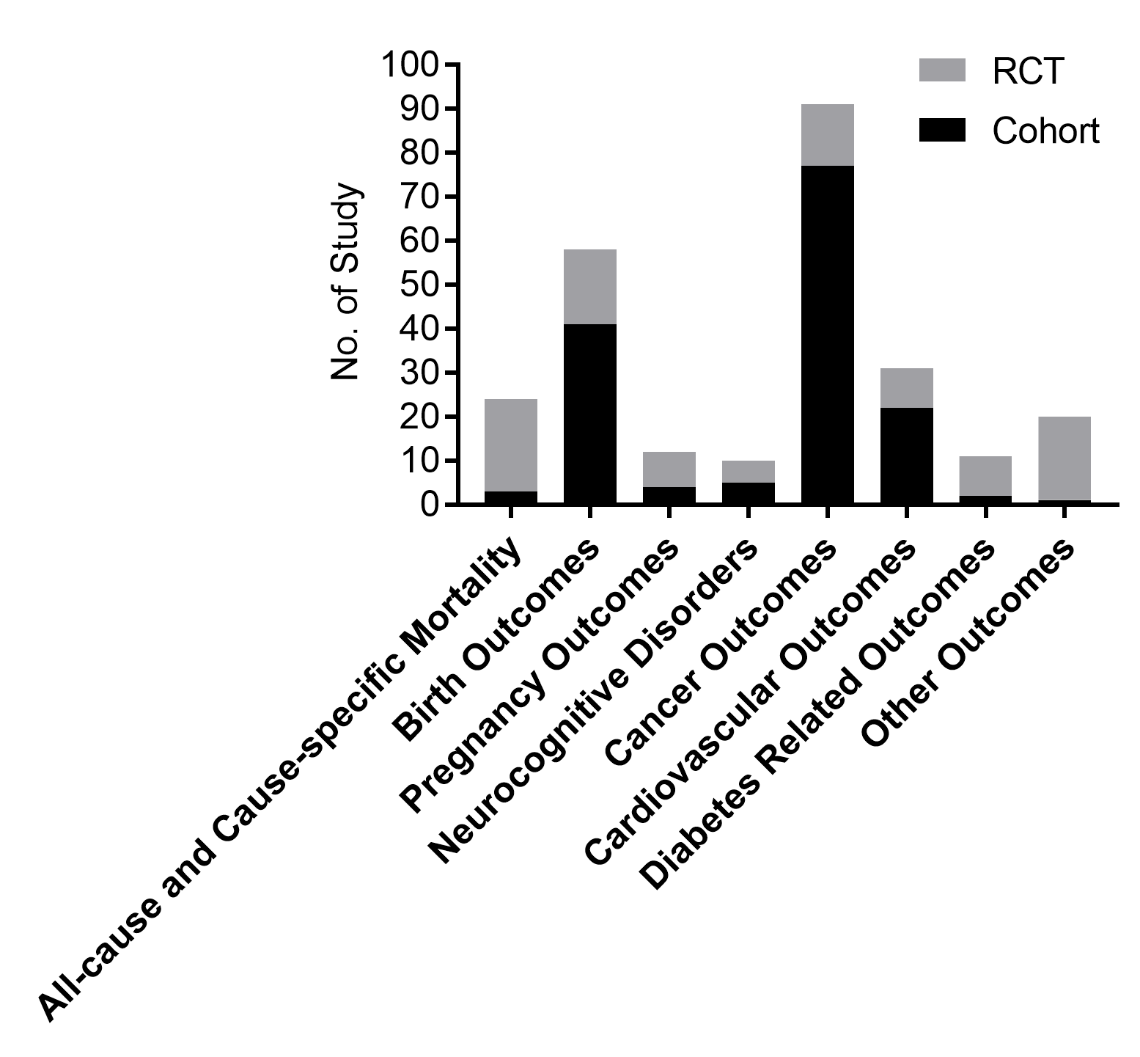


**Supplementary Figure S1 |** The histogram of outcomes category stratified by study type.


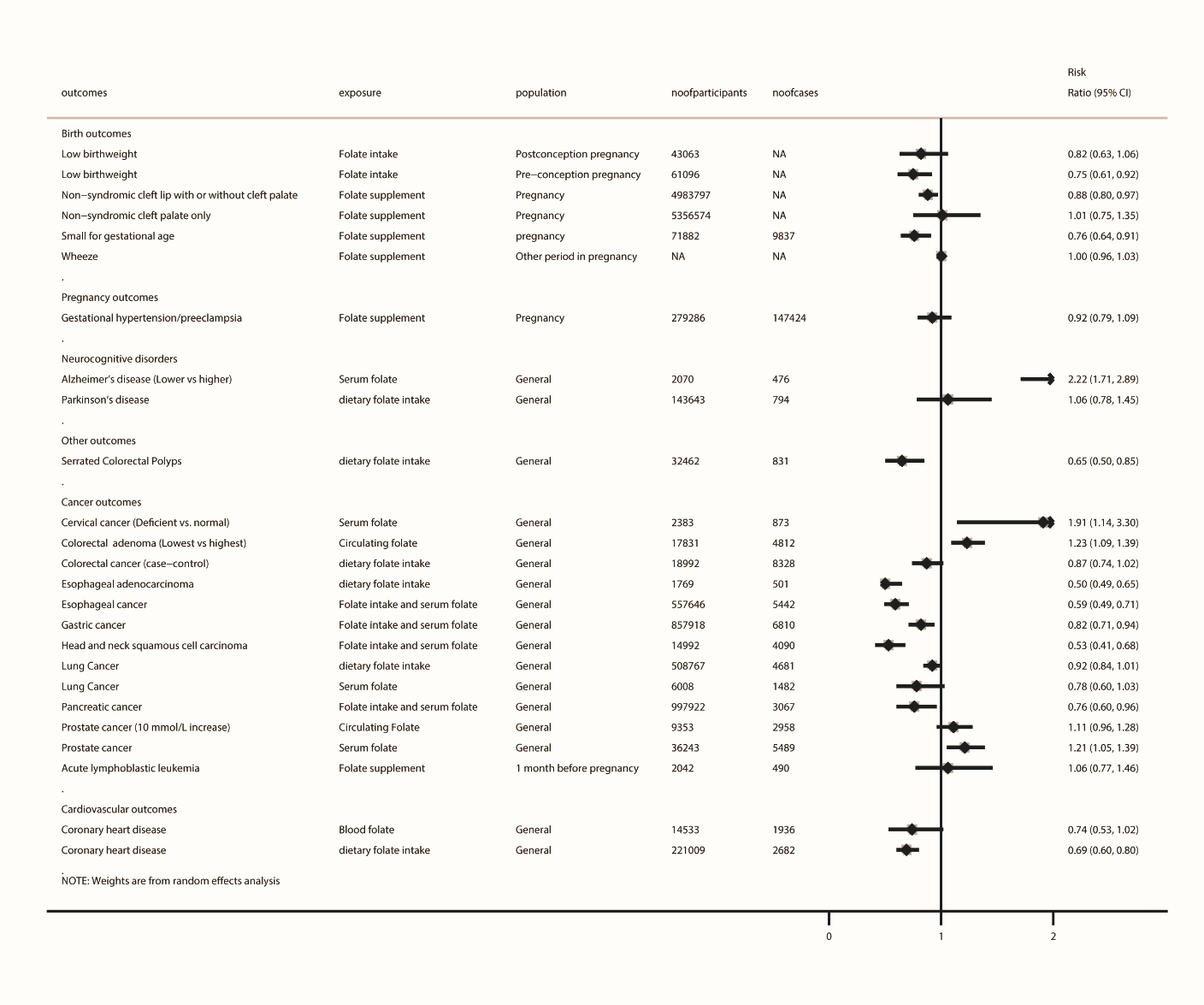


**Supplementary Figure S2 |** Summary of moderate and high evidence with meta-analysis of observational studies.


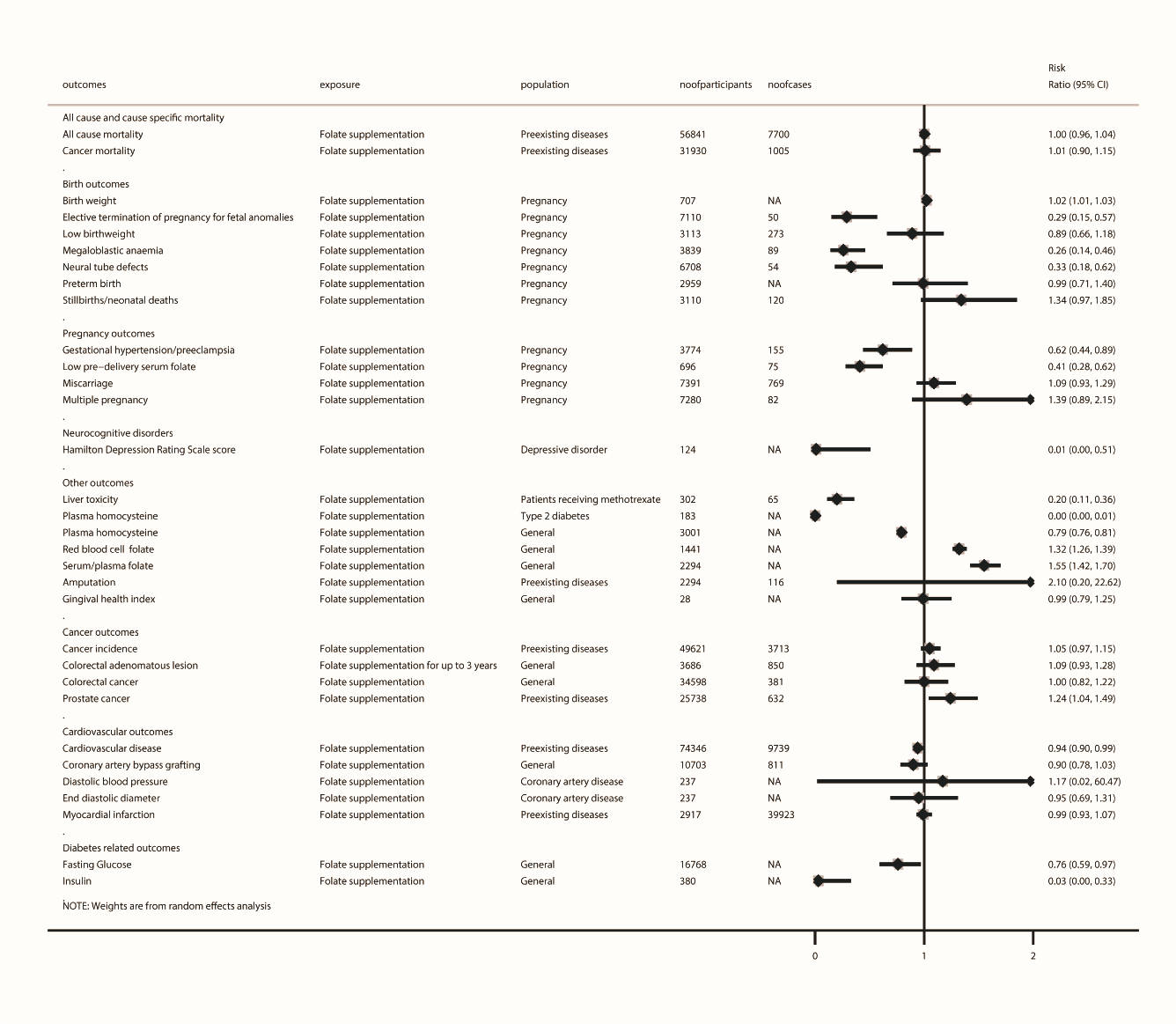


**Supplementary Figure S3 |** Summary of moderate and high evidence with meta-analysis of randomized controlled trials.

## Reference

1. Li B, Lu Y, Wang L, Zhang CX. Folate intake and breast cancer prognosis: a meta-analysis of prospective observational studies. *European journal of cancer prevention : the official journal of the European Cancer Prevention Organisation (ECP)* (2015) 24(2):113-21. Epub 2014/05/03. doi: 10.1097/cej.0000000000000028. PubMed PMID: 24787380.

2. Wang T, Zhang HP, Zhang X, Liang ZA, Ji YL, Wang G. Is Folate Status a Risk Factor for Asthma or Other Allergic Diseases? *Allergy, asthma & immunology research* (2015) 7(6):538-46. Epub 2015/09/04. doi: 10.4168/aair.2015.7.6.538. PubMed PMID: 26333700; PubMed Central PMCID: PMCPMC4605926.

3. Crider KS, Cordero AM, Qi YP, Mulinare J, Dowling NF, Berry RJ. Prenatal folic acid and risk of asthma in children: a systematic review and meta-analysis. *The American journal of clinical nutrition* (2013) 98(5):1272-81. Epub 2013/09/06. doi: 10.3945/ajcn.113.065623. PubMed PMID: 24004895; PubMed Central PMCID: PMCPMC5369603.

4. Yang L, Jiang L, Bi M, Jia X, Wang Y, He C, et al. High dose of maternal folic acid supplementation is associated to infant asthma. *Food Chem Toxicol* (2015) 75:88-93. doi: 10.1016/j.fct.2014.11.006. PubMed PMID: 25449200.

5. Wang M, Li K, Zhao D, Li L. The association between maternal use of folic acid supplements during pregnancy and risk of autism spectrum disorders in children: a meta-analysis. *Molecular autism* (2017) 8:51. Epub 2017/10/14. doi: 10.1186/s13229-017-0170-8. PubMed PMID: 29026508; PubMed Central PMCID: PMCPMC5625821.

6. Badovinac RL, Werler MM, Williams PL, Kelsey KT, Hayes C. Folic acid-containing supplement consumption during pregnancy and risk for oral clefts: a meta-analysis. *Birth defects research Part A, Clinical and molecular teratology* (2007) 79(1):8-15. Epub 2006/11/30. doi: 10.1002/bdra.20315. PubMed PMID: 17133404.

7. Johnson CY, Little J. Folate intake, markers of folate status and oral clefts: is the evidence converging? *International journal of epidemiology* (2008) 37(5):1041-58. Epub 2008/06/28. doi: 10.1093/ije/dyn098. PubMed PMID: 18583393.

8. Jahanbin A, Shadkam E, Miri HH, Shirazi AS, Abtahi M. Maternal Folic Acid Supplementation and the Risk of Oral Clefts in Offspring. *The Journal of craniofacial surgery* (2018). Epub 2018/05/16. doi: 10.1097/scs.0000000000004488. PubMed PMID: 29762322.

9. Millacura N, Pardo R, Cifuentes L, Suazo J. Effects of folic acid fortification on orofacial clefts prevalence: a meta-analysis. *Public health nutrition* (2017) 20(12):2260-8. Epub 2017/05/24. doi: 10.1017/s1368980017000878. PubMed PMID: 28534456.

10. Feng Y, Wang S, Chen R, Tong X, Wu Z, Mo X. Maternal folic acid supplementation and the risk of congenital heart defects in offspring: a meta-analysis of epidemiological observational studies. *Scientific reports* (2015) 5:8506. Epub 2015/02/18. doi: 10.1038/srep08506. PubMed PMID: 25687545; PubMed Central PMCID: PMCPMC4330542.

11. Xu A, Cao X, Lu Y, Li H, Zhu Q, Chen X, et al. A Meta-Analysis of the Relationship Between Maternal Folic Acid Supplementation and the Risk of Congenital Heart Defects. *International heart journal* (2016) 57(6):725-8. Epub 2016/11/11. doi: 10.1536/ihj.16-054. PubMed PMID: 27829639.

12. Dean SV, Lassi ZS, Imam AM, Bhutta ZA. Preconception care: nutritional risks and interventions. *Reproductive health* (2014) 11 Suppl 3:S3. Epub 2014/11/22. doi: 10.1186/1742-4755-11-s3-s3. PubMed PMID: 25415364; PubMed Central PMCID: PMCPMC4196560.

13. Hodgetts VA, Morris RK, Francis A, Gardosi J, Ismail KM. Effectiveness of folic acid supplementation in pregnancy on reducing the risk of small-for-gestational age neonates: a population study, systematic review and meta-analysis. *BJOG : an international journal of obstetrics and gynaecology* (2015) 122(4):478-90. Epub 2014/11/27. doi: 10.1111/1471-0528.13202. PubMed PMID: 25424556.

14. Imdad A, Yakoob MY, Bhutta ZA. The effect of folic acid, protein energy and multiple micronutrient supplements in pregnancy on stillbirths. *BMC public health* (2011) 11 Suppl 3:S4. Epub 2011/04/29. doi: 10.1186/1471-2458-11-s3-s4. PubMed PMID: 21501455; PubMed Central PMCID: PMCPMC3231910.

15. Blencowe H, Cousens S, Modell B, Lawn J. Folic acid to reduce neonatal mortality from neural tube disorders. *International journal of epidemiology* (2010) 39 Suppl 1:i110-21. Epub 2010/04/02. doi: 10.1093/ije/dyq028. PubMed PMID: 20348114; PubMed Central PMCID: PMCPMC2845867.

16. Zhang Q, Wang Y, Xin X, Zhang Y, Liu D, Peng Z, et al. Effect of folic acid supplementation on preterm delivery and small for gestational age births: A systematic review and meta-analysis. *Reproductive toxicology (Elmsford, NY)* (2017) 67:35-41. Epub 2016/11/20. doi: 10.1016/j.reprotox.2016.11.012. PubMed PMID: 27856370.

17. He H, Shui B. Folate intake and risk of bladder cancer: a meta-analysis of epidemiological studies. *International journal of food sciences and nutrition* (2014) 65(3):286-92. Epub 2013/12/18. doi: 10.3109/09637486.2013.866641. PubMed PMID: 24328495.

18. Larsson SC, Giovannucci E, Wolk A. Folate and Risk of Breast Cancer: A Meta-analysis. *JNCI: Journal of the National Cancer Institute* (2007) 99(1):64-76. doi: 10.1093/jnci/djk006.

19. Tio M, Andrici J, Eslick GD. Folate intake and the risk of breast cancer: a systematic review and meta-analysis. *Breast cancer research and treatment* (2014) 145(2):513-24. Epub 2014/04/30. doi: 10.1007/s10549-014-2969-8. PubMed PMID: 24777595.

20. Chen P, Li C, Li X, Li J, Chu R, Wang H. Higher dietary folate intake reduces the breast cancer risk: a systematic review and meta-analysis. *British journal of cancer* (2014) 110(9):2327-38. Epub 2014/03/29. doi: 10.1038/bjc.2014.155. PubMed PMID: 24667649; PubMed Central PMCID: PMCPMC4007237.

21. Liu M, Cui LH, Ma AG, Li N, Piao JM. Lack of effects of dietary folate intake on risk of breast cancer: an updated meta-analysis of prospective studies. *Asian Pacific journal of cancer prevention : APJCP* (2014) 15(5):2323-8. Epub 2014/04/11. PubMed PMID: 24716978.

22. Lewis SJ, Harbord RM, Harris R, Smith GD. Meta-analyses of observational and genetic association studies of folate intakes or levels and breast cancer risk. *Journal of the National Cancer Institute* (2006) 98(22):1607-22. Epub 2006/11/16. doi: 10.1093/jnci/djj440. PubMed PMID: 17105984.

23. Zhang YF, Shi WW, Gao HF, Zhou L, Hou AJ, Zhou YH. Folate intake and the risk of breast cancer: a dose-response meta-analysis of prospective studies. *PloS one* (2014) 9(6):e100044. Epub 2014/06/17. doi: 10.1371/journal.pone.0100044. PubMed PMID: 24932496; PubMed Central PMCID: PMCPMC4059748.

24. Zhou X, Meng Y. Association between serum folate level and cervical cancer: a meta-analysis. *Archives of gynecology and obstetrics* (2016) 293(4):871-7. Epub 2015/09/01. doi: 10.1007/s00404-015-3852-5. PubMed PMID: 26319154.

25. Myung SK, Ju W, Kim SC, Kim H. Vitamin or antioxidant intake (or serum level) and risk of cervical neoplasm: a meta-analysis. *BJOG : an international journal of obstetrics and gynaecology* (2011) 118(11):1285-91. Epub 2011/07/14. doi: 10.1111/j.1471-0528.2011.03032.x. PubMed PMID: 21749626.

26. Kim DH, Smith-Warner SA, Spiegelman D, Yaun SS, Colditz GA, Freudenheim JL, et al. Pooled analyses of 13 prospective cohort studies on folate intake and colon cancer. *Cancer causes & control : CCC* (2010) 21(11):1919-30. Epub 2010/09/08. doi: 10.1007/s10552-010-9620-8. PubMed PMID: 20820900; PubMed Central PMCID: PMCPMC3082430.

27. Park YM, Youn J, Cho CH, Kim SH, Lee JE. Circulating folate levels and colorectal adenoma: a case-control study and a meta-analysis. *Nutrition research and practice* (2017) 11(5):419-29. Epub 2017/10/11. doi: 10.4162/nrp.2017.11.5.419. PubMed PMID: 28989579; PubMed Central PMCID: PMCPMC5621365.

28. Chuang SC, Rota M, Gunter MJ, Zeleniuch-Jacquotte A, Eussen SJ, Vollset SE, et al. Quantifying the dose-response relationship between circulating folate concentrations and colorectal cancer in cohort studies: a meta-analysis based on a flexible meta-regression model. *American journal of epidemiology* (2013) 178(7):1028-37. Epub 2013/07/19. doi: 10.1093/aje/kwt083. PubMed PMID: 23863758.

29. Sanjoaquin MA, Allen N, Couto E, Roddam AW, Key TJ. Folate intake and colorectal cancer risk: a meta-analytical approach. *International journal of cancer* (2005) 113(5):825-8. Epub 2004/10/23. doi: 10.1002/ijc.20648. PubMed PMID: 15499620.

30. Kennedy DA, Stern SJ, Moretti M, Matok I, Sarkar M, Nickel C, et al. Folate intake and the risk of colorectal cancer: a systematic review and meta-analysis. *Cancer epidemiology* (2011) 35(1):2-10. Epub 2010/12/24. doi: 10.1016/j.canep.2010.11.004. PubMed PMID: 21177150.

31. Heine-Broring RC, Winkels RM, Renkema JM, Kragt L, van Orten-Luiten AC, Tigchelaar EF, et al. Dietary supplement use and colorectal cancer risk: a systematic review and meta-analyses of prospective cohort studies. *International journal of cancer* (2015) 136(10):2388-401. Epub 2014/10/23. doi: 10.1002/ijc.29277. PubMed PMID: 25335850.

32. Moazzen S, Dolatkhah R, Tabrizi JS, Shaarbafi J, Alizadeh BZ, de Bock GH, et al. Folic acid intake and folate status and colorectal cancer risk: A systematic review and meta-analysis. *Clinical Nutrition* (2017). doi: <https://doi.org/10.1016/j.clnu.2017.10.010>.

33. Burr NE, Hull MA, Subramanian V. Folic Acid Supplementation May Reduce Colorectal Cancer Risk in Patients With Inflammatory Bowel Disease: A Systematic Review and Meta-Analysis. *Journal of clinical gastroenterology* (2017) 51(3):247-53. Epub 2016/02/26. doi: 10.1097/mcg.0000000000000498. PubMed PMID: 26905603.

34. Liu Y, Yu Q, Zhu Z, Zhang J, Chen M, Tang P, et al. Vitamin and multiple-vitamin supplement intake and incidence of colorectal cancer: a meta-analysis of cohort studies. *Medical oncology (Northwood, London, England)* (2015) 32(1):434. Epub 2014/12/11. doi: 10.1007/s12032-014-0434-5. PubMed PMID: 25491145.

35. Du L, Wang Y, Zhang H, Zhang H, Gao Y. Folate intake and the risk of endometrial cancer: A meta-analysis. *Oncotarget* (2016) 7(51):85176-84. Epub 2016/11/12. doi: 10.18632/oncotarget.13211. PubMed PMID: 27835893; PubMed Central PMCID: PMCPMC5356727.

36. Larsson SC, Giovannucci E, Wolk A. Folate intake, MTHFR polymorphisms, and risk of esophageal, gastric, and pancreatic cancer: a meta-analysis. *Gastroenterology* (2006) 131(4):1271-83. Epub 2006/10/13. doi: 10.1053/j.gastro.2006.08.010. PubMed PMID: 17030196.

37. Zhao Y, Guo C, Hu H, Zheng L, Ma J, Jiang L, et al. Folate intake, serum folate levels and esophageal cancer risk: an overall and dose-response meta-analysis. *Oncotarget* (2017) 8(6):10458-69. Epub 2017/01/07. doi: 10.18632/oncotarget.14432. PubMed PMID: 28060731; PubMed Central PMCID: PMCPMC5354672.

38. Ni Y, Du J, Yin X, Lu M. Folate intake, serum folate, and risk of esophageal cancer: a systematic review and dose-response meta-analysis. *European journal of cancer prevention : the official journal of the European Cancer Prevention Organisation (ECP)* (2018). Epub 2018/03/24. doi: 10.1097/cej.0000000000000441. PubMed PMID: 29570105.

39. Tio M, Andrici J, Cox MR, Eslick GD. Folate intake and the risk of upper gastrointestinal cancers: a systematic review and meta-analysis. *Journal of gastroenterology and hepatology* (2014) 29(2):250-8. Epub 2013/11/15. doi: 10.1111/jgh.12446. PubMed PMID: 24224911.

40. Liu YX, Wang B, Wan MH, Tang WF, Huang FK, Li C. Meta-analysis of the relationship between the Metholenetetrahydrofolate reductase C677T genetic polymorphism, folate intake and esophageal cancer. *Asian Pacific journal of cancer prevention : APJCP* (2011) 12(1):247-52. Epub 2011/04/27. PubMed PMID: 21517266.

41. Liu W, Zhou H, Zhu Y, Tie C. Associations between dietary folate intake and risks of esophageal, gastric and pancreatic cancers: an overall and dose-response meta-analysis. *Oncotarget* (2017) 8(49):86828-42. Epub 2017/11/22. doi: 10.18632/oncotarget.18775. PubMed PMID: 29156838; PubMed Central PMCID: PMCPMC5689728.

42. Fan C, Yu S, Zhang S, Ding X, Su J, Cheng Z. Association between folate intake and risk of head and neck squamous cell carcinoma: An overall and dose-response PRISMA meta-analysis. *Medicine* (2017) 96(42):e8182. Epub 2017/10/20. doi: 10.1097/md.0000000000008182. PubMed PMID: 29049201; PubMed Central PMCID: PMCPMC5662367.

43. Dai WM, Yang B, Chu XY, Wang YQ, Zhao M, Chen L, et al. Association between folate intake, serum folate levels and the risk of lung cancer: a systematic review and meta-analysis. *Chinese medical journal* (2013) 126(10):1957-64. Epub 2013/05/16. PubMed PMID: 23673118.

44. Zhang YF, Zhou L, Zhang HW, Hou AJ, Gao HF, Zhou YH. Association between folate intake and the risk of lung cancer: a dose-response meta-analysis of prospective studies. *PloS one* (2014) 9(4):e93465. Epub 2014/04/10. doi: 10.1371/journal.pone.0093465. PubMed PMID: 24713625; PubMed Central PMCID: PMCPMC3979671.

45. Li C, Chen P, Hu P, Li M, Li X, Guo H, et al. Folate intake and MTHFR polymorphism C677T is not associated with ovarian cancer risk: evidence from the meta-analysis. *Molecular biology reports* (2013) 40(12):6547-60. Epub 2013/10/17. doi: 10.1007/s11033-013-2686-0. PubMed PMID: 24129496.

46. Lin HL, An QZ, Wang QZ, Liu CX. Folate intake and pancreatic cancer risk: an overall and dose-response meta-analysis. *Public health* (2013) 127(7):607-13. Epub 2013/06/19. doi: 10.1016/j.puhe.2013.04.008. PubMed PMID: 23769243.

47. Wien TN, Pike E, Wisloff T, Staff A, Smeland S, Klemp M. Cancer risk with folic acid supplements: a systematic review and meta-analysis. *BMJ open* (2012) 2(1):e000653. Epub 2012/01/14. doi: 10.1136/bmjopen-2011-000653. PubMed PMID: 22240654; PubMed Central PMCID: PMCPMC3278486.

48. Wang R, Zheng Y, Huang JY, Zhang AQ, Zhou YH, Wang JN. Folate intake, serum folate levels, and prostate cancer risk: a meta-analysis of prospective studies. *BMC public health* (2014) 14:1326. Epub 2014/12/30. doi: 10.1186/1471-2458-14-1326. PubMed PMID: 25543518; PubMed Central PMCID: PMCPMC4320532.

49. Tio M, Andrici J, Cox MR, Eslick GD. Folate intake and the risk of prostate cancer: a systematic review and meta-analysis. *Prostate cancer and prostatic diseases* (2014) 17(3):213-9. Epub 2014/05/14. doi: 10.1038/pcan.2014.16. PubMed PMID: 24819234.

50. Price AJ, Travis RC, Appleby PN, Albanes D, Barricarte Gurrea A, Bjorge T, et al. Circulating Folate and Vitamin B12 and Risk of Prostate Cancer: A Collaborative Analysis of Individual Participant Data from Six Cohorts Including 6875 Cases and 8104 Controls. *European urology* (2016) 70(6):941-51. Epub 2016/04/12. doi: 10.1016/j.eururo.2016.03.029. PubMed PMID: 27061263; PubMed Central PMCID: PMCPMC5094800.

51. Collin SM, Metcalfe C, Refsum H, Lewis SJ, Zuccolo L, Smith GD, et al. Circulating folate, vitamin B12, homocysteine, vitamin B12 transport proteins, and risk of prostate cancer: a case-control study, systematic review, and meta-analysis. *Cancer epidemiology, biomarkers & prevention : a publication of the American Association for Cancer Research, cosponsored by the American Society of Preventive Oncology* (2010) 19(6):1632-42. Epub 2010/05/27. doi: 10.1158/1055-9965.epi-10-0180. PubMed PMID: 20501771; PubMed Central PMCID: PMCPMC3759018.

52. Mao B, Li Y, Zhang Z, Chen C, Chen Y, Ding C, et al. One-Carbon Metabolic Factors and Risk of Renal Cell Cancer: A Meta-Analysis. *PloS one* (2015) 10(10):e0141762. Epub 2015/10/30. doi: 10.1371/journal.pone.0141762. PubMed PMID: 26513161; PubMed Central PMCID: PMCPMC4625965.

53. Milne E, Royle JA, Miller M, Bower C, de Klerk NH, Bailey HD, et al. Maternal folate and other vitamin supplementation during pregnancy and risk of acute lymphoblastic leukemia in the offspring. *International journal of cancer* (2010) 126(11):2690-9. Epub 2009/10/20. doi: 10.1002/ijc.24969. PubMed PMID: 19839053.

54. Wang ZM, Zhou B, Nie ZL, Gao W, Wang YS, Zhao H, et al. Folate and risk of coronary heart disease: a meta-analysis of prospective studies. *Nutrition, metabolism, and cardiovascular diseases : NMCD* (2012) 22(10):890-9. Epub 2011/09/20. doi: 10.1016/j.numecd.2011.04.011. PubMed PMID: 21924595.

55. Shen L, Ji HF. Associations between Homocysteine, Folic Acid, Vitamin B12 and Alzheimer's Disease: Insights from Meta-Analyses. *Journal of Alzheimer's disease : JAD* (2015) 46(3):777-90. Epub 2015/04/10. doi: 10.3233/jad-150140. PubMed PMID: 25854931.

56. Michelakos T, Kousoulis AA, Katsiardanis K, Dessypris N, Anastasiou A, Katsiardani KP, et al. Serum folate and B12 levels in association with cognitive impairment among seniors: results from the VELESTINO study in Greece and meta-analysis. *Journal of aging and health* (2013) 25(4):589-616. Epub 2013/04/10. doi: 10.1177/0898264313482488. PubMed PMID: 23569157.

57. Gilbody S, Lightfoot T, Sheldon T. Is low folate a risk factor for depression? A meta-analysis and exploration of heterogeneity. *Journal of epidemiology and community health* (2007) 61(7):631-7. Epub 2007/06/15. doi: 10.1136/jech.2006.050385. PubMed PMID: 17568057; PubMed Central PMCID: PMCPMC2465760.

58. Petridou ET, Kousoulis AA, Michelakos T, Papathoma P, Dessypris N, Papadopoulos FC, et al. Folate and B12 serum levels in association with depression in the aged: a systematic review and meta-analysis. *Aging & mental health* (2016) 20(9):965-73. Epub 2015/06/10. doi: 10.1080/13607863.2015.1049115. PubMed PMID: 26055921.

59. Shen L. Associations between B Vitamins and Parkinson's Disease. *Nutrients* (2015) 7(9):7197-208. Epub 2015/09/08. doi: 10.3390/nu7095333. PubMed PMID: 26343714; PubMed Central PMCID: PMCPMC4586528.

60. Yang X, Chen H, Du Y, Wang S, Wang Z. Periconceptional folic acid fortification for the risk of gestational hypertension and pre-eclampsia: a meta-analysis of prospective studies. *Maternal & child nutrition* (2016) 12(4):669-79. Epub 2015/08/12. doi: 10.1111/mcn.12209. PubMed PMID: 26260406.

61. Hua X, Zhang J, Guo Y, Shen M, Gaudet L, Janoudi G, et al. Effect of folic acid supplementation during pregnancy on gestational hypertension/preeclampsia: A systematic review and meta-analysis. *Hypertension in pregnancy* (2016) 35(4):447-60. Epub 2016/11/03. doi: 10.1080/10641955.2016.1183673. PubMed PMID: 27315401.

62. Bailie L, Loughrey MB, Coleman HG. Lifestyle Risk Factors for Serrated Colorectal Polyps: A&#xa0;Systematic Review and Meta-analysis. *Gastroenterology* (2017) 152(1):92-104. doi: 10.1053/j.gastro.2016.09.003.

63. Schwingshackl L, Boeing H, Stelmach-Mardas M, Gottschald M, Dietrich S, Hoffmann G, et al. Dietary Supplements and Risk of Cause-Specific Death, Cardiovascular Disease, and Cancer: A Systematic Review and Meta-Analysis of Primary Prevention Trials. *Advances in nutrition (Bethesda, Md)* (2017) 8(1):27-39. Epub 2017/01/18. doi: 10.3945/an.116.013516. PubMed PMID: 28096125; PubMed Central PMCID: PMCPMC5227980.

64. Bazzano LA, Reynolds K, Holder KN, He J. Effect of folic acid supplementation on risk of cardiovascular diseases: a meta-analysis of randomized controlled trials. *Jama* (2006) 296(22):2720-6. Epub 2006/12/14. doi: 10.1001/jama.296.22.2720. PubMed PMID: 17164458.

65. Miller ER, 3rd, Juraschek S, Pastor-Barriuso R, Bazzano LA, Appel LJ, Guallar E. Meta-analysis of folic acid supplementation trials on risk of cardiovascular disease and risk interaction with baseline homocysteine levels. *The American journal of cardiology* (2010) 106(4):517-27. Epub 2010/08/10. doi: 10.1016/j.amjcard.2010.03.064. PubMed PMID: 20691310.

66. Yang HT, Lee M, Hong KS, Ovbiagele B, Saver JL. Efficacy of folic acid supplementation in cardiovascular disease prevention: an updated meta-analysis of randomized controlled trials. *European journal of internal medicine* (2012) 23(8):745-54. Epub 2012/08/14. doi: 10.1016/j.ejim.2012.07.004. PubMed PMID: 22884409.

67. Qin X, Cui Y, Shen L, Sun N, Zhang Y, Li J, et al. Folic acid supplementation and cancer risk: a meta-analysis of randomized controlled trials. *International journal of cancer* (2013) 133(5):1033-41. Epub 2013/01/23. doi: 10.1002/ijc.28038. PubMed PMID: 23338728.

68. De-Regil LM, Pena-Rosas JP, Fernandez-Gaxiola AC, Rayco-Solon P. Effects and safety of periconceptional oral folate supplementation for preventing birth defects. *The Cochrane database of systematic reviews* (2015) (12):Cd007950. Epub 2015/12/15. doi: 10.1002/14651858.CD007950.pub3. PubMed PMID: 26662928.

69. Fekete K, Berti C, Trovato M, Lohner S, Dullemeijer C, Souverein OW, et al. Effect of folate intake on health outcomes in pregnancy: a systematic review and meta-analysis on birth weight, placental weight and length of gestation. *Nutrition journal* (2012) 11:75. Epub 2012/09/21. doi: 10.1186/1475-2891-11-75. PubMed PMID: 22992251; PubMed Central PMCID: PMCPMC3499376.

70. Lassi ZS, Salam RA, Haider BA, Bhutta ZA. Folic acid supplementation during pregnancy for maternal health and pregnancy outcomes. *The Cochrane database of systematic reviews* (2013) (3):Cd006896. Epub 2013/04/02. doi: 10.1002/14651858.CD006896.pub2. PubMed PMID: 23543547.

71. Saccone G, Berghella V. Folic acid supplementation in pregnancy to prevent preterm birth: a systematic review and meta-analysis of randomized controlled trials. *European journal of obstetrics, gynecology, and reproductive biology* (2016) 199:76-81. Epub 2016/02/24. doi: 10.1016/j.ejogrb.2016.01.042. PubMed PMID: 26901401.

72. Baggott JE, Oster RA, Tamura T. Meta-analysis of cancer risk in folic acid supplementation trials. *Cancer epidemiology* (2012) 36(1):78-81. Epub 2011/10/25. doi: 10.1016/j.canep.2011.05.003. PubMed PMID: 22018948.

73. Vollset SE, Clarke R, Lewington S, Ebbing M, Halsey J, Lonn E, et al. Effects of folic acid supplementation on overall and site-specific cancer incidence during the randomised trials: meta-analyses of data on 50 000 individuals. *The Lancet* (2013) 381(9871):1029-36. doi: <https://doi.org/10.1016/S0140-6736(12)62001-7>.

74. Zhou YH, Tang JY, Wu MJ, Lu J, Wei X, Qin YY, et al. Effect of folic acid supplementation on cardiovascular outcomes: a systematic review and meta-analysis. *PloS one* (2011) 6(9):e25142. Epub 2011/10/08. doi: 10.1371/journal.pone.0025142. PubMed PMID: 21980387; PubMed Central PMCID: PMCPMC3182189.

75. Carroll C, Cooper K, Papaioannou D, Hind D, Tappenden P, Pilgrim H, et al. Meta-analysis: folic acid in the chemoprevention of colorectal adenomas and colorectal cancer. *Alimentary pharmacology & therapeutics* (2010) 31(7):708-18. Epub 2010/01/21. doi: 10.1111/j.1365-2036.2010.04238.x. PubMed PMID: 20085565.

76. Figueiredo JC, Mott LA, Giovannucci E, Wu K, Cole B, Grainge MJ, et al. Folic acid and prevention of colorectal adenomas: a combined analysis of randomized clinical trials. *International journal of cancer* (2011) 129(1):192-203. Epub 2010/12/21. doi: 10.1002/ijc.25872. PubMed PMID: 21170989; PubMed Central PMCID: PMCPMC3753215.

77. Ibrahim EM, Zekri JM. Folic acid supplementation for the prevention of recurrence of colorectal adenomas: metaanalysis of interventional trials. *Medical oncology (Northwood, London, England)* (2010) 27(3):915-8. Epub 2009/09/17. doi: 10.1007/s12032-009-9306-9. PubMed PMID: 19757214.

78. van Dijk M, Pot GK. The effects of nutritional interventions on recurrence in survivors of colorectal adenomas and cancer: a systematic review of randomised controlled trials. *European journal of clinical nutrition* (2016) 70(5):566-73. Epub 2016/01/14. doi: 10.1038/ejcn.2015.210. PubMed PMID: 26757838.

79. Fife J, Raniga S, Hider PN, Frizelle FA. Folic acid supplementation and colorectal cancer risk: a meta-analysis. *Colorectal disease : the official journal of the Association of Coloproctology of Great Britain and Ireland* (2011) 13(2):132-7. Epub 2009/10/30. doi: 10.1111/j.1463-1318.2009.02089.x. PubMed PMID: 19863600.

80. Qin T, Du M, Du H, Shu Y, Wang M, Zhu L. Folic acid supplements and colorectal cancer risk: meta-analysis of randomized controlled trials. *Scientific reports* (2015) 5:12044. Epub 2015/07/02. doi: 10.1038/srep12044. PubMed PMID: 26131763; PubMed Central PMCID: PMCPMC4487230.

81. Myung SK, Ju W, Cho B, Oh SW, Park SM, Koo BK, et al. Efficacy of vitamin and antioxidant supplements in prevention of cardiovascular disease: systematic review and meta-analysis of randomised controlled trials. *BMJ (Clinical research ed)* (2013) 346:f10. Epub 2013/01/22. doi: 10.1136/bmj.f10. PubMed PMID: 23335472; PubMed Central PMCID: PMCPMC3548618.

82. Li Y, Huang T, Zheng Y, Muka T, Troup J, Hu FB. Folic Acid Supplementation and the Risk of Cardiovascular Diseases: A Meta-Analysis of Randomized Controlled Trials. *Journal of the American Heart Association* (2016) 5(8). Epub 2016/08/17. doi: 10.1161/jaha.116.003768. PubMed PMID: 27528407; PubMed Central PMCID: PMCPMC5015297.

83. Qin X, Huo Y, Langman CB, Hou F, Chen Y, Matossian D, et al. Folic acid therapy and cardiovascular disease in ESRD or advanced chronic kidney disease: a meta-analysis. *Clinical journal of the American Society of Nephrology : CJASN* (2011) 6(3):482-8. Epub 2010/11/23. doi: 10.2215/cjn.05310610. PubMed PMID: 21088292; PubMed Central PMCID: PMCPMC3082404.

84. Qin X, Xu M, Zhang Y, Li J, Xu X, Wang X, et al. Effect of folic acid supplementation on the progression of carotid intima-media thickness: a meta-analysis of randomized controlled trials. *Atherosclerosis* (2012) 222(2):307-13. Epub 2012/01/03. doi: 10.1016/j.atherosclerosis.2011.12.007. PubMed PMID: 22209480.

85. Qin X, Fan F, Cui Y, Chen F, Chen Y, Cheng X, et al. Folic acid supplementation with and without vitamin B6 and revascularization risk: a meta-analysis of randomized controlled trials. *Clinical nutrition (Edinburgh, Scotland)* (2014) 33(4):603-12. Epub 2014/01/28. doi: 10.1016/j.clnu.2014.01.006. PubMed PMID: 24461473.

86. McRae MP. High-dose folic acid supplementation effects on endothelial function and blood pressure in hypertensive patients: a meta-analysis of randomized controlled clinical trials. *Journal of chiropractic medicine* (2009) 8(1):15-24. Epub 2009/08/04. doi: 10.1016/j.jcm.2008.09.001. PubMed PMID: 19646382; PubMed Central PMCID: PMCPMC2697578.

87. Yi X, Zhou Y, Jiang D, Li X, Guo Y, Jiang X. Efficacy of folic acid supplementation on endothelial function and plasma homocysteine concentration in coronary artery disease: A meta-analysis of randomized controlled trials. *Experimental and therapeutic medicine* (2014) 7(5):1100-10. Epub 2014/06/19. doi: 10.3892/etm.2014.1553. PubMed PMID: 24940394; PubMed Central PMCID: PMCPMC3991512.

88. Tabrizi R, Lankarani KB, Akbari M, Naghibzadeh-Tahami A, Alizadeh H, Honarvar B, et al. The effects of folate supplementation on lipid profiles among patients with metabolic diseases: A systematic review and meta-analysis of randomized controlled trials. *Diabetes & metabolic syndrome* (2018) 12(3):423-30. Epub 2017/12/28. doi: 10.1016/j.dsx.2017.12.022. PubMed PMID: 29279272.

89. Huo Y, Qin X, Wang J, Sun N, Zeng Q, Xu X, et al. Efficacy of folic acid supplementation in stroke prevention: new insight from a meta-analysis. *International journal of clinical practice* (2012) 66(6):544-51. Epub 2012/05/23. doi: 10.1111/j.1742-1241.2012.02929.x. PubMed PMID: 22607506.

90. Lee M, Hong K-S, Chang S-C, Saver JL. Efficacy of homocysteine lowering therapy with folic acid in stroke prevention: a meta-analysis. *Stroke; a journal of cerebral circulation* (2010) 41(6):1205-12. doi: 10.1161/STROKEAHA.109.573410. PubMed PMID: PMC2909661.

91. Tian T, Yang KQ, Cui JG, Zhou LL, Zhou XL. Folic Acid Supplementation for Stroke Prevention in Patients With Cardiovascular Disease. *The American journal of the medical sciences* (2017) 354(4):379-87. Epub 2017/10/29. doi: 10.1016/j.amjms.2017.05.020. PubMed PMID: 29078842.

92. Wang X, Qin X, Demirtas H, Li J, Mao G, Huo Y, et al. Efficacy of folic acid supplementation in stroke prevention: a meta-analysis. *Lancet (London, England)* (2007) 369(9576):1876-82. Epub 2007/06/05. doi: 10.1016/s0140-6736(07)60854-x. PubMed PMID: 17544768.

93. Zhao M, Wu G, Li Y, Wang X, Hou FF, Xu X, et al. Meta-analysis of folic acid efficacy trials in stroke prevention: Insight into effect modifiers. *Neurology* (2017) 88(19):1830-8. Epub 2017/04/14. doi: 10.1212/wnl.0000000000003909. PubMed PMID: 28404799.

94. Hsu CY, Chiu SW, Hong KS, Saver JL, Wu YL, Lee JD, et al. Folic Acid in Stroke Prevention in Countries without Mandatory Folic Acid Food Fortification: A Meta-Analysis of Randomized Controlled Trials. *Journal of stroke* (2018) 20(1):99-109. Epub 2018/02/07. doi: 10.5853/jos.2017.01522. PubMed PMID: 29402063; PubMed Central PMCID: PMCPMC5836580.

95. de Bree A, van Mierlo LA, Draijer R. Folic acid improves vascular reactivity in humans: a meta-analysis of randomized controlled trials. *The American journal of clinical nutrition* (2007) 86(3):610-7. Epub 2007/09/08. doi: 10.1093/ajcn/86.3.610. PubMed PMID: 17823424.

96. Wald DS, Kasturiratne A, Simmonds M. Effect of folic acid, with or without other B vitamins, on cognitive decline: meta-analysis of randomized trials. *The American journal of medicine* (2010) 123(6):522-7.e2. Epub 2010/06/24. doi: 10.1016/j.amjmed.2010.01.017. PubMed PMID: 20569758.

97. Schefft C, Kilarski LL, Bschor T, Kohler S. Efficacy of adding nutritional supplements in unipolar depression: A systematic review and meta-analysis. *European neuropsychopharmacology : the journal of the European College of Neuropsychopharmacology* (2017) 27(11):1090-109. Epub 2017/10/11. doi: 10.1016/j.euroneuro.2017.07.004. PubMed PMID: 28988944.

98. Ranganathan LN, Ramaratnam S. Vitamins for epilepsy. *Cochrane Database of Systematic Reviews* (2005) (2). doi: 10.1002/14651858.CD004304.pub2. PubMed PMID: CD004304.

99. Taylor MJ, Carney SM, Goodwin GM, Geddes JR. Folate for depressive disorders: systematic review and meta-analysis of randomized controlled trials. *Journal of psychopharmacology (Oxford, England)* (2004) 18(2):251-6. Epub 2004/07/21. doi: 10.1177/0269881104042630. PubMed PMID: 15260915.

100. Roberts E, Carter B, Young AH. Caveat emptor: Folate in unipolar depressive illness, a systematic review and meta-analysis. *Journal of psychopharmacology (Oxford, England)* (2018) 32(4):377-84. Epub 2018/02/15. doi: 10.1177/0269881118756060. PubMed PMID: 29442609.

101. Akbari M, Tabrizi R, Lankarani KB, Heydari ST, Karamali M, Kashanian M, et al. The Effects of Folate Supplementation on Diabetes Biomarkers Among Patients with Metabolic Diseases: A Systematic Review and Meta-Analysis of Randomized Controlled Trials. *Hormone and metabolic research = Hormon- und Stoffwechselforschung = Hormones et metabolisme* (2018) 50(2):93-105. Epub 2018/01/18. doi: 10.1055/s-0043-125148. PubMed PMID: 29342488.

102. Zhao JV, Schooling CM, Zhao JX. The effects of folate supplementation on glucose metabolism and risk of type 2 diabetes: a systematic review and meta-analysis of randomized controlled trials. *Annals of epidemiology* (2018) 28(4):249-57.e1. Epub 2018/03/05. doi: 10.1016/j.annepidem.2018.02.001. PubMed PMID: 29501221.

103. Sudchada P, Saokaew S, Sridetch S, Incampa S, Jaiyen S, Khaithong W. Effect of folic acid supplementation on plasma total homocysteine levels and glycemic control in patients with type 2 diabetes: a systematic review and meta-analysis. *Diabetes research and clinical practice* (2012) 98(1):151-8. Epub 2012/06/26. doi: 10.1016/j.diabres.2012.05.027. PubMed PMID: 22727498.

104. Shea B, Swinden MV, Tanjong Ghogomu E, Ortiz Z, Katchamart W, Rader T, et al. Folic acid and folinic acid for reducing side effects in patients receiving methotrexate for rheumatoid arthritis. *The Cochrane database of systematic reviews* (2013) (5):Cd000951. Epub 2013/06/04. doi: 10.1002/14651858.CD000951.pub2. PubMed PMID: 23728635.

105. Berti C, Fekete K, Dullemeijer C, Trovato M, Souverein OW, Cavelaars A, et al. Folate intake and markers of folate status in women of reproductive age, pregnant and lactating women: a meta-analysis. *Journal of nutrition and metabolism* (2012) 2012:470656. Epub 2012/10/02. doi: 10.1155/2012/470656. PubMed PMID: 23024859; PubMed Central PMCID: PMCPMC3449134.

106. Clarke R, Armitage J. Vitamin supplements and cardiovascular risk: review of the randomized trials of homocysteine-lowering vitamin supplements. *Seminars in thrombosis and hemostasis* (2000) 26(3):341-8. Epub 2000/09/30. doi: 10.1055/s-2000-8101. PubMed PMID: 11011852.

107. Dose-dependent effects of folic acid on blood concentrations of homocysteine: a meta-analysis of the randomized trials. *The American journal of clinical nutrition* (2005) 82(4):806-12. Epub 2005/10/08. doi: 10.1093/ajcn/82.4.806. PubMed PMID: 16210710.

108. Duffy ME, Hoey L, Hughes CF, Strain JJ, Rankin A, Souverein OW, et al. Biomarker responses to folic acid intervention in healthy adults: a meta-analysis of randomized controlled trials. *The American journal of clinical nutrition* (2014) 99(1):96-106. Epub 2013/11/15. doi: 10.3945/ajcn.113.062752. PubMed PMID: 24225357.
